# Systematic review of the relationships between physical activity and health indicators in the early years (0-4 years)

**DOI:** 10.1186/s12889-017-4860-0

**Published:** 2017-11-20

**Authors:** Valerie Carson, Eun-Young Lee, Lyndel Hewitt, Cally Jennings, Stephen Hunter, Nicholas Kuzik, Jodie A. Stearns, Stephanie Powley Unrau, Veronica J. Poitras, Casey Gray, Kristi B. Adamo, Ian Janssen, Anthony D. Okely, John C. Spence, Brian W. Timmons, Margaret Sampson, Mark S. Tremblay

**Affiliations:** 1 Faculty of Physical Education and Recreation, University of Alberta, Edmonton, AB T6G 2H9 Canada; 2 Early Start Research Institute, Faculty of Social Sciences, University of Wollongong, Wollongong, NSW 2522 Australia; 3 Healthy Active Living and Obesity Research Group, Children’s Hospital of Eastern Ontario Research Institute, Ottawa, ON K1H 8L1 Canada; 4 School of Human Kinetics, Faculty of Health Sciences, University of Ottawa, Ottawa, ON K1N 1A2 Canada; 5 School of Kinesiology and Health Studies, and Department of Public Health Sciences, Queen’s University, Kingston, ON K7L 3N6 Canada; 6 Child Health & Exercise Medicine Program, Department of Pediatrics, McMaster University, Hamilton, ON L8S 4K1 Canada; 7 Library and Media Services, Children’s Hospital of Eastern Ontario, Ottawa, ON K1H 8L1 Canada

**Keywords:** Physical activity, Prone position, Adiposity, Motor development, Psychosocial health, Cognitive development, Fitness, Skeletal health, Cardiometabolic health, Injury, Early years, Infants, Toddlers, Preschoolers

## Abstract

**Background:**

Given the rapid development during the early years (0-4 years), an understanding of the health implications of physical activity is needed. The purpose of this systematic review was to examine the relationships between objectively and subjectively measured physical activity and health indicators in the early years.

**Methods:**

Electronic databases were originally searched in April, 2016. Included studies needed to be peer-reviewed, written in English or French, and meet a priori study criteria. The population was apparently healthy children aged 1 month to 59.99 months/4.99 years. The intervention/exposure was objectively and subjectively measured physical activity. The comparator was various volumes, durations, frequencies, patterns, types, and intensities of physical activity. The outcomes were health indicators ranked as critical (adiposity, motor development, psychosocial health, cognitive development, fitness) and important (bone and skeletal health, cardiometabolic health, and risks/harm). The Grading of Recommendations Assessment, Development, and Evaluation (GRADE) framework was used to assess the quality of evidence for each health indicator by each study design.

**Results:**

Ninety-six studies representing 71,291 unique participants from 36 countries were included. Physical activity interventions were consistently (>60% of studies) associated with improved motor and cognitive development, and psychosocial and cardiometabolic health. Across observational studies, physical activity was consistently associated with favourable motor development, fitness, and bone and skeletal health. For intensity, light- and moderate-intensity physical activity were not consistently associated with any health indicators, whereas moderate- to vigorous-intensity, vigorous-intensity, and total physical activity were consistently favourably associated with multiple health indicators. Across study designs, consistent favourable associations with health indicators were observed for a variety of types of physical activity, including active play, aerobic, dance, prone position (infants; ≤1 year), and structured/organized. Apart from ≥30 min/day of the prone position for infants, the most favourable frequency and duration of physical activity was unclear. However, more physical activity appeared better for health. Evidence ranged from “very low” to “high” quality.

**Conclusions:**

Specific types of physical activity, total physical activity, and physical activity of at least moderate- to vigorous-intensity were consistently favourably associated with multiple health indicators. The majority of evidence was in preschool-aged children (3-4 years). Findings will inform evidence-based guidelines.

**Electronic supplementary material:**

The online version of this article (10.1186/s12889-017-4860-0) contains supplementary material, which is available to authorized users.

## Background

The health benefits of physical activity, in particular moderate- to vigorous-intensity physical activity (MVPA), have been frequently studied in school-aged children and youth (5-17 years) as well as adults (≥18 years) [1–4]. Accordingly, global recommendations on the amount of MVPA recommended for health benefits in these age groups exists [5]. In contrast, less research has focused on the health benefits of physical activity in the early years (0-4 years). Given that the early years are a critical and rapid period of physical, cognitive, social, and emotional development [6], determining the dose (e.g., frequency, intensity, time/duration, type) of physical activity needed for healthy growth and development is of great importance.

To better understand the dose of physical activity needed in the early years, in 2012 Timmons and colleagues conducted a systematic review that examined the relationship between physical activity and multiple health indicators in this age group [7]. Favourable associations between physical activity and some aspects of health, including adiposity, bone and skeletal health, motor skill development, psychosocial health, cognitive development, and cardiometabolic health, were reported [7]. However, within this review, cross-sectional studies were excluded a priori; consequently, only 22 studies were identified and limited information on the dose of physical activity required for health benefits was found [7].

The previous systematic review by Timmons and colleagues helped inform the first *Canadian Physical Activity Guidelines for the Early Years* [8]. Given the limited information on the dose of physical activity required for good health, guideline formation was influenced by expert opinion, international harmonization, and stakeholder input [8]. The guidelines state that for healthy growth and development, infants (<1 year) should be physically active several times daily, and toddlers (1-2 years) and preschoolers (3-4 years) should accumulate at least 180 min per day of physical activity at any intensity spread throughout the day and progress to 60 min per day of energetic play by 5 years of age [8]. These recommendations align with physical activity recommendations in Australia [9] and the United Kingdom [10].

Since the dissemination of physical activity guidelines for children of the early years in Australia, Canada, and the United Kingdom [8–10], a number of new studies have examined physical activity in this age group, primarily in preschool-aged children [11]. However, due to several gaps and limitations in the literature, it remains unclear whether children in the early years are sufficiently active for good health [11, 12]. For example, no clear benchmark exists for the appropriate dose of physical activity in infants; limited research has been conducted with toddlers [13–15]; and estimates of the proportion of preschool-aged children meeting the physical activity guidelines vary considerably (27%-100%) [11]. This variation is partly due to different methodologies used across studies, and in particular different cut-points for light-intensity physical activity (LPA) [11]. Despite differences in cut-points used, most of the physical activity in preschool-aged children appears to be of low-intensity [11, 16, 17]. Currently, the specific frequency, intensity, duration, and type of physical activity required for good health in the early years remains unclear.

To ensure physical activity guidelines are reflective of the most up-to-date scientific knowledge, it is important to revisit and update the available evidence [18]. As studies with cross-sectional designs were excluded in the 2012 review [7] that informed the current Canadian guidelines, all available evidence was not originally captured. Causality cannot be determined with cross-sectional studies. However, given the limited evidence, cross-sectional studies may help to expand the current understanding of the relationships between physical activity and health in the early years. Since the 2012 review, other systematic reviews have been completed but they have focused on specific types of physical activity (e.g., outdoor play, structured physical activity) or specific health indicators (e.g., motor development, cognitive development, psychosocial health) [19–23], and three of the five reviews only included preschool-aged children [20, 22, 23]. To our knowledge, no systematic review has been conducted that comprehensively examined the relationships between subjectively and objectively measured physical activity and a broad range of health indicators in infants, toddlers, and preschoolers across study designs. Therefore, the purpose of this systematic review was to examine the associations between objectively and subjectively measured physical activity and health indicators in the early years across all study designs. To help inform guideline updates or development, an additional purpose was to determine what dose of physical activity is associated with health indicators in children of the early years.

## Methods

### Protocol and registration

This systematic review was registered with the International Prospective Register of Systematic Reviews (PROSPERO; Registration no. CRD42016035937; available from: https://www.crd.york.ac.uk/PROSPERO/display_record.php?ID=CRD42016035937). It was conducted and reported following the Preferred Reporting Items for Systematic Reviews and Meta-Analyses (PRISMA) statement for reporting systematic reviews and meta-analyses [24].

### Eligibility criteria

For a study to be included in this review, it had to be peer-reviewed, published, written in English or French, and meet a priori (i.e., before database searches and screening) determined Population, Intervention, Comparison, and Outcome (PICO) study criteria [25]. Conference abstracts and grey literature were not eligible because they may not be subject to the same peer-review rigour. However, preliminary results from registered clinical trials were eligible.

#### Population

The population was apparently healthy (i.e., general population, including samples of overweight/obese children but not samples of children exclusively with a diagnosed medical condition) young children (mean age: 1 month-59.99 months/4.99 years). Where an age range was reported instead of a mean, samples with a lower limit of 1 month-59.99 months/4.99 years and an upper limit of <6 years were eligible for inclusion. If a mean age or age range was not reported, samples described as infants, toddlers, and/or preschoolers were included. For longitudinal or experimental study designs, the age criterion applied to at least one measurement time point of the exposure. For feasibility (i.e., staff and funding restrictions and overall project timelines) and to maximize the generalizability of findings, experimental studies were required to have a minimum sample size of 15 participants in at least one intervention group and observational studies were required to have a minimum sample size of 100 participants. Setting minimum sample size inclusion criteria a priori is consistent with a similar systematic review in school-aged children and youth [2]; however, more lenient cut-offs were chosen a priori in the present review because it was anticipated the volume of research was lower in the early years age group. Age subgroups were defined as 1.0-12.99 months (≤1.0 year) for infants, 13.0-35.99 months (1.1-2.99 years) for toddlers, and 36.0-59.99 months (3.0-4.99 years) for preschoolers.

#### Intervention (exposure)

The interventions were volumes, durations, frequencies, patterns, types, and intensities of physical activity. For this review, physical activity was defined as any bodily movement generated by skeletal muscles that results in energy expenditure above resting levels [26]. “Prone position” or “tummy time” in infants, and “outdoor time” in any age group, were considered eligible physical activity exposures. Total energy expenditure measured by doubly labelled water or direct/indirect calorimetry was not considered an eligible exposure because it includes resting metabolic rate and the thermic effect of food in addition to activity energy expenditure [27]; however, activity energy expenditure measured by these methods was eligible. Physical activity could be measured objectively (e.g., accelerometer, direct observation) or subjectively (e.g., proxy-report). For experimental studies, interventions had to target physical activity exclusively with no other health behaviours (e.g., physical activity and diet or physical activity and sedentary behaviour), but were not required to have reported a measured change in physical activity.

#### Comparison

The comparators were volumes, durations, frequencies, patterns, types, and intensities of physical activity. A comparator or control group was not required.

#### Outcomes (health indicators)

The outcomes were eight health indicators chosen by the review team and collaborators based on the scientific literature to reflect physical, social, and cognitive health. The review team and collaborators ranked the eight health indicators as “critical” or “important” in line with the Grading of Recommendations Assessment, Development, and Evaluation (GRADE) framework [28, 29]. *Critical* health indicators included: adiposity (e.g., overweight, obesity, body mass index [BMI], skinfold thickness, body fat), motor development (e.g., gross motor skills, fine motor skills, locomotor and object control skills), psychosocial health (e.g., self-efficacy, self-esteem, prosocial behaviour, aggression, social functioning, depressive symptoms, anxiety symptoms, quality of life), cognitive development (e.g., language development, attention, executive functioning), and fitness (e.g., cardiovascular fitness, musculoskeletal fitness). *Important* health indicators included: bone and skeletal health (e.g., bone mineral density, bone mineral content, skeletal area, Vitamin D), cardiometabolic health (e.g., blood pressure, insulin resistance, blood lipids), and risks/harm (e.g., injury, plagiocephaly).

### Information sources and search strategy

The search strategies for this review were developed and peer-reviewed by two librarians with expertise in systematic reviews. The following databases were searched between April 14 and 28, 2016 (the full MEDLINE search was run at this time and again in July to ensure currency): SPORTDiscus (dates of coverage not stated), MEDLINE In-Process & Other Non-Indexed Citations and Ovid MEDLINE (1946-July 29, 2016), EMBASE (1974 to 2016 April Week 4), PsycINFO (1806 to April Week 4 2016), and Cochrane Central Register of Controlled Trials (CENTRAL) (February 2016 issue). No date or study design limits were included (see Additional file 1 for the complete search strategies). As more than 6 months had passed since the initial full search, a partial search update was conducted in all databases on November 1, 2016, to capture any randomized controlled trials (RCTs) or clustered RCTs that included “critical” health indicators. A partial search update rather than a full update was conducted because of logistical reasons (i.e., staff and funding restrictions and overall project timelines). Furthermore, a large volume of observational studies had already been captured, so it was a priority to focus on studies with designs that have the potential to provide the highest quality of evidence to inform review findings and guideline formation.

All records retrieved from the database searches were imported into Reference Manager Software (Version 11; Thompson Reuters, San Francisco, CA, USA), and duplicate records were removed by employing a two-step strategy. Specifically, duplicates were first identified automatically in Reference Manager; one member of the review team then manually checked and removed additional duplicates where appropriate. After de-duplication, records were imported into Distiller SR Software (Evidence Partners, Ottawa, ON, Canada) for screening. First, titles and abstracts were screened by two independent reviewers; if a record was included by at least one reviewer, the record was obtained for further screening. Second, full-text articles were obtained and screened by two independent reviewers. Agreement between reviewers was required for a study to be included or excluded. Discrepancies that could not be resolved by the two independent reviewers were resolved by discussions with a third reviewer or with the review team if needed.

The reference lists of relevant reviews identified during screening were also checked to see if any additional relevant studies could be identified. To capture registered clinical trials, two trial registries (https://clinicaltrials.gov and http://www.who.int/ictrp/en/) were searched on February 1, 2017, using search terms for physical activity and the early years age group. This final search was to detect any large studies that were in progress and could potentially overturn findings. If found, this pending new evidence would have been included in the discussion.

### Data extraction

Descriptive study characteristics as well as information regarding the exposure, outcome, and results were extracted in Microsoft Excel for each included study. For the results, where applicable, information was extracted from both unadjusted models and the most fully adjusted model. Furthermore, a finding was deemed to be statistically significant when *p* < 0.05 was reported, even if statistical significance was defined differently in a study. One reviewer completed data extraction for each study and a second reviewer checked the extracted data. A third reviewer then checked all extracted results.

### Quality assessment

The quality of evidence assessment for each included study design within each health indicator was guided by the GRADE framework [30]. Quality of evidence reflects the level of confidence in the estimated effects. Detailed information on GRADE methodology can be found elsewhere [30]. Briefly, five assessment criteria (risk of bias, inconsistency, indirectness, imprecision, other [e.g., dose-response evidence]) were used to rate quality of evidence as “high”, “moderate”, “low”, or “very low”. Quality of evidence ratings started at “high” for RCTs and “low” for all other experimental and observational studies. The quality of evidence could be downgraded for any study design due to limitations associated with the five assessment criteria. The review team decided a priori that if the only identified sources of bias were selection bias due to the use of a convenience sample or performance bias due to lack of intervention/control group blinding, the quality of evidence would not be downgraded because of the risk of bias. If no limitations were identified, the quality of evidence from non-randomized and observational study designs could be upgraded if large effect sizes or evidence of a dose-response gradient were reported. Since dose-response evidence could not be determined for cross-sectional studies, observations of a gradient of higher exposure with higher/lower outcome were considered a reason to upgrade the quality of evidence associated with this study design [29].

Risk of bias was the only criterion out of the five assessment criteria that was first assessed at the individual study level. The Cochrane risk of bias assessment was used for experimental studies [31]. For observational studies, the risk of selection bias, performance bias, selective reporting bias, detection bias, attrition bias, and other biases (e.g., inadequate control for key confounders) was assessed [32]. For all studies, risk of bias was assessed by one reviewer and checked by two other reviewers. Overall quality of evidence was evaluated by one reviewer and verified by the larger review team, including two members with expertise in systematic review methodology.

### Data analysis

Two members of the review team with experience in conducting meta-analyses assessed the data for each health indicator to determine if any of the data was sufficiently homogenous with regard to statistical, clinical, and methodological characteristics for meta-analyses. Due to high levels of heterogeneity in study design and measured outcomes, only one meta-analysis was possible for four studies that included adiposity as a health indicator [33–36]. Change (post-intervention minus baseline) values from studies were entered into Review Manager Software 5.3 (The Cochrane Collaboration, Copenhagen, Denmark). When necessary, standard deviations of change were calculated based on other available statistics in accordance with the Cochrane Handbook for Systematic Reviews of Interventions [31]. Additionally, one study [37] only presented results by sex-specific sub-groups, and thus had to be entered into the meta-analysis accordingly. Based on the subjectively assessed heterogeneity of the interventions, random-effects models were used to calculate the weighted mean difference according to the DerSimonian and Laird method [38, 39]. Due to the small number of studies included in the meta-analysis, sensitivity analyses and/or sub-group analyses were not possible.

A narrative synthesis was also conducted for all included studies. Results were first synthesized by health indicator and study design then further synthesized by intensity or type of physical activity. For fitness and cardiometabolic health, results were also synthesized by different dimensions of the indicator (i.e., cardiorespiratory fitness and other fitness measures; blood pressure, cholesterol, and triglycerides). Finally, a sub-group analysis was conducted to examine frequency and duration of physical activity. Since not all studies reported on frequency and duration, data were synthesized across health indicators but examined separately for experimental and observational study designs. For observational study designs, frequency and duration data were also synthesized for intensity and type of physical activity. When multiple associations were examined (e.g., physical activity and BMI and physical activity and waist circumference or sex-stratified analyses between physical activity and BMI), a study was classified in one of four mutually exclusive groups: 1) “favourable” if at least one favourable but no unfavourable associations were observed, 2) “unfavourable” if at least one unfavourable but no favourable associations were observed, 3) “null” if no favourable or unfavourable associations were observed, and 4) “mixed” if both favourable and unfavourable or favourable, unfavourable and null associations were all observed. Within the narrative analysis, all studies were weighted equally. Finally, unless otherwise stated, findings are based on samples classified as preschool-aged children.

## Results

### Description of studies

**Fig. 1:**
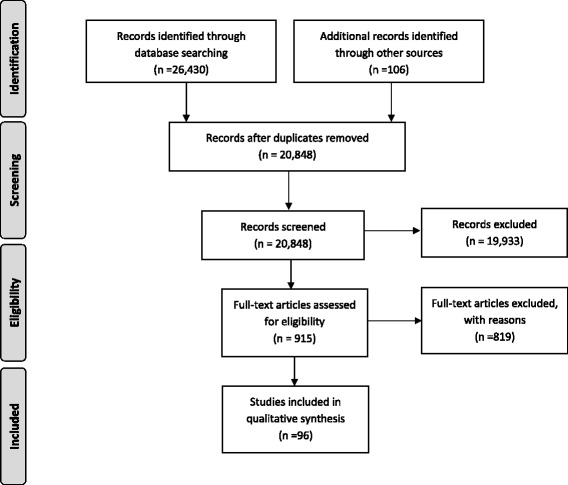
Flow diagram for the identification, screening, eligibility, and inclusion of studies [24]

After de-duplication, 20,848 titles and abstracts and 915 full-text articles were screened (see Fig. 1). It was determined that 96 studies (87 unique samples) met the inclusion criteria. Of the 96 studies, 4 were identified through the MEDLINE update of the full search. No additional studies were identified in the partial update search or the trial registry searches. Reasons for excluding full-text articles included: not original research (e.g., review; *n* = 116), non-English language or non-French language (*n* = 4), ineligible age (*n* = 321), special population (*n* = 19), no measure of physical activity (*n* = 155), no measure of a health indicator of interest/did not assess the association between physical activity and health indicator of interest (*n* = 98), sample size (*n* = 48), intervention did not exclusively target physical activity (*n* = 45), and other (e.g., physical activity was a covariate, not human participants; *n* = 13). Some full-text articles were excluded for multiple reasons. Additionally, nine full-text articles could not be located so these records were excluded.

The 96 studies involved 86,040 participants (71,291 from unique samples) from 36 different countries. An experimental study design was used in 24 studies, including RCTs (*n* = 8), clustered RCTs (*n* = 4), non-randomized intervention (*n* = 9), and cross-over trial (*n* = 3) designs. An observational study design was used in the remaining 72 studies, including longitudinal (*n* = 7), longitudinal with additional cross-sectional analyses (*n* = 5), case-control (*n* = 4), case cross-over (*n* = 1), and cross-sectional (*n* = 55) designs. Out of the 96 studies, 80 were classified as preschool samples, two as toddler samples, 13 as infant samples, and one as both infant and toddler samples (i.e., 0.1-2.9 years).

Physical activity was measured objectively in 38 studies, primarily by accelerometers; 10 studies used direct observation, heart rate monitors, pedometers, and/or doubly labelled water (i.e., activity energy expenditure). Physical activity was measured subjectively in 48 studies by proxy-report questionnaire, log, or interview. Five studies used both objective and subjective measures of physical activity. For 15 studies that included physical activity interventions, physical activity was not measured but these studies were included in the review because the intervention targeted physical activity exclusively. The types of physical activity included in both observational and experimental studies were: active play, active transportation, aerobic, biking, dance, home-based, exercise play, indoor, leisure, outdoor, passive cycling, prone position, rough-and-tumble play, sport, structured/organized, walking, and weight bearing. Further information on the study design, sample, exposure, outcome, and main findings for all individual studies are summarized in Tables S1 to S8 in Additional file 2. It should be noted that the number of studies summed across study designs and across health indicators is more than 96 because 20 studies included more than one health indicator of interest, and five studies presented both longitudinal and cross-sectional findings.

### Data synthesis

#### Adiposity

**Fig. 2:**
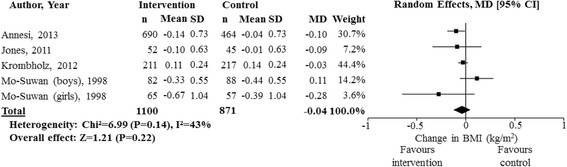
Meta-analysis of the effect of physical activity interventions on body mass index

The association between physical activity and adiposity was examined in 57 studies (49 unique samples; see Table 1 and Table S1 in Additional file 2). Meta-analyses of four studies, including three clustered RCTs [33–35] and one non-randomized intervention [36], involving a total of 1100 participants, found no significant differences between intervention and control groups for BMI (weighted mean difference = −0.04 kg/m^2^; 95% confidence interval = −0.12, 0.03; I^2^ = 43%; *n* = 1971; see Fig. 2).

**Table 1:** The relationship between physical activity and adiposity

# of studies	Design	Quality assessment					# of participants	Absolute effect	Quality
		Risk of bias	Inconsistency	Indirectness	Imprecision	Other			
Mean baseline age ranged from 41 weeks-59.6 months; where mean age was not reported, baseline age ranged from 2 weeks- <6 years. Data were collected by RCT, clustered RCT, non-randomized intervention, longitudinal with up to 4-year follow-up, case-control, and cross-sectional study designs. Adiposity was assessed objectively by BMI, weight-for-height z-score, BMI z-score (CDC, WHO, other country-specific reference data), weight/height^3^, weight percentiles, weight status (CDC, WHO, IOTF, Kaup index, country-specific reference data, BMI > 18, BMI percentile ≥95, ≥85th and ≥95th percentiles), waist circumference (absolute, percentile), hip circumference, waist-to-hip ratio, waist circumference z-score (Netherlands reference data), waist circumference-for-age z-score, sum of skinfolds, triceps skinfold thickness, body fat % (bioelectrical impedance, dual-energy X-ray absorptiometry), fat mass index (dual energy X-ray absorptiometry, air-displacement plethysmography), fat free mass index (dual energy X-ray absorptiometry, air-displacement plethysmography), fat mass (dual energy X-ray absorptiometry, air-displacement plethysmography), fat free mass (dual energy X-ray absorptiometry), % fat mass, trunk fat mass index, lean mass index (dual-energy X-ray absorptiometry), and subjectively by weight status (CDC ≥85th percentile). In 2 studies, it was unclear whether weight status (CDC ≥85th percentile) or BMI was measured objectively or subjectively.									
1	RCT^a^	No serious risk of bias	No serious inconsistency	Very serious indirectness^b^	No serious imprecision	None	161	The **PA intervention (PA recommendations from nurse)** was *favourably* associated with improved adiposity (*sum of 4 skinfolds but not % overweight, waist circumference, hip circumference, or body fat %*) in **1 study** [40].	LOW^c^
4	Clustered RCT^d^	Serious risk of bias^e^	No serious inconsistency	Serious indirectness^f^	No serious imprecision	None	3028	The **PA interventions (structured/organized PA)** were *favourably* associated with adiposity in **1 study** [34]. The **PA interventions (structured/organized PA, aerobic PA, or government-led PA program)** were *not* associated with adiposity in **3 studies** [33, 35, 41].	LOW^g^
2	Non-randomized intervention^h^	Serious risk of bias^i^	No serious inconsistency	No serious indirectness	No serious imprecision	None	640	The **PA interventions (structured/organized PA)** were *not* associated with adiposity in **2 studies** [36, 42].	VERY LOW^j^
7	Longitudinal^k^	Serious risk of bias^l^	No serious inconsistency	No serious indirectness	No serious imprecision	Dose-response gradient^m^	2441	**TPA** was *favourably* associated with adiposity (*change in weight-for-height z-score but not waist circumference-for-age z-score in 1 study*) in **2 studies** [43, 45] and *not* associated with adiposity in **2 studies** [46, 49]. **MVPA** was *favourably* associated with adiposity (*fat free mass but not BMI, fat mass, or % fat mass in 1 study*) in **1 study** [49]. **VPA** was *not* associated with adiposity in **1 study** [48]. **Activity energy expenditure** was *favourably* (*fat free mass*), *unfavourably* (*BMI, fat mass*), and *not* (*% fat mass*) associated with adiposity in **1 study** [49]. **Aerobic PA** was *favourably* associated with adiposity (*baseline PA only not change in PA*) in **1 study** [44]. **Home-based PA** was *not* associated with adiposity in **1 study** [47]. **Leisure PA** was *not* associated with adiposity in **1 study** [44]. **Structured/organized PA** was *not* associated with adiposity in **2 studies** [44, 47].	VERY LOW^n^
3	Case-contol^o^	Serious risk of bias^p^	No serious inconsistency	No serious indirectness	No serious imprecision	None	2271	**TPA** was *not* associated with adiposity in **1 study** [51]. **MPA** was *not* associated with adiposity in **1 study** [52]. **VPA** was *not* associated with adiposity in **1 study** [52]. **Outdoor PA** was *favourably* associated with adiposity in **1 study** [51] and *not* associated with adiposity in **1 study** [53].	VERY LOW^q^
40	Cross-sectional^r^	Serious risk of bias^s^	Serious inconsistency^t^	No serious indirectness	No serious imprecision	Exposure/outcome gradient^u^	37,813	**TPA** was *favourably* associated with adiposity (*age 6 months but not 1, 2, 3, and 4 years in 1 study; boys only in 1 study; 95th percentile of vector magnitude and fat free mass index but not BMI, fat mass, or waist circumference and 90th percentile of vector magnitude and % fat mass and fat free mass index but not BMI, fat mass index, or waist circumference in 1 study*) in **6 studies** [55, 56, 60, 61, 63, 64], *unfavourably* associated with adiposity (*BMI z-score but not waist circumference z-score in 1 study and hip circumference but not relative weights, skinfold thicknesses, and waist circumference in 1 study*) in **3 studies** [50, 66, 69], and *not* associated with adiposity in **11 studies** [45, 46, 49, 54, 65, 72, 73, 75, 81, 82, 86]. **LPA** was *favourably* associated with adiposity (*waist circumference z-score but not BMI z-score*) in **1 study** [50], *unfavourably* associated with adiposity (% *body fat and fat mass index but not trunk fat mass index and lean mass index*) in **1 study** [89], and *not* associated with adiposity in **6 studies** [55, 67, 76, 84, 86, 87]. **LPA 5-min bouts** were *not* associated with adiposity in **1 study** [86]. **MPA** was *unfavourably* associated with adiposity in **1 study** [50] and *not* associated with adiposity in **2 studies** [55, 89]. **MVPA** was *favourably* associated with adiposity (*% fat mass but not BMI, fat free mass, fat mass in 1 study; boys only in 1 study; % body fat and fat mass index but not trunk fat mass index or lean mass index in 1 study; % fat mass and fat free mass index but not BMI, fat mass index, or waist circumference in 1 study; girls only and waist circumference at the 90th percentile but not the 10th, 25th, 75th percentiles or BMI z-score or waist circumference in 1 study*) in **6 studies** [49, 54, 55, 60, 88, 89], *unfavourably* associated with adiposity (*boys only and BMI z-score but not waist circumference in 1 study*) in **3 studies** [67, 69, 88], and *not* associated with adiposity in **8 studies** [65, 76, 77, 82, 84–87]. **MVPA 5-min bouts** were *not* associated with adiposity in **1 study** [86]. **VPA** was *favourably* associated with adiposity (*boys only in 1 study; % body fat, fat mass index, trunk fat mass index but not lean mass index in 1 study; fat free mass index but not BMI, fat mass, fat mass index, and waist circumference in 1 study*) in **4 studies** [54, 55, 60, 89], *unfavourably* associated with adiposity in **1 study** [50], and *not* associated with adiposity in **3 studies** [67, 74, 82]. **Activity energy expenditure** was *favourably* (*fat free mass*), *unfavourably* (*BMI*), and *not* (*fat mass, % fat mass*) associated with adiposity in **1 study** [49]. **Indoor PA** was *not* associated with adiposity in **1 study** [81]. **Leisure PA** was *favourably* associated with adiposity (*intermediate* vs. *none but not high* vs. *none*) in **1 study** [59]. **Outdoor PA** was *favourably* associated with adiposity in **1 study** [58] and *not* associated with adiposity in **8 studies** [61, 73, 75, 78–81, 83] **Organized Sport** was *unfavourably* associated with adiposity (*girls only*) in **1 study** [68]. **Structured/organized PA** was *favourably* associated with adiposity in **1 study** [57]. **Active play** was *favourably* associated with adiposity (*weekdays only in 1 study*) in **2 studies** [62, 65] and *not* associated with adiposity in **1 study** [71]. **Active transportation** was *not* associated with adiposity in **1 study** [70].	VERY LOW^v^

BMI: body mass index; CDC: Centers for Disease Control and Prevention; IOTF: International Obesity Task Force; LPA: light-intensity physical activity MPA: moderate-intensity physical activity; MVPA: moderate- to vigorous-intensity physical activity; PA: physical activity; RCT: randomized controlled trial; TPA: total physical activity; VPA: vigorous-intensity physical activity; WHO: World Health Organization

^a^Includes **1 RCT** [40]

^b^The intervention did not result in a significant change in physical activity [40]

^c^Quality of evidence was downgraded from “high” to “low” because of very serious indirectness

^d^Includes **4 clustered RCTs** [33–35, 41]

^e^Unclear whether outcome assessors were blinded to group allocation and unclear if the outcome was objectively measured in 1 study [34]. Large amount of missing data primarily because mean attendance at child care was 48% and it is unknown if the reason for poor attendance was related to adiposity in 1 study [41]. Physical activity was not measured so it is unknown if the intervention resulted in a significant change in physical activity in 1 study [35]

^f^The intervention did not result in a significant change in physical activity in 1 study [41]

^g^Quality of evidence was downgraded from “high” to “low” because of serious risk of bias and serious indirectness

^h^Includes **2 non-randomized interventions** [36, 42]

^i^No control group in 1 study [42]. Physical activity was not measured so it is unknown if the intervention resulted in a significant change in physical activity in 2 studies [36, 42]

^j^Quality of evidence was downgraded from “low” to “very low” because of serious risk of bias

^k^Includes **7 longitudinal studies** [43–49]

^l^Convenience sample was used in 1 study [44]. Psychometric properties unknown for the subjective physical activity measures in 3 studies [44, 45, 47]. Large unexplained loss to follow-up and incomplete data in 1 study [45]. No potential confounders were adjusted for in 2 studies [43, 45]. Potentially inappropriate statistical analysis: one study mutually adjusted for other movement behaviours in the fully adjusted models [49]

^m^A dose-response gradient of higher aerobic PA and MVPA with better adiposity was observed in 2 studies [44, 49]. A dose-response gradient of higher activity energy expenditure was associated with both better and worse adiposity depending on the adiposity measure in 1 study [49]

^n^Quality of evidence was downgraded from “low” to “very low” because of serious risk of bias; because of this limitation, was not upgraded for a dose-response gradient

^o^Includes **3 case-control studies** [51–53]

^p^Psychometric properties unknown for the subjective physical activity measures in 3 studies [51–53]

^q^Quality of evidence was downgraded from “low” to “very low” because of serious risk of bias

^r^Includes **40 cross-sectional studies** [45, 46, 49, 50, 54–89]

^s^Convenience sample was used in 11 studies [54, 56, 62, 63, 67, 69, 76, 77, 85, 86, 88]. Low participation rate in 3 studies [54, 68, 84]. Psychometric properties unknown for the subjective physical activity measure in 15 studies [45, 57, 59, 61–65, 68, 70, 71, 75, 79, 80, 84]. No potential confounders were adjusted for in 19 studies [45, 50, 56, 61, 64–67, 69, 71, 72, 76, 77, 80, 81, 83, 85–87]. Large amount of unexplained missing data or it was unclear if the large amount of missing data was related to adiposity in 9 studies [50, 57, 62, 65, 67, 68, 80, 82, 89]. Physical activity was measured only during child care in 3 studies [58, 60, 82]. Potentially inappropriate statistical analysis: other movement behaviours were mutually adjusted for in the fully adjusted models in 3 studies [49, 55, 89]

^t^Favourable and unfavourable associations between physical activity and adiposity observed across studies

^u^A gradient for higher TPA, MVPA, VPA activity energy expenditure, outdoor PA, and physical education with better adiposity was observed in 6 studies [49, 55, 57, 58, 88, 89]. A gradient for higher activity energy expenditure and LPA, MVPA with worse adiposity was observed in 3 studies [49, 88, 89]

^v^Quality of evidence was downgraded from “low” to “very low” because of serious risk of bias and serious inconsistency; because of this limitation, was not upgraded for an exposure/outcome gradient

In the RCT, the mean sum of four skinfolds was significantly lower in the intervention group whose parents received physical activity recommendations from a nurse when their child was an infant, compared to the control group, who did not receive recommendations [40]. However, no significant group differences were observed for percentage overweight, waist or hip circumference, or body fat percentage. Furthermore, physical activity did not significantly differ between the intervention and the control group [40]. The quality of evidence was downgraded from “high” to “low” because of very serious indirectness (see Table 1).

For the four clustered RCTs, a significant decrease in BMI was observed in the intervention group (structured/organized physical activity plus cognitive-behavioural training and resources) compared to the control group (structured/organized physical activity) in one study [34]. However, no significant differences in adiposity were observed between the intervention (structured/organized physical activity or aerobic physical activity or government-led physical activity program) and the control groups (standard care) in the other three studies [33, 35, 41]. Furthermore, physical activity did not significantly differ between the intervention and the control groups in one study [41]. The quality of evidence was downgraded from “high” to “low” because of a serious risk of bias and serious indirectness (see Table 1).

For the two non-randomized interventions, there were no significant differences in adiposity between intervention (structured/organized physical activity) and control (standard care) groups in one study [36] or from baseline to follow-up in another study (structured/organized physical activity) [42]. The quality of evidence was downgraded from “low” to “very low” because of a serious risk of bias (see Table 1).

Among the seven longitudinal studies, physical activity was favourably associated with adiposity for at least one association in three studies [43–45] and not associated with adiposity in three studies [46–48]; mixed findings were observed in one study [49]. For two of the studies that found some favourable associations, a number of null associations were also observed [44, 50]. One study with favourable findings had an infant sample [45]. In regard to intensity or type of physical activity, at least one favourable association was observed between each of the following physical activity exposures and adiposity: total physical activity (TPA; 2/4 studies), MVPA (1/1 study), and aerobic physical activity (1/1 study). However, primarily null or mixed associations were observed between each of the following physical activity exposures and adiposity: vigorous-intensity physical activity (VPA), activity energy expenditure, home-based physical activity, leisure physical activity, and structured/organized physical activity (see Table 1). The quality of evidence was downgraded from “low” to “very low” because of a serious risk of bias (see Table 1).

For the three case-control studies, physical activity was favourably associated with adiposity in one study [51] and not associated with adiposity in two studies [52, 53]. One study with null findings had an infant and toddler sample [53]. In terms of the intensity or type of physical activity, at least one favourable association was observed between outdoor physical activity and adiposity (1/2 studies). However, primarily null associations were observed between each of the following physical activity exposures and adiposity: TPA, moderate-intensity physical activity (MPA), and VPA (see Table 1). The quality of evidence was downgraded from “low” to “very low” because of a serious risk of bias (see Table 1).

For the 40 cross-sectional studies, physical activity was favourably associated with adiposity for at least one association in 12 studies [54–65], unfavourably associated with adiposity for at least one association in four studies [66–69], and not associated with adiposity in 20 studies [45, 46, 70–87]; mixed findings were observed in four studies [49, 50, 88, 89]. In two of the studies that observed some favourable associations, primarily null associations were observed [55, 64]. One study with favourable findings [64] and one study with null findings [45] had infant samples. Similarly, one study with null findings had a toddler sample [86]. In regard to intensity or type of physical activity, at least one favourable association was observed between each of the following and adiposity: active play (2/3 studies), leisure physical activity (1/1 study), and structured/organized physical activity (1/1 study); and at least one unfavourable association was observed between organized sport and adiposity (1/1 study). However, primarily null or mixed findings were observed between each of the following physical activity exposures and adiposity: TPA, LPA, LPA bouts, MPA, MVPA, MVPA bouts, VPA, activity energy expenditure, active transportation, indoor physical activity, and outdoor physical activity (see Table 1). The quality of evidence was downgraded from “low” to “very low” because of a serious risk of bias and serious inconsistency (see Table 1).

#### Motor development

The association between physical activity and motor development was examined in 23 studies (21 unique samples; see Table 2 and Table S2 in Additional file 2). Among the four RCTs, significant increases in motor development were observed in the intervention groups (planned passive cycling or structured/organized physical activity) compared to the control groups (standard care) in three studies [90–92]. One intervention involved an infant sample [91]. In the fourth study, no significant differences in motor development were observed between the intervention (parents received physical activity recommendations from a nurse when their child was an infant) and control (no recommendations) groups [40]; however, physical activity was not significantly different between groups [40]. The quality of evidence was downgraded from “high” to “low” because of a serious risk of bias and serious indirectness (see Table 2).

**Table 2:** The relationship between physical activity and motor development

# of studies	Design	Quality assessment					# of participants	Absolute effect	Quality
		Risk of bias	Inconsistency	Indirectness	Imprecision	Other			
Mean baseline age ranged from 18.3 weeks-59.79 months; where mean age was not reported, baseline age ranged from 0 months-5 years. Data were collected by RCT, clustered RCT, non-randomized intervention, longitudinal with up to 20-month follow-up, and cross-sectional study designs. Motor development was assessed by fundamental movement skills/motor ability/motor performance/motor development/motor skills/gross-motor development/psychomotor skills (objectively measured; Test of Gross Motor Development – 2, movement assessment battery, Movement Assessment Battery for Children – 2, APM-Inventory, Dutch Second Edition of the Bayley Scales of Infant and Toddler – 3, Motoriktestfürvier-bissechsjährige Kinder 4-6; 12-m run, standing long jump, Motor Test Battery 3-7, Alberta Infant Motor Scales, neurological examination technique for toddler-age, Children’s Activity and Movement in Preschool Study Motor Skill Protocol, Comprehensive Developmental Inventory for Infants and Toddlers, Gessel Development Schedules – Development Quotient, adapted measures from the Zurich Neuromotor Assessment test), achievement of developmental milestones (proxy-report questionnaire), coordination (proxy-report questionnaire), and fine motor coordination/fine motor development (proxy-report interview; Comprehensive Developmental Inventory for Infants and Toddlers).									
4	RCT^a^	Serious risk of bias^b^	No serious inconsistency	Serious indirectness^c^	No serious imprecision	None	705	The **PA interventions (planned passive cycling or structured/organized PA)** were *favourably* associated with improved motor development in **3 studies** [90–92]. The **PA intervention (PA recommendations from nurse)** was *not* associated with improved motor development in **1 study** [40].	LOW^d^
2	Clustered RCT^e^	Serious risk of bias^f^	No serious inconsistency	Serious indirectness^g^	No serious imprecision	None	1564	The **PA intervention (structured/organized PA)** was *favourably* associated with improved motor development (*total score and jumping individual score but not for running, hopping, catching, and kicking*) in **1 study** [33]. The **PA intervention (government-led PA program)** was *not* associated with motor development in **1 study** [41].	LOW^h^
6	Non-randomized intervention^i^	Serious risk of bias^j^	No serious inconsistency	No serious indirectness	No serious imprecision	None	946	The **PA interventions (free play and structured activities, structured/organized PA, dance, or swimming)** were *favourably* associated with improved motor development (*boys only and running speed between time 2 and 3 only in 1 study; one-leg balance only in 1 study*) in **6 studies** [36, 42, 93–96].	VERY LOW^k^
1	Longitudinal^l^	Serious risk of bias^m^	No serious inconsistency	No serious indirectness	No serious imprecision	None	197	**Prone position** was *favourably* associated with motor development (*higher prone duration and gross motor development only at age 6 mo but not at age 24 mo and prone-specific milestones only*) [97].	VERY LOW^n^
10	Cross-sectional^o^	Serious risk of bias^p^	No serious inconsistency	No serious indirectness	No serious imprecision	Exposure/outcome gradient^q^	1833	**TPA** was *favourably* associated with motor development (*correlations but not when comparing quartiles of fundamental movement skills in 1 study*) in **3 studies** [56, 69, 100], *unfavourably* associated with motor development (*running speed only in 1 study*) in **2 studies** [81, 101], and *not* associated with motor development in **1 study** [86]. **LPA** was *not* associated with motor development in **3 studies** [67, 86, 100]. **LPA 5-min bouts** were *not* associated with motor development in **1 study** [86]. **MVPA** was *favourably* associated with motor development (*total and locomotor [high* vs. *low only] but not for object control skills in 1 study*) in **3 studies** [67, 69, 100] and *not* associated with motor development in **1 study** [86]. **MVPA 5-min bouts** were *not* associated with motor development in **1 study** [86]. **VPA** was *favourably* associated with motor development (*total and locomotor* [*high* vs. *low only] but not for object control skills)* in **1 study** [67]. **Indoor PA** was *favourably* associated with motor development (*throwing at target only*) in **1 study** [81]**.** **Outdoor PA** was *not* associated with motor development in **1 study** [81]**.** **Prone position** was *favourably* associated with motor development (*gross motor development but not fine motor development in 1 study*) in **3 studies** [97–99].	VERY LOW^r^

LPA: light-intensity physical activity; MVPA: moderate- to vigorous-intensity physical activity; PA: physical activity; RCT: randomized controlled trial; TPA: total physical activity; VPA: vigorous-intensity physical activity

^a^Includes **4 RCTs** [40, 90–92]

^b^No intention-to-treat analysis; parent-child dyads were excluded if they did not carry out the management plan or if they became sick during the study; and the physical activity program was interrupted in 1 study [90]. Physical activity was not measured, so it is unknown if the intervention resulted in a significant change in physical activity in 3 studies [90–92]

^c^The intervention did not result in a significant change in physical activity in 1 study [40]

^d^Quality of evidence was downgraded from “high” to “low” because of serious risk of bias and serious indirectness

^e^Includes **2 clustered RCTs** [33, 41]

^f^Large amount of missing data primarily because mean attendance at child care was 48%, and it is unknown if the reason for poor attendance was related to the motor development in 1 study [41]

^g^The intervention did not result in a significant change in physical activity in 1 study [41]

^h^Quality of evidence was downgraded from “high” to “low” because of serious risk of bias and serious indirectness

^i^Includes **6 non-randomized interventions** [36, 42, 93–96]

^j^The outcome was measured post-intervention only in 2 studies [93, 96]. No control group in 1 study [42]. Physical activity was not measured so it is unknown if the intervention resulted in a significant change in physical activity in 6 studies [36, 42, 93–96]

^k^Quality of evidence was downgraded from “low” to “very low” because of serious risk of bias

^l^Includes **1 longitudinal study** [97]

^m^Psychometric properties unknown for the subjective physical activity measures

^n^Quality of evidence was downgraded from “low” to “very low” because of serious risk of bias

^o^Includes **10 cross-sectional studies** [56, 67, 69, 81, 86, 97–101]

^p^Convenience sample was used in 6 studies [56, 67, 69, 86, 99, 101]. Psychometric properties unknown for the subjective physical activity measure in 5 studies [56, 97–99, 101], and the outcome measure in 2 studies [69, 101]. Potential confounders were not adjusted for in 7 studies [67, 69, 81, 86, 98, 100, 101]. Large amount of missing motor development data in 1 study [67]

^q^A gradient for higher MVPA and VPA with better motor development was observed in 2 studies [67, 100]

^r^Quality of evidence was downgraded from “low” to “very low” because of serious risk of bias; because of this limitation, was not upgraded for an exposure/outcome gradient

In the two clustered RCTs, greater increases in total motor development and jumping were observed in the intervention group (structured/organized physical activity) compared to the control group (standard care) in one study; however, no such increases were seen in running, hopping, catching, or kicking [33]. In the second study, no significant difference was observed in motor skills between the intervention (government-led physical activity program) and control (standard care) groups [41]. However, physical activity was also not significantly different between groups [41]. The quality of evidence was downgraded from “high” to “low” because of a serious risk of bias and serious indirectness (see Table 2).

Among the six non-randomized interventions, significant increases in at least one measure of motor development were observed in the intervention group (free play and structured activities, structured/organized physical activity, dance program, or swimming) compared to the control group (usual care) in five studies [36, 93–96], and significant increases from baseline to follow-up in the 12-m run and standing long jump were observed in one study (structured/organized physical activity) [42]. However, for two of the interventions, more null than favourable effects were observed with the different motor development measures [94, 96]. One intervention had an infant sample at baseline [96]. The quality of evidence was downgraded from “low” to “very low” because of a serious risk of bias (see Table 2).

In the longitudinal study, higher duration of prone positioning at 4 months of age was favourably associated with the earlier achievement of several developmental milestones and gross motor development at 6 months but not at 24 months of age [97]. However, no significant differences were observed in fine motor development [97]. In separate analyses, no significant differences in motor development at 6 and 24 months of age were observed between infants who had, versus had not, experienced prone position at 4 months of age [97]. Apart from “crawled on abdomen”, significant differences for achievement of developmental milestones were also not observed between groups [97]. In further analyses comparing infants that preferred prone position at 6 months of age to those that did not, no significant differences were observed in gross and fine motor development at 24 months of age; however, the prone-preference group achieved several developmental milestones significantly earlier [97]. The quality of evidence was downgraded from “low” to “very low” because of a serious risk of bias (see Table 2).

Among the 10 cross-sectional studies, physical activity was favourably associated with at least one measure of motor development in seven studies [56, 67, 69, 97–100], unfavourably associated with motor development in one study [101], and not associated with motor development in one study [86]; mixed findings were observed in one study [81]. Three of the studies with favourable associations [97–99] and one study with unfavourable associations had infant samples [101]. One study with null findings had a toddler sample [86]. For the intensity or type of physical activity, at least one favourable association was observed between each of the following physical activity exposures and motor development: MVPA (3/4 studies), VPA (1/1 study), indoor physical activity (1/1 study), and prone position (3/3 studies). However, primarily null or mixed findings were observed between each of the following physical activity exposures and motor development: TPA, LPA, LPA bouts, MVPA bouts, and outdoor physical activity (see Table 1). The quality of evidence was downgraded from “low” to “very low” because of a serious risk of bias (see Table 2).

#### Psychosocial health

The association between physical activity and psychosocial health was examined in 11 studies (9 unique samples; see Table 3 and Table S3 in Additional file 2). Among the two RCTs, greater increases in psychosocial health were observed in the intervention groups (planned passive cycling or dance program) compared to the control groups (standard care) [90, 102]. One of the interventions had an infant sample [90]. The quality of evidence was downgraded from “high” to “moderate” because of a serious risk of bias (see Table 3).

**Table 3:** The relationship between physical activity and psychosocial health

# of studies	Design	Quality assessment					# of participants	Absolute effect	Quality
		Risk of bias	Inconsistency	Indirectness	Imprecision	Other			
Mean baseline age ranged from 18.3 weeks-57.61 months; where mean age was not reported, baseline age ranged from 12 months-5 years. Data were collected by RCT, clustered RCT longitudinal with up to 8- to 10-year follow-up, and cross-sectional study designs. Psychosocial health was assessed by social competence (proxy-report; Social Competence Behavior Evaluation: Preschool Education Questionnaire); internalizing behaviour problems (proxy-report; Social Competence Behavior Evaluation: Preschool Education Questionnaire); externalizing behaviour problems (proxy-report; Social Competence Behavior Evaluation: Preschool Education Questionnaire); quality of life (self-reported; Dartmouth Primary Care Cooperative Project charts); health-related quality of life (proxy-report; PedsQL 4.0); temper frequency (proxy-report interview); sociability, emotionality, and soothability (proxy-report; Child Temperament Questionnaire); conduct problems (proxy-report; Strengths and Difficulties Questionnaire); anxiety symptoms (proxy-report; Preschool Anxiety Scale – Revised); classroom peer acceptance (proxy-report; sociometric interviews); and personal-social behaviour (objectively measured; Gessell Development Schedules – Development Quotient).									
2	RCT^a^	Serious risk of bias^b^	No serious inconsistency	No serious indirectness	No serious imprecision	None	170	The **PA interventions (planned passive cycling or dance)** were *favourably* associated with improved psychosocial health in **2 studies** [90, 102].	MODERATE^c^
1	Clustered RCT^d^	Serious risk of bias^e^	No serious inconsistency	Very serious indirectness^f^	No serious imprecision	None	1467	The **PA intervention (government-led PA program)** was *not* associated with psychosocial health [41].	VERY LOW^g^
2	Longitudinal^h^	Serious risk of bias^i^	No serious inconsistency	No serious indirectness	No serious imprecision	Dose-response gradient^j^	9989	**TPA** was *favourably* associated with psychosocial health (*active* vs. *less active but not active* vs. *average*) in **1 study** [104] and *not* associated with psychosocial health in **1 study** [103]. **Sport participation** was *favourably* associated with psychosocial health (*high risk and recovery trajectories but not the rebound trajectory*) in **1 study** [103].	VERY LOW^k^
6	Cross-sectional^l^	Serious risk of bias^m^	Serious inconsistency^n^	No serious indirectness	No serious imprecision	None	5517	**TPA** was *unfavourably* associated with psychosocial health in **1 study** [101] and *not* associated with psychosocial health in **1 study** [109]. **MVPA** was *unfavourably* associated with psychosocial health in **1 study** [107] and *not* associated with psychosocial health in **1 study** [109]. **Bike riding** was *unfavourably* associated with psychosocial health (*for boys only on weekdays only in 1 study*) in **2 studies** [106, 107]. **Exercise play** was *favourably* associated with psychosocial health (*mixed gender [not non-mediated] and same gender but not other gender groups*) in **1 study** [105], *unfavourably* associated with psychosocial health (*boys only, weekend only, and only for > 2 and ≤ 24 h group)* in **1 study** [106], and *not* associated with psychosocial health in **1 study** [107]. **Routh-and-tumble play** was *not* associated with psychosocial health in **2 studies** [105, 108]. **Walking** was *not* associated with psychosocial health in **2 studies** [106, 107].	VERY LOW^o^

MVPA: moderate- to vigorous-intensity physical activity; PA: physical activity; RCT: randomized controlled trial; TPA: total physical activity

^a^Includes **2 RCTs** [90, 102]

^b^No intention-to-treat analysis; parent-child dyads were excluded if they did not carry out the management plan or if they became sick during the study and the physical activity program was interrupted in 1 study [90]. Physical activity was not measured, so it is unknown if the intervention significantly changed physical activity in 2 studies [90, 102]

^c^Quality of evidence was downgraded from “high” to “moderate” because of serious risk of bias

^d^Includes **1 clustered RCT** [41]

^e^Large amount of missing data primarily because mean attendance at child care was 48%, and it is unknown if hte reason for poor attendance was related to psychosocial health

^f^The intervention did not result in a significant change in physical activity

^g^Quality of evidence was downgraded from “high” to “very low” because of serious risk of bias and very serious indirectness

^h^Includes **2 longitudinal studies** [103, 104]

^i^No psychometric properties reported for the subjective physical activity measures in 2 studies [103, 104]

^j^A significant trend was observed for poor quality of life when moving from the active to less active groups in 1 study [104]

^k^Quality of evidence was downgraded from “low” to “very low” because of serious risk of bias; because of this limitation, was not upgraded for a dose-response gradient

^l^Includes **6 cross-sectional studies** [101, 105–109]

^m^Convenience sample was used in 5 studies [101, 105–108]. Physical activity was measured only during child care in 1 study [109]. Potential confounders were not adjusted for in 3 adjusted studies [101, 107, 109]. No psychometric properties reported for the subjective physical activity measures in 1 study [101]. No psychometric properties reported for the outcome measure in 2 studies [101, 105]. Large amount of missing data in 1 study [106]

^n^Favourable and unfavourable associations between physical activity and psychosocial health observed across studies

^o^Quality of evidence was downgraded from “low” to “very low” because of serious risk of bias and serious inconsistency

In the clustered RCT, no significant differences in quality of life were observed between the intervention (government-led physical activity program) and control (standard care) groups [41]. Physical activity was also not significantly different between groups [41]. The quality of evidence was downgraded from “high” to “very low” because of a serious risk of bias and very serious indirectness (see Table 3).

Among the two longitudinal studies, sport participation was favourably associated with psychosocial health in one study [103], and TPA was favourably associated with psychosocial health in one study [104] but not the other [103]. The quality of evidence was downgraded from “low” to “very low” because of a serious risk of bias (see Table 3).

Among the six cross-sectional studies, physical activity was favourably associated with at least one measure of psychosocial health in one study [105], unfavourably associated with at least one measure of psychosocial health in three studies [101, 106, 107], and not associated with psychosocial health in two studies [108, 109]. However, primarily null associations were observed in all studies. One study with unfavourable associations had an infant sample [101]. In regard to intensity or type of physical activity, at least one favourable association was observed between MVPA and psychosocial health (1/2 studies), and at least one unfavourable association was observed between bike riding and psychosocial health (2/2 studies). However, primarily null or mixed findings were observed between each of the following physical activity exposures and psychosocial health: TPA, exercise play, rough-and-tumble play, and walking (see Table 3). The quality of evidence was downgraded from “low” to “very low” because of a serious risk of bias and serious inconsistency (see Table 3).

#### Cognitive development

The association between physical activity and cognitive development was examined in 13 studies (13 unique samples; see Table 4 and Table S4 in Additional file 2). Among the two RCTs, significant increases in cognitive development were observed in the intervention groups (planned passive cycling or structured/organized physical activity) compared to the control groups (standard care) [90, 91]. One intervention involved an infant sample [90]. The quality of evidence was downgraded from “high” to “moderate” because of a serious risk of bias (Table 4).

**Table 4:** The relationship between physical activity and cognitive development

# of studies	Design	Quality assessment					# of participants	Absolute effect	Quality
		Risk of bias	Inconsistency	Indirectness	Imprecision	Other			
Mean baseline age ranged from 18.3 weeks-4.94 years; where mean age was not reported, baseline age ranged from 12 months-5 years. Data were collected by RCT, clustered RCT, non-randomized intervention, cross-over trial, and cross-sectional study designs. Cognitive development was assessed by psychomotor skills (objectively measured), time on task (direct observation), early literacy and language skills (objectively measured), creativity (direct observation; Thinking Creatively in Action and Movement test), attention (direct observation), attention span (proxy-report interview; proxy-report Child Temperament Questionnaire), literacy skills (self-report; Woodcock Johnson, Peabody Picture Vocabulary Test), math skills (self-report; Woodcock Johnson Applied Problems subscale), language development (objectively measured; Gessell Developmental Schedules – Development Quotient), free and cued word recall (objectively measured), cognitive function (objectively measured; Herbst Test), and sustained attention and response inhibition (objectively measured; Picture Deletion Task for Preschoolers).									
2	RCT^a^	Serious risk of bias^b^	No serious inconsistency	No serious indirectness	No serious imprecision	None	454	The **PA interventions (planned passive cycling or structured/organized PA)** were *favourably* associated with improved cognitive development in **2 studies** [90, 91].	MODERATE^c^
1	Clustered RCT^d^	No serious risk of bias	No serious inconsistency	No serious indirectness	No serious imprecision	None	125	The **PA intervention (physical exercises to enact meanings of words)** was *favourably* associated with improved cognitive development [110]. The **PA intervention (physical exercises unrelated to words)** was *favourably* associated with improved cognitive development (*cued recall of words but not free recall of words*) [110].	HIGH
4	Non-randomized intervention^e^	Serious risk of bias^f^	No serious inconsistency	No serious indirectness	No serious imprecision	None	460	The **PA interventions (structured/organized PA, free play and structured PA, or academic MVPA lessons)** were *favourably* associated with improved cognitive development (*only in intervention sites that actively participated in the intervention in 1 study; for alliteration in 2/2 studies; and for rhyming and picture naming in 1/2 studies*) in **4 studies** [93, 111–113].	VERY LOW^g^
3	Cross-over trial^h^	Serious risk of bias^i^	No serious inconsistency	No serious indirectness	No serious imprecision	None	182	The **PA condition (structured/organized PA or MVPA breaks)** was *favourably* associated with improved cognitive development (*sustained attention but not response inhibition in 1 study*) in **2 studies** [114, 115]. **Recess conditions** were *favourably* associated with cognitive development in **1 study** [116].	VERY LOW^j^
3	Cross-sectional^k^	Serious risk of bias^l^	No serious inconsistency	No serious indirectness	No serious imprecision	None	3138	**TPA** was *unfavourably* associated with cognitive development in **1 study** [101] and *not* associated with cognitive development in **1 study** [109]. **MVPA** was *not* associated with cognitive development in **1 study** [109]. **Outdoor PA** (*at child care*) was *not* associated with cognitive development in **1 study** [58].	VERY LOW^m^

MVPA: moderate- to vigorous-intensity physical activity; PA: physical activity; RCT: randomized controlled trial; TPA: total physical activity

^a^Includes **2 RCTs** [90, 91]

^b^No intention-to-treat analysis; parent-child dyads were excluded if they did not carry out the management plan or if they became sick during the study and the physical activity program was interrupted in 1 study [90]. Physical activity was not measured, so it is unknown if the intervention significantly changed physical activity in 2 studies [90, 91]

^c^Quality of evidence was downgraded from “high” to “moderate” because of serious risk of bias

^d^Includes **1 clustered RCT** [110]

^e^Includes **4 non-randomized interventions** [93, 111–113]

^f^Physical activity was not measured, so it is unknown if the intervention significantly changed physical activity in in 2 studies [93, 113]

^g^Quality of evidence was downgraded from “low” to “very low” because of serious risk of bias

^h^Includes **3 cross-over trials** [114–116]

^i^Condition was not randomly assigned in 1 study [116]. Physical activity was not measured, so it is unknown if there were significant differences in physical activity between conditions in 2 studies [114, 116]. Unclear what conditions had significant differences in the outcome measure in 1 study [116]

^j^Quality of evidence was downgraded from “low” to “very low” because of serious risk of bias

^k^Includes **3 cross-sectional studies** [58, 101, 109]

^l^Convenience sample was used in 1 study [101]. Physical activity was measured only during child care in 2 studies [58, 109]. No potential confounders were adjusted for in 2 adjusted studies [101, 109]. No psychometric properties reported for the subjective physical activity measure or the outcome measure in 1 study [101]

^m^Quality of evidence was downgraded from “low” to “very low” because of serious risk of bias

For the clustered RCT, significant increases in free and/or cued recalls of previously learned Italian words were observed in the physical activity intervention groups (physical activity to enact meaning of words and physical activity unrelated to words) compared to the control groups (no physical activity) [110]. The quality of evidence remained at “high” (Table 4).

Among the four non-randomized interventions, a significant increase in at least one measure of cognitive development was observed in the intervention groups that participated in the intervention (academic lessons, free play, and structured activities) compared to the control groups (standard care) in three studies [93, 111, 112], and significant increases in children’s creativity at follow-up compared to baseline were reported in one study [113]. The quality of evidence was downgraded from “low” to “very low” because of a serious risk of bias (Table 4).

Among the three cross-over trials, at least one measure of cognitive development was significantly higher in the physical activity condition (MVPA breaks, structured/organized physical activity) compared to the control condition (typical instruction, sedentary session) in two studies [114, 115], and attention was significantly higher after 10-, 20-, and 30-min outdoor recess conditions in one study [116]. The quality of evidence was downgraded from “low” to “very low” because of a serious risk of bias (Table 4).

Among the three cross-sectional studies, physical activity was unfavourably associated with cognitive development in one study [101] and not associated with cognitive development in two studies [58, 109]. The study with unfavourable associations had a sample of infants [101]. In regard to intensity or type of physical activity, at least one favourable association was observed between TPA and cognitive development (1/2 studies). However, MVPA and outdoor physical activity were not associated with cognitive development (see Table 4). The quality of evidence was downgraded from “low” to “very low” because of a serious risk of bias (see Table 4).

#### Fitness

The association between physical activity and fitness was examined in three studies (three unique samples; see Table 5 and Table S5 in Additional file 2). In the longitudinal study, TPA was favourably associated with cardiorespiratory fitness [43]. The quality of evidence was downgraded from “low” to “very low” because of a serious risk of bias (see Table 5).

**Table 5:** The relationship between physical activity and fitness

# of studies	Design	Quality assessment					# of participants	Absolute effect	Quality
		Risk of bias	Inconsistency	Indirectness	Imprecision	Other			
Mean baseline age ranged from 4.04-4.48 years. One study reported the sample was of preschool age but did not provide a mean or range. Data were collected by longitudinal with 1-year follow-up and cross-sectional study designs. Fitness was assessed as cardiorespiratory fitness (treadmill test, 20-m shuttle run from the PREFIT fitness test battery), muscular fitness including handgrip strength and standing long jump (PREFIT fitness test battery), speed-agility (4 × 10 shuttle run from the PREFIT fitness test battery), and physical working capacity (Ruffier’s test using Ruffier–Dickson index). All outcomes were objectively measured.									
1	Longitudinal^a^	Serious risk of bias^b^	No serious inconsistency	No serious indirectness	No serious imprecision	None	123	**TPA** was *favourably* associated with **cardiorespiratory fitness** [43].	VERY LOW^c^
2	Cross-sectional^d^	Serious risk of bias^e^	No serious inconsistency	No serious indirectness	No serious imprecision	Exposure/outcome gradient^f^	594	***Cardiorespiratory fitness*** **TPA** was *favourably* associated with fitness (*only for 95th, 90th, 75th but not 50th and 25th percentiles of vector magnitude in 1 study*) in **2 studies** [55, 117]. **LPA** was *not* associated with fitness in **1 study** [55]. **MPA** was *not* associated with fitness in **1 study** [55]. **MVPA** was *favourably* associated with fitness in **1 study** [55]. **VPA** was *favourably* associated with fitness in **1 study** [55]. ***Other fitness measures*** **TPA** was *favourably* associated with muscular fitness and speed-agility (*only for 95th, 90th, 75th but not 50th and 25th percentiles of vector magnitude and not for standing long jump at the 75th percentile*) in **1 study** [55]. **LPA** was *not* associated with muscular fitness and speed-agility in **1 study** [55]. **MPA** was *not* associated with muscular fitness and speed-agility in **1 study** [55]. **MVPA** was *favourably* associated with muscular fitness (*standing long jump but not handgrip strength)* and speed-agility in **1 study** [55]. **VPA** was *favourably* associated with muscular fitness and speed-agility in **1 study** [55].	VERY LOW^g^

LPA: light-intensity physical activity; MPA: moderate-intensity physical activity; MVPA: moderate- to vigorous-intensity physical activity; TPA: total physical activity; VPA: vigorous-intensity physical activity

^a^Includes **1 longitudinal study** [43]

^b^The findings that were reported did not adjust for any potential confounders

^c^Quality of evidence was downgraded from “low” to “very low” because of serious risk of bias

^d^Includes **2 cross-sectional studies** [55, 117]

^e^No potential confounders were adjusted for; a convenience sample was used and it is unclear if the fitness measure is suitable for this age group in 1 study [117]. Potentially inappropriate statistical analysis: other movement behaviours were mutually adjusted for in the fully adjusted models in 1 study [55]

^f^A gradient for higher TPA, MVPA, VPA with higher fitness was observed in 1 study [55]

^g^Quality of evidence was downgraded from “low” to “very low” because of serious risk of bias; because of this limitation, was not upgraded for an exposure/outcome gradient

Among the two cross-sectional studies, physical activity was favourably associated with at least one measure of fitness in both studies [55, 117]. As for physical activity intensity or type, at least one favourable association was observed between each of the following physical activity exposures and cardiorespiratory fitness: TPA (2/2 studies), MVPA (1/1 study), and VPA (1/1 study). Similarly, at least one favourable association was observed between each of the following physical activity exposures and muscular fitness and speed-agility: TPA (1/1 study), MVPA (1/1 study), and VPA (1/1 study). However, null LPA and MPA were not associated with cardiorespiratory fitness, muscular fitness or speed-agility (See Table 5). The quality of evidence was downgraded from “low” to “very low” because of a serious risk of bias (see Table 5).

#### Bone and skeletal health

The association between physical activity and bone and skeletal health was examined in seven studies (seven unique samples; see Table 6 and Table S6 in Additional file 2). For the RCT, total bone mineral content in a baseline sample of infants was not significantly different between the intervention (structured/organized physical activity) and control (fine motor activity) groups [118]. However, physical activity was also not significantly different between groups. The quality of evidence was downgraded from “high” to “low” because of very serious indirectness (see Table 6).

**Table 6:** The relationship between physical activity and bone and skeletal health

# of studies	Design	Quality assessment					# of participants	Absolute effect	Quality
		Risk of bias	Inconsistency	Indirectness	Imprecision	Other			
Mean baseline age ranged from 9.27-57.12 months. One study reported the baseline age as 6 months but a mean was not given. Data were collected by RCT and cross-sectional study designs. Several bone and skeletal health measures were assessed by X-ray absorptiometry including: total bone mineral content, bone mineral density of the lumbar spine (L2-L4), total body bone area, periosteal circumference of tibia, endosteal circumference of tibia, cortical bone area of tibia, hip bone area, hip bone mineral content, areal bone mineral density, and estimated volumetric bone mineral density. Bone and skeletal health was also assessed by vitamin D (25-(OH)- vitamin D3 measured in serum), vitamin D (25-(OH)- vitamin D3 parathyroid hormone in non-fasting venous blood samples), and bone stiffness (quantitative ultrasound). All outcomes were objectively measured.									
1	RCT^a^	No risk of bias	No serious inconsistency	Very serious indirectness^b^	No serious imprecision	None	422	The **PA intervention (structured/organized PA)** was *not* associated with improved bone mineral content [118].	LOW^c^
6	Cross-sectional^d^	Serious risk of bias^e^	No serious inconsistency	No serious indirectness	No serious imprecision	Exposure/outcome gradient^f^	14,774	**TPA** was *favourably* associated with bone and skeletal health in **2 studies** [119, 123] and *not* associated with bone and skeletal health in **1 study** [124]. **LPA** was *not* associated with bone and skeletal health in **1 study** [123]. **MPA** was *favourably* associated with bone and skeletal health in **1 study** [123] and *not* associated with bone and skeletal health in **1 study** [124]. **MVPA** was *favourably* associated with bone and skeletal health in **2 studies** [122, 123] and *not* associated with bone and skeletal health in **1 study** [124]. **VPA** was *not* associated with bone and skeletal health in **2 studies** [123, 124]. **Leisure time physical activity** was *favourably* associated with bone and skeletal health in **1 study** [123]. **Outdoor activity** was *favourably* associated with bone and skeletal health in **3 studies** [119–121]. **Weight-bearing activity** was *favourably* associated with bone and skeletal health in **1 study** [123].	VERY LOW^g^

LPA: light-intensity physical activity; MPA: moderate-intensity physical activity; MVPA: moderate- to vigorous-intensity activity; PA: physical activity; RCT: randomized controlled trial; TPA: total physical activity; VPA: vigorous-intensity physical activity

^a^Includes **1 RCT** [118]

^b^The intervention did not significantly change physical activity

^c^Quality of evidence was downgraded from “high” to “low” because of very serious indirectness

^d^Includes **6 cross-sectional studies** [119–124]

^e^Potential confounders were not adjusted for in 2 studies [120, 121]. Potentially inappropriate statistical analysis: other movement behaviours were mutually adjusted for in the fully adjusted models in 1 study [123]. No psychometric properties were reported for the subjective physical activity measure in 4 studies [119–121, 123]. A convenience sample was used in 2 studies [120, 124]

^f^A gradient for higher TPA, MPA, MVPA, leisure time physical activity, outdoor activity, and weight-bearing physical activity with better bone and skeletal health was observed in 2 studies [119, 123]

^g^Quality of evidence was downgraded from “low” to “very low” because of serious risk of bias; because of this limitation, was not upgraded for an exposure/outcome gradient

Among the six cross-sectional studies, favourable associations were reported between physical activity and at least one measure of bone and skeletal health in five studies [119–123], and null associations were reported in one study [124]. One study with favourable associations had a sample of infants [119]. In regard to intensity or type of physical activity, at least one favourable association was observed between each of the following physical activity exposures and bone and skeletal health: TPA (2/3 studies), MPA (1/2 studies), MVPA (2/3 studies), VPA (2/2 studies), leisure time physical activity (1/1 study), outdoor physical activity (3/3 studies), and weight-bearing physical activity (1/1 study). Conversely, LPA was not associated with bone and skeletal health (see Table 6). The quality of evidence was downgraded from “low” to “very low” because of a serious risk of bias (see Table 6).

#### Cardiometabolic health

The association between physical activity and cardiometabolic health was examined in nine studies (eight unique samples; see Table 7 and Table S7 in Additional file 2). In the non-randomized intervention, children in the intervention group (structured/organized physical activity) had significantly lower diastolic blood pressure than the controls (standard care) [125]. The quality of evidence was downgraded from “low” to “very low” because of a serious risk of bias (see Table 7).

**Table 7:** The relationship between physical activity and cardiometabolic health

# of studies	Design	Quality assessment					# of participants	Absolute effect	Quality
		Risk of bias	Inconsistency	Indirectness	Imprecision	Other			
Mean baseline age ranged from 3-4.9 years. One study reported only that the children were preschool age. Data were collected by non-randomized intervention, longitudinal with up to 2 years follow-up, and cross-sectional study designs. Cardiometabolic health was assessed by mean arterial pressure, DBP, SBP, total cholesterol, total serum cholesterol, HDL, triglycerides, HDL_2_, LDL, LDL/HDL, total serum cholesterol/HDL, HDL/total triglycerides, and clustered cardiovascular risk score (SBP, triglycerides, total cholesterol/HDL, HOMA-IR, sum of two skinfolds). All outcomes were objectively measured.									
1	Non-randomized intervention^a^	Serious risk of bias^b^	No serious inconsistency	No serious indirectness	No serious imprecision	None	264	***BP*** The **PA intervention (structured/organized PA)** was *favourably* associated with DBP during rest and activity [125].	VERY LOW^c^
2	Longitudinal^d^	Serious risk of bias^e^	No serious inconsistency	No serious indirectness	No serious imprecision	None	291	***BP*** **Aerobic PA** was *favourably* associated with BP (*SBP but not DBP, boys only, 2-year follow-up but not 1-year follow-up*) in **1 study** [126]. **Leisure PA** was *unfavourably* associated with BP (*DBP but not SBP, boys only, 1-year follow-up but not 2-year follow-up*) in **1 study** [126]. **Structured PA** was *not* associated with BP (*SBP or DBP*) in **1 study** [126]. ***Cholesterol*** **TPA** was *not* associated with cholesterol (*total serum cholesterol, HDL, HDL*_*2*_ *, LDL, LDL/HDL, or total serum cholesterol/HDL*) in **1 study** [43]. ***Triglycerides*** **TPA** was *not* associated with triglycerides in **1 study** [43].	VERY LOW^f^
6	Cross-sectional^g^	Serious risk of bias^h^	Serious inconsistency^i^	No serious indirectness	No serious imprecision	Exposure/outcome gradient^j^	1882	***Clustered risk score*** **TPA** was *favourably* associated with clustered risk score (*boys only, Quartile 1* vs. *Quartile 5 only*) in **1 study** [127]. **MPA** was *not* associated with clustered risk score in **1 study** [127]. **MVPA** was *not* associated with clustered risk score in **1 study** [127]. **VPA** was *favourably* associated with clustered risk score (*boys only, Quartile 2* vs. *Quartile 5 only*) in **1 study** [127]. ***BP*** **TPA** was *unfavourably* associated with BP (*SBP and DBP*) in **1 study** [117] and *not* associated with BP (*SBP, DBP, or mean arterial pressure*) in **3 studies** [66, 72, 81]. **Aerobic PA** was *not* associated with BP (*SBP or DBP*) in **1 study** [126]. **Indoor PA** was *not* associated with BP (*SBP or DBP*) in **1 study** [81]. **Leisure PA** was *not* associated with BP (*SBP or DBP*) in **1 study** [126]. **Outdoor PA** was *not* associated with BP (*SBP or DBP*) in **1 study** [81]. **Structured PA** was *not* associated with BP (*SBP or DBP*) in **1 study** [126]. ***Cholesterol*** **TPA** was *favourably* associated with cholesterol (*total cholesterol but not HDL*) in **1 study** [81] and *not* associated with cholesterol (*total cholesterol, HDL, or HDL/total cholesterol)* in **1 study** [72]. **Indoor PA** was *not* associated with cholesterol (*total cholesterol or HDL*) in **1 study** [81]. **Outdoor PA** was *unfavourably* associated with cholesterol (*HDL but not total cholesterol*) in **1 study** [81]. ***Triglycerides*** **TPA** was *not* associated with cholesterol (*total cholesterol, HDL, or HDL/total cholesterol*) in **1 study** [72].	VERY LOW^k^

BP: blood pressure; DBP: diastolic blood pressure; HDL: high-density lipoprotein cholesterol; HOMA-IR: homeostatic model assessment – insulin resistance; LDL: low-density lipoprotein cholesterol; MPA: moderate-intensity physical activity; MVPA: moderate- to vigorous-intensity physical activity; PA: physical activity; SBP: systolic blood pressure; TPA: total physical activity; VPA: vigorous intensity physical activity

^a^Includes **1 non-randomized intervention** [125]

^b^No intention-to-treat analysis; results are based on children who were measured at all 3 time points. Physical activity was not measured, so it is unknown if the intervention significantly changed physical activity

^c^Quality of evidence was downgraded from “low” to “very low” because of serious risk of bias

^d^Includes **2 longitudinal studies** [43, 126]

^e^Potential confounders were not adjusted for in 1 study [43]. No psychometric properties were reported for the subjective physical activity measure in 1 study [126]

^f^Quality of evidence was downgraded from “low” to “very low” because of serious risk of bias

^g^Includes **6 cross-sectional studies** [66, 72, 81, 117, 126, 127]

^h^No potential confounders were adjusted for in 5 studies [66, 72, 81, 117, 127]. Convenience sample in 1 study [117]. No psychometric properties were reported for the subjective physical activity measure in 1 study [126]

^i^Favourable and unfavourable associations between physical activity and cardiometabolic health observed across studies

^j^A gradient for higher TPA with worse total cholesterol was observed in 1 study [81]

^k^Quality of evidence was downgraded from “low” to “very low” because of serious risk of bias and serious inconsistency; because of this limitation, was not upgraded for an exposure/outcome gradient

Among the two longitudinal studies, physical activity was not associated with any measure of blood pressure, cholesterol, or triglycerides in one study [43], and mixed findings with blood pressure were observed in the other study, though primarily null associations were observed [126]. In regard to intensity or type of physical activity, at least one favourable association was observed between aerobic physical activity and blood pressure (1/1 study), and at least one unfavourable association was observed between leisure physical activity and blood pressure (1/1 study). Structured physical activity was not associated with blood pressure [126]. Similarly, TPA was not associated with cholesterol or triglycerides (See Table 7). The quality of evidence was downgraded from “low” to “very low” because of a serious risk of bias (see Table 7).

Among the six cross-sectional studies, physical activity was favourably associated with at least one measure of cardiometabolic health in one study [127] and unfavourably associated with cardiometabolic health in one study [117]; null associations were found in three studies [66, 72, 126], and mixed findings were found in one study [81]. In the study where some favourable associations were observed, primarily null associations were observed [127]. In regard to intensity or type of physical activity, at least one favourable association was observed between each of the following physical activity exposures and a clustered risk score: TPA (1/1 study) and VPA (1/1 study). However, MPA and MVPA were not associated with a clustered risk score. The following types of physical activity were not associated with blood pressure: TPA, aerobic physical activity, indoor physical activity, outdoor physical activity, leisure physical activity, and structured physical activity. At least one favourable association was observed between TPA and cholesterol (1/2 studies), and at least one unfavourable association was observed between outdoor physical activity and cholesterol (1/1 study). However, indoor physical activity was not associated with cholesterol. Similarly, TPA was not associated with triglycerides (see Table 7). The quality of evidence for the cross-sectional studies was downgraded from “low” to “very low” because of a serious risk of bias and serious inconsistency (see Table 7).

#### Risks/harm

The association between physical activity and risks/harm was examined in four studies (four unique samples; see Table 8 and Table S8 in Additional file 2). In the case cross-over study, a high activity level, compared to a low activity level, was unfavourably associated with injury risk among toddlers, but activity level was not associated with injury severity [128]. The quality of evidence remained at “low” (see Table 8).

**Table 8:** The relationship between physical activity and risks/harm

# of studies	Design	Quality assessment					# of participants	Absolute effect	Quality
		Risk of bias	Inconsistency	Indirectness	Imprecision	Other			
Mean baseline age ranged from 7.4 weeks-24 months; where mean age was not reported, baseline age ranged from 2 months-4.5 years. Data were collected by case cross-over and longitudinal with 4.5-6.5 years follow-up, case control, and cross-sectional study designs. Risks/harm was assessed as injury risk (proxy-report; Participant Event Monitoring method), injury severity (proxy-report; minor injury severity scale), fracture incidence (proxy-report), and plagiocephaly (objectively measured).									
1	Case cross-over^a^	Serious risk of bias^b^	No serious inconsistency	No serious indirectness	No serious imprecision	None	170	**TPA** was *unfavourably* associated with injury risk but was *not* associated with injury severity [128].	LOW^c^
1	Longitudinal^d^	Serious risk of bias^e^	No serious inconsistency	Serious indirectness^f^	No serious imprecision	Dose-response evidence^g^	2692	**Outdoor time** was *favourably* associated with fracture incidence in the winter but *unfavourably* associated with fracture incidence in the summer [129].	VERY LOW^h^
1	Case-control^i^	Serious risk of bias^j^	No serious inconsistency	No serious indirectness	No serious imprecision	None	194	**TPA** was *favourably* associated with plagiocephaly (*at present but not at 6 weeks of age*) [130]. **Prone position** was *favourably* associated with plagiocephaly (*for ≥ 5 min/day but not whether it was provided or not*) at 6 weeks of age [130].	VERY LOW^k^
1	Cross-sectional^l^	Serious risk of bias^m^	No serious inconsistency	No serious indirectness	No serious imprecision	None	380	**Prone position** was *not* associated with plagiocephaly [131].	VERY LOW^n^

min: minutes; TPA: total physical activity

^a^Includes **1 case cross-over study** [128]

^b^Convenience sample

^c^Quality of evidence remained at “low”

^d^Includes **1 longitudinal study** [129]

^e^No psychometric properties were reported for outdoor time and fracture incidence, and there was a large unexplained loss to follow-up

^f^Outdoor time was the measure of physical activity

^g^Dose-response evidence was observed for higher outdoor time with lower fracture incidence

^h^Quality of evidence was downgraded from “low” to “very low” because of serious risk of bias and serious indirectness; because of these limitations, was not upgraded for dose-response evidence

^i^Includes **1 case-control** study [130]

^j^No psychometric properties were reported for the subjective physical activity measures

^k^Quality of evidence was downgraded from “low” to “very low” because of serious risk of bias

^l^Includes **1 cross-sectional study** [131]

^m^Convenience sample and no psychometric properties were reported for the subjective physical activity measure

^n^Quality of evidence was downgraded from “low” to “very low” because of serious risk of bias

In the longitudinal study, findings differed based on the season, with more outdoor time in the summer associated with an increased likelihood of reporting a fracture, and more outdoor time in the winter associated with a decreased likelihood of reporting a fracture [129]. The quality of evidence was downgraded from “low” to “very low” because of a serious risk of bias and serious indirectness (see Table 8).

In the case-control study, cases (those with plagiocephaly) had an increased likelihood of being in the very inactive/inactive/average group compared to the active/very active group [130]. Cases were also more likely to participate in a lower duration of prone position per day. The quality of evidence was downgraded from “low” to “very low” because of a serious risk of bias (see Table 8).

In the cross-sectional study, no associations were observed between first age of tummy time or tummy time duration and plagiocephaly [131]. Infants with a lower frequency of tummy time were more likely to have plagiocephaly in unadjusted models but not in adjusted models. The quality of evidence was downgraded from “low” to “very low” because of a serious risk of bias (see Table 8).

### Frequency and duration

The impact of different frequencies or durations of physical activity on health indicators could be compared only across studies (i.e., within-study comparisons were not possible), as most studies dichotomized physical activity frequency and duration or had only a single- or two-arm physical activity intervention.

In 19 experimental studies, times per day of intervention delivery were reported [34, 35, 42, 90–95, 102, 111–116, 118, 125]. Regardless of frequency, the intervention had favourable impacts on at least one health indicator in the majority of studies (1 time/day, 14/15 studies; 2 times/day, 2/3 studies; 4 times/day, 1/1 studies). In 19 experimental studies, times per week of intervention delivery were reported or could be calculated [33, 35, 36, 42, 90–95, 102, 111–116, 118, 125]. Regardless of frequency, favourable impacts on at least one health indicator were observed in the majority of studies (1 time/week, 3/3 studies; 2 times/week, 6/6 studies; 3-4 times/week, 6/6 studies; 5 times/week, 0/1 study; 6 times/week, 0/1 study; 10 times/week, 2/2 studies; 24 times/week, 1/1 study).

Only five observational studies examined the association between frequency of physical activity and a health indicator [56, 71, 120, 121, 131]. In one study, participants who engaged in TPA <5 times per week were significantly more likely to have a motor difficulty compared to participants who engaged in TPA >5 times per week [56]. It is important to note that this study measured TPA with a questionnaire; therefore, LPA was likely underestimated or not captured [11]. In one bone and skeletal health study, vitamin D levels were significantly lower in children who participated in 0, 1-5, 6-10, 11-15, or 16-20 times per month, compared to children who participated in outdoor physical activity 26-31 times per month [120]. However, in a second study, only children with no outdoor physical activity were significantly more likely to have lower vitamin D status compared to children who participated in outdoor physical activity 26-31 times per month [121]. In another study, infants who participated in the prone position <3 times per day were significantly more likely to have plagiocephaly in the unadjusted but not in the adjusted models [131]. Similarly, the proportion of girls or boys participating in active play <7 times per week was not significantly different between non-obese and obese groups in one study [71].

In 17 experimental studies, duration of intervention delivery per day was reported [34–36, 42, 90, 91, 93–95, 111–116, 118, 125]. Regardless of duration, favourable impacts on at least one health indicator were observed in the majority of studies (10-15 min/day, 3/3 studies; 15-20 min/day, 0/1 study; 20-40 min/day, 7/8 studies; 45-60 min/day, 8/8 studies; note one study included three different durations that all had favourable impacts but statistical comparisons between groups were not made [116]). In 18 experimental studies, total duration of intervention delivery per week was reported or could be calculated [35, 36, 42, 90, 91, 93–96, 102, 111–116, 118, 125]. Regardless of duration, favourable impacts on at least one health indicator were observed in the majority of studies (10-30 min/week, 5/5 studies; 45 min/week, 2/2 studies; 70-100 min/week, 5/6 studies; 105-240 min/week, 5/7 studies; 300 min/week, 1/1 study; note one study included three different durations that all had favourable impacts but statistical comparisons between groups were not made [116]).

As for duration, 17 observational studies examined the association between duration of physical activity and a health indicator [45, 47, 53, 56, 58, 63, 73, 74, 78, 79, 98, 99, 106, 120, 129–131]. In infants, ≥5 h per day of unrestricted moving time was associated with favourable changes in one measure of adiposity in one study [45]. Additionally, ≥30 min of prone position per day in one study [98] and ≥60 min of prone position per day in another study [99] were associated with more favourable motor development scores or an increased likelihood of achieving motor development milestones at an earlier age. While ≥5 min of prone position per day was favourably associated with plagiocephaly in one study [130], >5 min of prone position per day was not associated with plagiocephaly in another study [131]. For toddlers and/or preschoolers, one study found that those who participated in TPA for ≥7 h per week were significantly less likely to be overweight or obese [63], and a second study found that those who participated in TPA for <840 min per week (<14 h per week) were significantly more likely to have a motor difficulty [56]. In one study, unfavourable associations between ≥2 h of exercise per day and psychosocial health were observed in boys but not in girls [106].

Six studies examined the association between duration of outdoor physical activity and a health indicator [53, 58, 78, 79, 120, 129]; the findings were mixed. Specifically, >30 min of outdoor physical activity per day was favourably associated with adiposity [58]; ≥1 h per day was favourably associated with bone and skeletal health [120]; and ≥28 h per week were unfavourably associated with risks/harm [129]. Null associations were observed between outdoor physical activity and adiposity in three studies [53, 78, 79], using a > 1 h per day, ≥2 h per day, and ≥7 h per week cut-points. Null associations were also observed in the remaining three studies that examined duration of physical activity [47, 73, 74]. For instance, the following durations were not associated with adiposity: ≥2 h of active play per weekday and ≥4 h per weekend day in one study [73], <60 min of VPA per day outside of kindergarten in one study [74], and ≤51.43 min per day of physical activity at home and ≤34.59 min per day of structured physical activity in one study [47].

## Discussion

In this systematic review, evidence from 96 studies and 71,291 unique participants was synthesized to examine the relationships between objectively and subjectively measured physical activity and health indicators in the early years. For experimental studies, physical activity was consistently (>60% of studies) associated with improved motor development, cognitive development, psychosocial health, and cardiometabolic health. For observational studies, physical activity was consistently associated with favourable motor development, fitness, and bone and skeletal health. However, physical activity was not consistently associated with adiposity or risks/harm across study designs, and significant differences between intervention and control groups in BMI were not observed in the meta-analysis. Although some high-quality evidence was included, the vast majority of evidence was of “low” to “very low” quality. A high-level summary is provided in Table 9.

**Table 9:** High-level summary of findings by health indicator across all, experimental and observational study designs

Health indicator	# of studies	Quality of evidence	Summary of findings^a^		
			Experimental study designs	Observational study designs	All designs
Critical					
Adiposity	57	Very low to low	Favourable: 2 studies	Favourable: 16 studies	Favourable: 18 studies
			Null: 5 studies	Null: 25 studies	Null: 30 studies
			Unfavourable: 0 studies	Unfavourable: 4 studies	Unfavourable: 4 studies
			Mixed: 0 studies	Mixed: 5 studies	Mixed: 5 studies
Motor development	23	Very low to low	**Favourable: 10 studies**	**Favourable: 8 studies**	**Favourable: 18 studies**
			Null: 2 studies	Null: 1 study	Null: 3 studies
			Unfavourable: 0 studies	Unfavourable: 1 study	Unfavourable: 1 study
			Mixed: 0 studies	Mixed: 1 study	Mixed: 1 study
Psychosocial health	11	Very low to moderate	**Favourable: 2 studies**	Favourable: 3 studies	Favourable: 5 studies
			Null: 1 study	Null: 2 studies	Null: 3 studies
			Unfavourable: 0 studies	Unfavourable: 3 studies	Unfavourable: 3 studies
			Mixed: 0 studies	Mixed: 0 studies	Mixed: 0 studies
Cognitive development	13	Very low to high	**Favourable: 10 studies**	Favourable: 0 studies	**Favourable: 10 studies**
			Null: 0 studies	Null: 2 studies	Null: 2 studies
			Unfavourable: 0 studies	Unfavourable: 1 study	Unfavourable: 1 study
			Mixed: 0 studies	Mixed: 0 studies	Mixed: 0 studies
Fitness	3	Very low	Favourable: 0 studies	**Favourable: 3 studies**	**Favourable: 3 studies**
			Null: 0 studies	Null: 0 studies	Null: 0 studies
			Unfavourable: 0 studies	Unfavourable: 0 studies	Unfavourable: 0 studies
			Mixed: 0 studies	Mixed: 0 studies	Mixed: 0 studies
Important					
Bone and skeletal health	7	Very low to low	Favourable: 0 studies	**Favourable: 5 studies**	**Favourable: 5 studies**
			Null: 1 study	Null: 1 study	Null: 2 studies
			Unfavourable: 0 studies	Unfavourable: 0 studies	Unfavourable: 0 studies
			Mixed: 0 studies	Mixed: 0 studies	Mixed: 0 studies
Cardiometabolic health	9	Very low	**Favourable: 1 study**	Favourable: 1 study	Favourable: 2 studies
			Null: 0 studies	Null: 4 studies	Null: 4 studies
			Unfavourable: 0 studies	Unfavourable: 1 study	Unfavourable: 1 study
			Mixed: 0 studies	Mixed: 2 studies	Mixed: 2 studies
Risks/harm	4	Very low to low	Favourable: 0 studies	Favourable: 1 study	Favourable: 1 study
			Null: 0 studies	Null: 1 study	Null: 1 study
			Unfavourable: 0 studies	Unfavourable: 1 study	Unfavourable: 1 study
			Mixed: 0 studies	Mixed: 1 study	Mixed: 1 study

^a^Favourable: at least one favourable but no unfavourable associations were observed; Unfavourable: at least one unfavourable but no favourable associations were observed; Null: no favourable or unfavourable associations were observed; Mixed: both favourable and unfavourable or favourable, unfavourable, and null associations were all observed. **Bold font indicates ≥ 60% of studies were in the favourable or unfavourable direction**

Where possible, evidence on the association between the dose (i.e., frequency, intensity, duration, and type) of physical activity and health indicators was also synthesized. Various frequencies of physical activity were associated with health indicators, but the most favourable frequency of physical activity to obtain health benefits was unclear. In regard to intensity of physical activity, LPA and MPA were not consistently associated with any health indicators; whereas TPA, MVPA, and VPA were consistently associated with multiple health indicators. In terms of duration of physical activity, the evidence indicated that for infants, ≥30 min per day in the prone position accumulated throughout waking hours was associated with health benefits. However, for toddlers and preschoolers, the duration of physical activity needed to obtain health benefits was unclear. In regard to type of physical activity, consistent favourable associations with at least one health indicator were observed across multiple studies for a variety of different types of physical activity, including active play, aerobic, dance, prone position (infants), and structured/organized. Finally, some evidence existed to indicate that more physical activity was associated with greater health benefits.

This review builds on a previous systematic review conducted in 2012 that synthesized the evidence from 22 studies on the association between physical activity and health indicators among infants, toddlers, and preschoolers [7]. In contrast to the previous review (where cross-sectional study designs were excluded a priori), the present review included all study designs, thereby greatly increasing the evidence base; specifically, 55 cross-sectional studies were included. Not surprisingly, the evidence base also increased with time: a total of 45 of the studies included in the present study were published in 2012 or later (27 of those were cross-sectional). Due to a more comprehensive search strategy compared to the previous review, 17 additional studies, published in 2011 or earlier were also included. However, 12 studies included in the previous review were excluded in the present review because of changes in inclusion criteria (e.g., sample size) [132–143].

Despite the differences in the studies considered, the present review gathered similar results as the previous review [7]: both found that physical activity was favourably associated with motor development, cognitive development, psychosocial health, bone and skeletal health, and cardiometabolic health. This is in line with other reviews that have been published since 2012 on physical activity and single health indicators, including cognitive development in early childhood (aged 0-6 years) [19], psychosocial health in early childhood [21], and motor development in preschoolers [22]. However, it was acknowledged in both the psychosocial health and cognitive development reviews that the evidence was limited [19, 21].

In contrast to the review published by Timmons and colleagues in 2012 [7], favourable associations were not consistently observed between physical activity and adiposity in the present review. Although some favourable associations were observed, a large number of null associations were also observed, as well as some unfavourable and mixed associations (Table 9). Furthermore, no significant differences in BMI between intervention and control groups were observed in the meta-analysis of four studies. It is important to note that the bulk of studies used surrogate adiposity measures, such as BMI, whereas bioelectrical impedance [40], air-displacement plethysmography via the pediatric option for the BodPod [55], or dual energy X-ray absorptiometry [46, 49, 89] were used to measure adiposity in only five out of the 57 studies. Furthermore, the use of subjective physical activity measures that had unknown psychometric properties and neglecting to account for potential confounders (e.g., diet) within analyses were commonly identified risks of bias that may have affected the findings (Table 1). Alternatively, it could be that physical activity is not strongly associated with adiposity in the early years, and other factors such as diet and sleep are more important predictors in this age group [144, 145]. This conclusion is partly supported by the clearer evidence for the impact of physical activity on more rapidly developing health indicators such as motor development, psychosocial health, and cognitive development in the present review, where similar risk of bias limitations existed.

To better understand the commonly examined relationship between physical activity and adiposity in the early years, there is no need for more low-quality evidence; rather, what is needed is higher-quality evidence from strong study designs that address current limitations, including the use of objective measures of physical activity, direct measures of adiposity, and study designs or analyses that account for potential confounding factors. Given that adiposity was by far the most commonly studied health indicator, the focus of future high-quality physical activity and adiposity research should be balanced with the need for high-quality research that includes other health indicators in this age group.

Given that the current review included substantially more evidence than previous reviews, sub-analyses on the dose of physical activity, including frequency, intensity, duration, and type were possible. Intensity of physical activity was commonly examined in observational studies and in three experimental studies [111, 112, 115]. Previous research has shown that most of the physical activity that preschool-aged children participate in is of light intensity [11, 16, 17]. For instance, it was reported in a systematic review examining objectively measured physical activity and sedentary time that preschoolers spent an average of 2.2 h per day in LPA compared to 47 min per day in MVPA [11]. Interestingly, in the present review, the higher intensities of physical activity (MVPA and VPA), but not the lower intensities of physical activity (MPA and LPA), were consistently associated with multiple health indicators. However, it is important to note that most TPA consists of LPA, and several favourable relationships between TPA and health indicators were observed. Moreover, the majority of this evidence was in preschool-age samples. Overall, these findings suggest that some developmentally appropriate MVPA may be needed for health benefits at least for preschool-aged children, while acknowledging the inherent limitations of accelerometer cut-points to distinguish different intensities of physical activity [146]. In terms of LPA, there is some evidence in youth [147] and in adults [148] that physical activity at the higher end of the LPA spectrum compared to the lower end of the spectrum may be more beneficial for health but this is masked when looking only at total LPA. Future research should examine whether this is also the case for the early years, including infants, toddlers, and preschoolers. Such knowledge will help to determine whether activities at the upper end of the LPA spectrum should be targeted and promoted over lower intensities for health benefits in the early years.

Across both observational and experimental studies in the present review, a wide variety of types of physical activity were examined. The finding that structured/organized physical activity was favourably associated with health indicators in the present review is consistent with a recent systematic review on organized physical activity and health in preschool children [23]. In contrast to another recent systematic review, which reported favourable associations between risky outdoor play (i.e., play where children can disappear/get lost, great heights, rough-and-tumble play) and health [20], primarily null associations were observed between rough-and-tumble play or outdoor play and health indicators in the present study. However, it is important to note that the age groups differed between the two reviews, with the risky outdoor play review including children aged 3-12 years. Furthermore, the outdoor physical activity studies in the present review were not focused on “risky” outdoor play per se. Nevertheless, the favourable associations between a number of different types of physical activity and health indicators suggest that children in the early years should participate in a variety of physical activities for the most health benefits.

It was difficult to draw conclusions about the specific frequency or duration of physical activity that is needed for health benefits because only a small proportion of the included studies examined these dose parameters. Furthermore, most observational studies dichotomized physical activity frequency or duration, and no clear pattern was observed across studies for toddlers and preschoolers. For experimental studies, most involved a single- or two-arm intervention. Additionally, it was not possible to quantify total daily frequency or duration of physical activity because physical activity outside of the intervention was not usually taken into account or even measured. Despite these limitations, sparse but consistent evidence in infants indicates that at least 30 min of prone position or tummy time per day accumulated during waking hours appears beneficial, in particular for motor development. This aligns with the recommendation from the Canadian Paediatric Society, which suggests at least 10-15 min of tummy time, three times per day [149]. Unfortunately, it is not possible to draw specific conclusions on frequency and duration of physical activity for health benefits in toddlers and preschoolers. Current physical activity guidelines in Canada, Australia, and the United Kingdom recommend accumulating 180 min per day of any intensity in these age groups. According to the previously mentioned review by Hnatiuk and colleagues [11], this daily recommendation does align with average prevalence estimates in preschool-aged children (2.2 h or 132 min of LPA + 47 min of MVPA).

Despite not being able to make specific conclusions regarding frequency and duration of physical activity needed for health benefits in toddlers and preschoolers, some evidence existed across age groups that more physical activity is better for health. Specifically, this was supported in 13 studies by dose-response evidence or an exposure/outcome gradient for cross-sectional studies, primarily through continuous data [44, 49, 55, 57, 58, 67, 88, 89, 100, 104, 119, 123, 129]. Furthermore, 20 out of the 24 included experimental studies observed favourable associations with at least one health indicator. Although some behaviour compensation could have occurred, it is likely that the majority of the physical activity accumulated as part of the intervention was in addition to children’s baseline physical activity. Therefore, this experimental evidence also supports the overall conclusion that more physical activity is better for health. However, to understand the specific frequency and duration of physical activity needed for health benefits across the early years, further dose-response evidence is needed. Specifically, this should include observational studies that compare multiple categories of physical activity frequency and duration in relation to health indicators, and experimental studies that compare multiple intervention arms with different frequency and duration of physical activity in relation to health indicators. Experimental studies should also take into account baseline physical activity levels.

This discussion has already highlighted a number of research gaps and limitations that need to be addressed by future research; however, there are additional gaps and limitations that also warrant attention. For instance, most of the evidence included in this review was based on preschool-aged children. Given the vast developmental differences in early years age groups [6], the findings observed in preschool-aged children may not be generalizable to infants and toddlers. Therefore, future research should examine the relationships between physical activity and health indicators specifically in infants and toddlers to ensure developmentally appropriate doses of physical activity are being identified, recommended, and promoted. Part of this work may involve determining how to most accurately measure physical activity in infants and toddlers. In fact, objective measures of physical activity were used in only two studies with samples classified as infants or toddlers [86, 118], although the measurement of physical activity was a limitation observed across all age groups in the present review. Specifically, as noted in the risk of bias assessments, subjective measures of physical activity with unknown psychometric properties were commonly used. It is known that the sporadic and intermittent nature of physical activity in the early years makes it difficult to accurately capture physical activity with subjective measures [146]. Furthermore, although objective measures of physical activity were used in 35 studies, primarily with accelerometers, heterogeneity in data collection (e.g., monitor placement, epoch length) and reduction (non-wear time definitions, removal of naps, cut-points) procedures across studies may have contributed to the inconsistency of some findings. This may explain why similar conclusions were found across health indicators in the present review when comparing studies that used objective versus subjective measures of TPA as the exposure. Therefore, identifying the most appropriate accelerometer data collection and reduction procedures for early years children should be explored in future research so that these procedures can be standardized across studies. Furthermore, among the 24 experimental studies, 15 did not measure physical activity, so it was unclear if the intervention was in fact successful in changing physical activity levels. Therefore, baseline and follow-up measures of physical activity should be included in future interventions.

Along with the evidence gaps and limitations associated with age groups studied and physical activity measurement, limited studies were available for a number of the health indicators. For example, there were 10 or fewer included studies for each of the following health indicators: psychosocial health, fitness, bone and skeletal health, cardiometabolic health, and risks/harm. Future high-quality research that increases the evidence base for these health indicators is needed. Additionally, while only three studies were included for fitness, some overlap existed between fitness and motor development categories (e.g., standing long jump versus standing broad jump; 12-m run versus 20-m shuttle run). Consensus is needed on what measures constitute fitness versus motor development in this age group.

One strength of the present systematic review was the use of a comprehensive search strategy that was both developed and peer-reviewed by librarians with expertise in systematic reviews. Another strength was the broad scope of the review through the inclusion of all study designs, both subjective and objective measures of physical activity, multiple health indicators, and multiple age groups (i.e. infants, toddlers, and preschoolers). Furthermore, the conduct of sub-analyses on dose of physical activity was a notable strength of the review, as was the meta-analysis of four adiposity interventions. Finally, the use of the established GRADE framework to guide the review and assess the quality of evidence was an additional strength [28].

The present review also had several limitations, including English and French language limits for feasibility, as well as sample size restrictions for both feasibility and generalizability. It is possible that studies published in other languages or with smaller sample sizes might have provided additional insight, especially for health indicators where evidence was limited. Furthermore, while a meta-analysis was conducted on four included studies, due to the large heterogeneity of the study designs and measured outcomes, the majority of findings were based on a narrative synthesis that weighted all studies equally. For some health indicators, conclusions from the narrative synthesis had to be drawn from a small number of studies. Furthermore, it was not possible to do sensitivity analyses between higher- and lower-quality evidence because the vast majority of evidence was “low” to “very low” quality.

## Conclusions

This review synthesized evidence from 96 studies on the health implications of physical activity in the early years. Physical activity was consistently found to be favourably associated with a broad range of health indicators. Several types of physical activity, especially prone position for infants, TPA, and physical activity of at least moderate to vigorous intensity, particularly for preschool-aged children, were consistently found to be favourable with a number of health indicators. Although it was not possible to identify the specific frequency and duration of physical activity needed for health benefits in all age groups, it was consistently observed that more physical activity (in terms of frequency or duration) was better for health. Therefore, it can be concluded that it is important to promote physical activity in the early years. The findings of this review will help to inform evidence-based guidelines to facilitate physical activity promotion aimed at optimizing the overall health of our youngest children. Given that the study of physical activity in the early years is still a relatively new area of inquiry, future research should focus on addressing a number of gaps and limitations mentioned in this review, in order to strengthen the evidence base and accurately inform future health promotion efforts.

## Additional files

Additional file 1: Search strategies for the systematic review. (DOCX 37 kb)

Additional file 2: Supplementary Tables S1-S8. Summary of studies included in the systematic review for each health indicator sorted by (whenever possible) study design, age group, and physical activity measurement. (DOCX 177 kb)

## Abbreviations

BMI: Body mass index
GRADE: Grading of recommendations assessment, development, and evaluation
LPA: Light-intensity physical activity
MPA: Moderate-intensity physical activity
MVPA: Moderate- to vigorous-intensity physical activity
PICO: Population, intervention, comparison, and outcome
PRISMA: Preferred reporting items for systematic reviews and meta-analyses
PROSPERO: International prospective register of systematic reviews
RCT: Randomized controlled trial
TPA: Total physical activity
VPA: Vigorous-intensity physical activity

## References

[1] Janssen I, Leblanc AG (2010). Systematic review of the health benefits of physical activity and fitness in school-aged children and youth. Int J Behav Nutr Phys Act.

[2] Poitras VJ, Gray CE, Borghese MM, Carson V, Chaput JP, Janssen I (2016). Systematic review of the relationships between objectively measured physical activity and health indicators in school-aged children and youth. Appl Physiol Nutr Metab..

[3] Warburton DE, Nicol CW, Bredin SS (2006). Health benefits of physical activity: the evidence. Can Med Assoc J.

[4] US Department of Health and Human Services. Physical activity guidelines for Americans. 2008. https://health.gov/paguidelines/guidelines/. Accessed 4 Jan 2016.

[5] World Health Organization. Global recommendations on physical activity for health. World Health Organization, Geneva, Switzerland. 2010. http://www.who.int/dietphysicalactivity/publications/9789241599979/en/. Accessed 4 Jan 2016.

[6] Berk L. Development through the lifespan. 6th ed. Boston, MA: Pearson Higher Education; 2013.

[7] Timmons BW, LeBlanc AG, Carson V, Connor Gorber S, Dillman C, Janssen I (2012). Systematic review of physical activity and health in the early years (aged 0–4 years). Appl Physiol Nutr Metab..

[8] Tremblay MS, LeBlanc AG, Carson V, Choquette L, Connor Gorber S, Dillman C (2012). Canadian physical activity guidelines for the early years (aged 0–4 years). Appl Physiol Nutr Metab.

[9] Department of Health and Aging (DoAH) (2010). Move and play every day. National physical activity recommendations for children 0–5 years. Physical activity recommendations for 0-5 year olds.

[10] Department of Health Physical Activity Health Improvement and Protection (2011). Start active stay active: a report on physical activity from the four home countries' chief medical officers. London, England: Department of Health, physical activity, health improvement and protection.

[11] Hnatiuk JA, Salmon J, Hinkley T, Okely AD, Trost S (2014). A review of preschool children's physical activity and sedentary time using objective measures. Am J Prev Med.

[12] Bingham DD, Costa S, Hinkley T, Shire KA, Clemes SA, Barber SE (2016). Physical activity during the early years: a systematic review of correlates and determinants. Am J Prev Med.

[13] Vanderloo LM, Tucker P (2015). An objective assessment of toddlers' physical activity and sedentary levels: a cross-sectional study. BMC Public Health.

[14] Wijtzes AI, Kooijman MN, Kiefte-de Jong JC, de Vries SI, Henrichs J, Jansen W (2013). Correlates of physical activity in 2-year-old toddlers: the generation R study. J Pediatr.

[15] Hnatiuk J, Ridgers ND, Salmon J, Campbell K, McCallum Z, Hesketh K (2012). Physical activity levels and patterns of 19-month-old children. Med Sci Sport Exerc.

[16] Colley RC, Garriguet D, Adamo KB, Carson V, Janssen I, Timmons BW (2013). Physical activity and sedentary behavior during the early years in Canada: a cross-sectional study. Int J Behav Nutr Phys Act.

[17] Hesketh KR, McMinn AM, Ekelund U, Sharp SJ, Collings PJ, Harvey NC (2014). Objectively measured physical activity in four-year-old British children: a cross-sectional analysis of activity patterns segmented across the day. Int J Behav Nutr Phys Act.

[18] Shekelle P, Woolf S, Grimshaw JM, Schünemann HJ, Eccles MP (2012). Developing clinical practice guidelines: reviewing, reporting, and publishing guidelines; updating guidelines; and the emerging issues of enhancing guideline implementability and accounting for comorbid conditions in guideline development. Implement Sci.

[19] Carson V, Hunter S, Kuzik N, Wiebe SA, Spence JC, Friedman A (2016). Systematic review of physical activity and cognitive development in early childhood. J Sci Med Sport.

[20] Brussoni M, Gibbons R, Gray C, Ishikawa T, Sandseter EBH, Bienenstock A (2015). What is the relationship between risky outdoor play and health in children? A systematic review. Int J Environ Res Public Health.

[21] Hinkley T, Teychenne M, Downing KL, Ball K, Salmon J, Hesketh KD (2014). Early childhood physical activity, sedentary behaviors and psychosocial well-being: a systematic review. Prev Med.

[22] Figueroa R, An R (2017). Motor skill competence and physical activity in preschoolers: a review. Matern Child Health J.

[23] Venetsanou F, Kambas A, Giannakidou D (2015). Organized physical activity and health in preschool age: a review. Cent Eur J Public Health.

[24] Moher D, Liberati A, Tetzlaff J, Altman DG, Group P (2009). Preferred reporting items for systematic reviews and meta-analyses: the PRISMA statement. PLoS Med.

[25] Schardt C, Adams MB, Owens T, Keitz S, Fontelo P (2007). Utilization of the PICO framework to improve searching PubMed for clinical questions. BMC Med Inform Decis Mak.

[26] Caspersen CJ, Powell KE, Christenson GM (1985). Physical activity, exercise, and physical fitness: definitions and distinctions for health-related research. Public Health Rep.

[27] Sirard JR, Pate RR (2001). Physical activity assessment in children and adolescents. Sports Med.

[28] Guyatt G, Oxman AD, Akl EA, Kunz R, Vist G, Brozek J (2011). GRADE guidelines: 1. Introduction—GRADE evidence profiles and summary of findings tables. J Clin Epidemiol.

[29] Guyatt G, Oxman AD, Sultan S, Glasziou P, Akl EA, Alonso-Coello P (2011). GRADE guidelines: 9. Rating up the quality of evidence. J Clin Epidemiol.

[30] Guyatt GH, Oxman AD, Schünemann HJ, Tugwell P, Knottnerus A (2011). GRADE guidelines: a new series of articles in the journal of clinical epidemiology. J Clin Epidemiol.

[31] Higgins JP, Green S. Cochrane handbook for systematic reviews of interventions, vol. 4. West Sussex, England: John Wiley & Sons; 2011.

[32] Guyatt G, Oxman AD, Vist G, Kunz R, Brozek J, Alonso-Coello P (2011). GRADE guidelines: 4. Rating the quality of evidence—study limitations (risk of bias). J Clin Epidemiol.

[33] Jones RA, Riethmuller A, Hesketh K, Trezise J, Batterham M, Okely AD (2011). Promoting fundamental movement skill development and physical activity in early childhood settings: a cluster randomized controlled trial. Pediatr Exerc Sci.

[34] Annesi JJ, Smith AE, Tennant GA (2013). Effects of a cognitive–behaviorally based physical activity treatment for 4- and 5-year-old children attending US preschools. Int J Behav Med.

[35] Mo-suwan L, Pongprapai S, Junjana C, Puetpaiboon A (1998). Effects of a controlled trial of a school-based exercise program on the obesity indexes of preschool children. Am J Clin Nutr.

[36] Krombholz H (2012). The impact of a 20-month physical activity intervention in child care centers on motor performance and weight in overweight and healthy-weight preschool children. Percept Mot Skills.

[37] Higgins JPT, Deeks JJ, editors. Chapter 7.7.3.3: Obtaining standard deviations from standard errors, confidence intervals, t values and P values for differences in means. In: Higgins JPT, Green S, editors. Cochrane handbook for systematic reviews of interventions, Version 5.1.0 (updated March 2011). The Cochrane Collaboration. 2011. www.handbook.cochrane.org. Accessed 4 Jan 2016.

[38] DerSimonian R, Laird N (1986). Meta-analysis in clinical trials. Controlled. Clin Trials.

[39] Higgins JPT, Deeks JJ, editors. Chapter 9.4.3.1: Random-effects (DerSimonian and Laird) method for meta-analysis. In: Higgins JPT, Green S, editors. Cochrane handbook for systematic reviews of interventions, Version 5.1.0 (updated March 2011). The Cochrane Collaboration. 2011. www.handbook.cochrane.org. Accessed 16 Jan 2016.

[40] de Vries A, Huiting H, Heuvel E, L'Abée C, Corpeleijn E, Stolk R (2015). An activity stimulation programme during a childs first year reduces some indicators of adiposity at the age of two-and-a-half. Acta Paediatr.

[41] Bonvin A, Barral J, Kakebeeke TH, Kriemler S, Longchamp A, Schindler C (2013). Effect of a governmentally-led physical activity program on motor skills in young children attending child care centers: a cluster randomized controlled trial. Int J Behav Nutr Phys Act.

[42] Monsalves Álvarez M, Castro Sepúlveda M, Zapata Lamana R, Rosales Soto G, Salazar G (2015). Motor skills and nutritional status outcomes from a physical activity intervention in short breaks on preschool children conducted by their educators: a pilot study. Nutr Hosp.

[43] DuRant RH, Baranowski T, Rhodes T, Gutin B, Thompson WO, Carroll R (1993). Association among serum lipid and lipoprotein concentrations and physical activity, physical fitness, and body composition in young children. J Pediatr.

[44] Klesges RC, Klesges LM, Eck LH, Shelton ML (1995). A longitudinal analysis of accelerated weight gain in preschool children. Pediatr..

[45] Sijtsma A, Sauer PJ, Stolk RP, Corpeleijn E (2013). Infant movement opportunities are related to early growth—GECKO Drenthe cohort. Early Hum Dev.

[46] Carter PJ, Taylor BJ, Williams SM, Taylor RW (2011). Longitudinal analysis of sleep in relation to BMI and body fat in children: the FLAME study. BMJ.

[47] de Coen V, De Bourdeaudhuij I, Verbestel V, Maes L, Vereecken C (2014). Risk factors for childhood overweight: a 30-month longitudinal study of 3- to 6-year-old children. Public Health Nutr.

[48] Huynh DT, Dibley MJ, Sibbritt D, Tran H, Le QT (2011). Influence of contextual and individual level risk factors on adiposity in a preschool child cohort in ho chi Minh City, Vietnam. Pediatr Obes.

[49] Butte NF, Puyau MR, Wilson TA, Liu Y, Wong WW, Adolph AL (2016). Role of physical activity and sleep duration in growth and body composition of preschool-aged children. Obesity.

[50] Sijtsma A, Sauer PJ, Corpeleijn E (2015). Parental correlations of physical activity and body mass index in young children—the GECKO Drenthe cohort. Int J Behav Nutr Phys Act.

[51] Takahashi E, Yoshida K, Sugimori H, Miyakawa M, Izuno T, Yamagami T (1999). Influence factors on the development of obesity in 3-year-old children based on the Toyama study. Prev Med.

[52] Kain J, Andrade M (1999). Characteristics of the diet and patterns of physical activity in obese Chilean preschoolers. Nutr Res.

[53] He Q, Ding Z, Fong D, Karlberg J (2000). Risk factors of obesity in preschool children in China: a population-based case-control study. Int J Obes.

[54] Eijkemans M, Mommers M, de Vries SI, van Buuren S, Stafleu A, Bakker I (2008). Asthmatic symptoms, physical activity, and overweight in young children: a cohort study. Pediatr..

[55] Leppänen M, Nyström CD, Henriksson P, Pomeroy J, Ruiz J, Ortega F (2016). Physical activity intensity, sedentary behavior, body composition and physical fitness in 4-year-old children: results from the MINISTOP trial. Int J Obes.

[56] Lin LY, Cherng RJ, Chen YJ. Relationship between time use in physical activity and gross motor performance of preschool children. Aust Occup Ther J. 2016; 10.1111/1440-1630.12318. 10.1111/1440-1630.12318 27427505

[57] Pallan MJ, Adab P, Sitch AJ, Aveyard P (2014). Are school physical activity characteristics associated with weight status in primary school children? A multilevel cross-sectional analysis of routine surveillance data. Arch Dis Child.

[58] Ansari A, Pettit K, Gershoff E (2015). Combating obesity in head start: outdoor play and change in children's body mass index. J Dev Behav Pediatr.

[59] Lioret S, Maire B, Volatier J, Charles M (2007). Child overweight in France and its relationship with physical activity, sedentary behaviour and socioeconomic status. Eur J Clin Nutr.

[60] Trost SG, Sirard JR, Dowda M, Pfeiffer KA, Pate RR (2003). Physical activity in overweight and nonoverweight preschool children. Int J Obes.

[61] Kagamimori S, Yamagami T, Sokejima S, Numata N, Handa K, Nanri S (1999). The relationship between lifestyle, social characteristics and obesity in 3-year-old Japanese children. Child Care Health Dev.

[62] Nelson JA, Carpenter K, Chiasson MA (2006). Diet, activity, and overweight among preschool-age children enrolled in the special supplemental nutrition program for women, infants, and children (WIC). Prev Chronic Dis.

[63] Chen LP, Ziegenfuss JY, Jenkins SM, Beebe TJ, Ytterberg KL (2011). Pediatric obesity and self-reported health behavior information. Clin Pediatr.

[64] Shapiro LR, Crawford PB, Clark MJ, Pearson DL, Raz J, Huenemann RL (1984). Obesity prognosis: a longitudinal study of children from the age of 6 months to 9 years. Am J Public Health.

[65] Jones RA, Okely AD, Gregory P, Cliff DP (2009). Relationships between weight status and child, parent and community characteristics in preschool children. Int J Pediatr Obes.

[66] Klesges RC, Haddock CK, Eck LH (1990). A multimethod approach to the measurement of childhood physical activity and its relationship to blood pressure and body weight. J Pediatr.

[67] Williams HG, Pfeiffer KA, O'Neill JR, Dowda M, McIver KL, Brown WH (2008). Motor skill performance and physical activity in preschool children. Obesity.

[68] Jouret B, Ahluwalia N, Cristini C, Dupuy M, Nègre-Pages L, Grandjean H (2007). Factors associated with overweight in preschool-age children in southwestern France. Am J Clin Nutr.

[69] Pfeiffer KA, Dowda M, McIver KL, Pate RR (2009). Factors related to objectively measured physical activity in preschool children. Pediatr Exerc Sci.

[70] de Carvalho Cremm E, Leite FHM, de Abreu DSC, de Oliveira MA, Scagliusi FB, Martins PA (2012). Factors associated with overweight in children living in the neighbourhoods of an urban area of Brazil. Public Health Nutr.

[71] Anderson SE, Economos CD, Must A (2008). Active play and screen time in US children aged 4 to 11 years in relation to sociodemographic and weight status characteristics: a nationally representative cross-sectional analysis. BMC Public Health.

[72] Sääkslahti A, Numminen P, Varstala V, Helenius H, Tammi A, Viikari J (2004). Physical activity as a preventive measure for coronary heart disease risk factors in early childhood. Scand J Med Sci Sports.

[73] Cardon G, De Bourdeaudhuij I, Iotova V, Latomme J, Socha P, Koletzko B (2016). Health related behaviours in normal weight and overweight preschoolers of a large pan-European sample: the ToyBox-study. PLoS One.

[74] Jiang J, Rosenqvist U, Wang H, Greiner T, Ma Y, Toschke AM (2006). Risk factors for overweight in 2- to 6-year-old children in Beijing, China. Int J Pediatr Obes.

[75] Söderström M, Boldemann C, Sahlin U, Mårtensson F, Raustorp A, Blennow M (2013). The quality of the outdoor environment influences childrens health—a cross-sectional study of preschools. Acta Paediatr.

[76] Cox R, Skouteris H, Rutherford L, Fuller-Tyszkiewicz M, Hardy LL (2012). Television viewing, television content, food intake, physical activity and body mass index: a cross-sectional study of preschool children aged 2-6 years. Health Promot J Austr.

[77] Jago R, Baranowski T, Baranowski JC, Thompson D, Greaves K (2005). BMI from 3-6 y of age is predicted by TV viewing and physical activity, not diet. Int J Obes.

[78] Hajian-Tilaki K, Heidari B (2013). Childhood obesity, overweight, socio-demographic and life style determinants among preschool children in Babol, northern Iran. Iran J Public Health.

[79] Watanabe E, Lee J, Kawakubo K (2011). Associations of maternal employment and three-generation families with pre-school children's overweight and obesity in Japan. Int J Obes.

[80] Sijtsma A, Koller M, Sauer PJ, Corpeleijn E (2015). Television, sleep, outdoor play and BMI in young children: the GECKO Drenthe cohort. Eur J Pediatr.

[81] Sääkslahti A, Numminen P, Niinikoski H, Rask-Nissilä L, Viikari J, Tuominen J (1999). Is physical activity related to body size, fundamental motor skills, and CHD risk factors in early childhood?. Pediatr Exerc Sci.

[82] Bonvin A, Barral J, Kakebeeke TH, Kriemler S, Longchamp A, Marques-Vidal P (2012). Weight status and gender-related differences in motor skills and in child care-based physical activity in young children. BMC Pediatr.

[83] Burdette HL, Whitaker RC (2005). A national study of neighborhood safety, outdoor play, television viewing, and obesity in preschool children. Pediatr..

[84] Kuzik N, Carson V (2016). The association between physical activity, sedentary behavior, sleep, and body mass index z-scores in different settings among toddlers and preschoolers. BMC Pediatr.

[85] Østbye T, Malhotra R, Stroo M, Lovelady C, Brouwer R, Zucker N (2013). The effect of the home environment on physical activity and dietary intake in preschool children. Int J Obes.

[86] Johansson E, Hagströmer M, Svensson V, Ek A, Forssén M, Nero H, Marcus C (2015). Objectively measured physical activity in two-year-old children—levels, patterns and correlates. Int J Behav Nutr Phys Act.

[87] LaRowe TL, Adams AK, Jobe JB, Cronin KA, Vannatter SM, Prince RJ (2010). Dietary intakes and physical activity among preschool-aged children living in rural American Indian communities before a family-based healthy lifestyle intervention. J Am Diet Assoc.

[88] España-Romero V, Mitchell JA, Dowda M, O'Neill JR, Pate RR (2013). Objectively measured sedentary time, physical activity and markers of body fat in preschool children. Pediatr Exerc Sci.

[89] Collings PJ, Brage S, Ridgway CL, Harvey NC, Godfrey KM, Inskip HM (2013). Physical activity intensity, sedentary time, and body composition in preschoolers. Am J Clin Nutr.

[90] Porter LS (1972). The impact of physical-physiological activity on infants' growth and development. Nursing Res.

[91] Teixeira Costa HJ, Abelairas-Gomez C, Arufe-Giráldez V, Pazos-Couto JM, Barcala-Furelos R (2015). Influence of a physical education plan on psychomotor development profiles of preschool children. J Human Sport Exerc.

[92] Mostafavi R, Ziaee V, Akbari H, Haji-Hosseini S (2014). The effects of spark physical education program on fundamental motor skills in 4-6 year-old children. Iran J Pediatr.

[93] Draper CE, Achmat M, Forbes J, Lambert EV (2012). Impact of a community-based programme for motor development on gross motor skills and cognitive function in preschool children from disadvantaged settings. Early Child Dev Care.

[94] Livonen S, Sääkslahti A, Nissinen K (2011). The development of fundamental motor skills of four- to five-year-old preschool children and the effects of a preschool physical education curriculum. Early Child Dev Care.

[95] Venetsanou F, Kambas A (2004). How can a traditional Greek dances programme affect the motor proficiency of pre-school children?. Research in Dance Education.

[96] Sigmundsson H, Hopkins B (2010). Baby swimming: exploring the effects of early intervention on subsequent motor abilities. Child Care Health Dev.

[97] Kuo Y-L, Liao H-F, Chen P-C, Hsieh W-S, Hwang A-W (2008). The influence of wakeful prone positioning on motor development during the early life. J Dev Behav Pediatr.

[98] de Kegel A, Peersman W, Onderbeke K, Baetens T, Dhooge I (2013). New reference values must be established for the Alberta infant motor scales for accurate identification of infants at risk for motor developmental delay in Flanders. Child Care Health Dev.

[99] Dudek-Shriber L, Zelazny S (2007). The effects of prone positioning on the quality and acquisition of developmental milestones in four-month-old infants. Pediatr Phys Ther.

[100] Fisher A, Reilly JJ, Kelly LA, Montgomery C, Williamson A, Paton JY (2005). Fundamental movement skills and habitual physical activity in young children. Med Sci Sports Exerc.

[101] Matheny AP, Brown AM (1971). Activity, motor coordination and attention: individual differences in twins. Percept Mot Skills.

[102] Lobo YB, Winsler A (2006). The effects of a creative dance and movement program on the social competence of head start preschoolers. Soc Dev.

[103] Vella SA, Cliff DP, Magee CA, Okely AD (2015). Associations between sports participation and psychological difficulties during childhood: a two-year follow up. J Sci Med Sport.

[104] Wang H, Sekine M, Chen X, Yamagami T, Kagamimori S (2008). Lifestyle at 3 years of age and quality of life (QOL) in first-year junior high school students in Japan: results of the Toyama birth cohort study. Qual Life Res.

[105] Lindsay H, Brussoni M (2014). Injuries and helmet use related to non-motorized wheeled activities among pediatric patients. Chronic Dis Inj Canada.

[106] BN Y, Protudjer JLP, Anderson K, Fieldhouse P (2010). Weight status and determinants of health in Manitoba children and youth. Can J Diet Pract Res.

[107] ML Y, Ziviani J, Baxter J, Haynes M (2012). Time use differences in activity participation among children 4–5 years old with and without the risk of developing conduct problems. Res Dev Disabil.

[108] Fliek L, Daemen E, Roelofs J, Muris P (2015). Rough-and-tumble play and other parental factors as correlates of anxiety symptoms in preschool children. J Child Fam Stud.

[109] Irwin JD, Johnson AM, Vanderloo LM, Burke SM, Tucker P (2015). Temperament and objectively measured physical activity and sedentary time among Canadian preschoolers. Prev Med Rep.

[110] Mavilidi M-F, Okely AD, Chandler P, Cliff DP, Paas F (2015). Effects of integrated physical exercises and gestures on preschool children's foreign language vocabulary learning. Educ Psychol Rev.

[111] Kirk SM, Vizcarra CR, Looney EC, Kirk EP (2014). Using physical activity to teach academic content: a study of the effects on literacy in head start preschoolers. Early Child Educ J.

[112] Kirk SM, Kirk EP (2016). Sixty minutes of physical activity per day included within preschool academic lessons improves early literacy. J Sch Health.

[113] Zachopoulou E, Trevlas E, Konstadinidou E (2006). Archimedes project research group. The design and implementation of a physical education program to promote children's creativity in the early years. Int J Early Years Educ.

[114] Palmer KK, Miller MW, Robinson LE (2013). Acute exercise enhances preschoolers' ability to sustain attention. J Sport Exerc Psychol.

[115] Webster EK, Wadsworth DD, Robinson LE (2015). Preschoolers' time on-task and physical activity during a classroom activity break. Pediatr Exerc Sci.

[116] Holmes RM, Pellegrini AD, Schmidt SL (2006). The effects of different recess timing regimens on preschoolers' classroom attention. Early Child Dev Care.

[117] Kolpakov V, Bespalova T, Tomilova E, Larkina NY, Mamchits E, Chernogrivova M (2011). Functional reserves and adaptive capacity of subjects with different levels of habitual physical activity. Human. Physiol.

[118] Specker BL, Mulligan L, Ho M (1999). Longitudinal study of calcium intake, physical activity, and bone mineral content in infants 6-18 months of age. J Bone Miner Res.

[119] Xu H, Zhao Z, Wang H, Ding M, Zhou A, Wang X (2013). Bone mineral density of the spine in 11,898 Chinese infants and young children: a cross-sectional study. PLoS One.

[120] Jazar AS, Takruri HR, Khuri-Bulos NA (2012). Vitamin D status in a sample of preschool children aged from 1 to 6 years visiting the pediatrics clinic at Jordan University hospital. Jordan Med J.

[121] Kensarah OA, Jazar AS, Azzeh FS (2015). Hypovitaminosis D in healthy toddlers and preschool children from western Saudi Arabia. Int J Vit Nutr Res.

[122] Harvey N, Cole Z, Crozier S, Kim M, Ntani G, Goodfellow L (2012). Physical activity, calcium intake and childhood bone mineral: a population-based cross-sectional study. Osteoporos Int.

[123] Herrmann D, Buck C, Sioen I, Kouride Y, Marild S, Molnár D (2015). Impact of physical activity, sedentary behaviour and muscle strength on bone stiffness in 2-10-year-old children—cross-sectional results from the IDEFICS study. Int J Behav Nutr Phys Act.

[124] Specker BL, Johannsen N, Binkley T, Finn K (2001). Total body bone mineral content and tibial cortical bone measures in preschool children. J Bone Miner Res.

[125] Scheffler C, Ketelhut K, Mohasseb I. Does physical education modify the body composition? Results of a longitudinal study of pre-school children. Anthropol Anz. 2007:193–201. 17711151

[126] Wilson DK, Klesges LM, Klesges RC, Eck LH, Hackett-Renner CA, Alpert BS (1992). A prospective study of familial aggregation of blood pressure in young children. J Clin Epidemiol.

[127] Jiménez-Pavón D, Konstabel K, Bergman P, Ahrens W, Pohlabeln H, Hadjigeorgiou C (2013). Physical activity and clustered cardiovascular disease risk factors in young children: a cross-sectional study (the IDEFICS study). BMC Med.

[128] Damashek A, Kuhn J (2012). Toddlers’ unintentional injuries: the role of maternal-reported paternal and maternal supervision. J Pediatr Psychol.

[129] Clark EM, Ness AR, Tobias JH (2008). Vigorous physical activity increases fracture risk in children irrespective of bone mass: a prospective study of the independent risk factors for fractures in healthy children. J Bone Miner Res.

[130] Hutchison BL, Thompson JM, Mitchell EA (2003). Determinants of nonsynostotic plagiocephaly: a case-control study. Pediatr..

[131] van Vlimmeren LA, van der Graaf Y, Boere-Boonekamp MM, L'Hoir MP, Helders PJ, Engelbert RH (2007). Risk factors for deformational plagiocephaly at birth and at 7 weeks of age: a prospective cohort study. Pediatr.

[132] Buss DM, Block JH, Block J. Preschool activity level: personality correlates and developmental implications. Child Dev. 1980:401–8.

[133] Li R, O'Connor L, Buckley D, Specker B (1995). Relation of activity levels to body fat in infants 6 to 12 months of age. J Pediatr.

[134] Metcalf BS, Jeffery AN, Hosking J, Voss LD, Sattar N, Wilkin TJ (2009). Objectively measured physical activity and its association with adiponectin and other novel metabolic markers. Diabetes Care.

[135] Metcalf BS, Voss LD, Hosking J, Jeffery AN, Wilkin TJ (2008). Physical activity at the government-recommended level and obesity-related health outcomes: a longitudinal study (early bird 37). Arch Dis Child.

[136] Moore LL, Gao D, Bradlee ML, Cupples LA, Sundarajan-Ramamurti A, Proctor MH (2003). Does early physical activity predict body fat change throughout childhood?. Prev Med.

[137] Moore LL, Nguyen U-SD, Rothman KJ, Cupples LA, Ellison RC (1995). Preschool physical activity level and change in body fatness in young children: the Framingham Children’s study. Am J Epidemiol.

[138] Reilly JJ, Kelly L, Montgomery C, Williamson A, Fisher A, McColl JH (2006). Physical activity to prevent obesity in young children: cluster randomised controlled trial. BMJ.

[139] Binkley T, Specker B (2004). Increased periosteal circumference remains present 12 months after an exercise intervention in preschool children. Bone.

[140] Specker B, Binkley T (2003). Randomized trial of physical activity and calcium supplementation on bone mineral content in 3- to 5-year-old children. J Bone Miner Res.

[141] Sugimori H, Yoshida K, Izuno T, Miyakawa M, Suka M, Sekine M (2004). Analysis of factors that influence body mass index from ages 3 to 6 years: a study based on the Toyama cohort study. Pediatr Int.

[142] Wells JC, Ritz P (2001). Physical activity at 9-12 months and fatness at 2 years of age. Am J Human Biol.

[143] Ku L, Shapiro L, Crawford P, Huenemann R (1981). Body composition and physical activity in 8-year-old children. Am J Clin Nutr.

[144] Monasta L, Batty G, Cattaneo A, Lutje V, Ronfani L, Van Lenthe F (2010). Early-life determinants of overweight and obesity: a review of systematic reviews. Obes Rev.

[145] Chaput JP, Gray CG, Poitras VJ, Carson V, Gruber R, Birken CS (2017). Systematic review of the relationships between sleep duration and health indicators in the early years (0-4 years). BMC Public Health.

[146] Cliff DP, Reilly JJ, Okely AD (2009). Methodological considerations in using accelerometers to assess habitual physical activity in children aged 0–5 years. J Sci Med Sport.

[147] Carson V, Ridgers ND, Howard BJ, Winkler EAH, Healy GN, Owen N (2013). Light-intensity physical activity and cardiometabolic biomarkers in US adolescents. PLoS One.

[148] Howard B, Winkler E, Sethi P, Carson V, Ridgers ND, Salmon J (2015). Associations of low- and high-intensity light activity with cardiometabolic biomarkers. Med Sci Sports Exer.

[149] Canadian Paediatric Society. Positional plagiocephaly. 2011. http://www.cps.ca/documents/position/positional-plagiocephaly#ref2. Accessed 4 Jan 2016.

