# Correction to: Systematic review of the relationships between physical activity and health indicators in the early years (0-4 years)

**DOI:** 10.1186/s12889-017-4981-5

**Published:** 2017-12-29

**Authors:** Valerie Carson, Eun-Young Lee, Lyndel Hewitt, Cally Jennings, Stephen Hunter, Nicholas Kuzik, Jodie A. Stearns, Stephanie Powley Unrau, Veronica J. Poitras, Casey Gray, Kristi B. Adamo, Ian Janssen, Anthony D. Okely, John C. Spence, Brian W. Timmons, Margaret Sampson, Mark S. Tremblay

**Affiliations:** 1 Faculty of Physical Education and Recreation, University of Alberta, Edmonton, AB T6G 2H9 Canada; 2 Early Start Research Institute, Faculty of Social Sciences, University of Wollongong, Wollongong, NSW 2522 Australia; 3 Healthy Active Living and Obesity Research Group, Children’s Hospital of Eastern Ontario Research Institute, Ottawa, ON K1H 8L1 Canada; 4 School of Human Kinetics, Faculty of Health Sciences, University of Ottawa, Ottawa, ON K1N 1A2 Canada; 5 School of Kinesiology and Health Studies, and Department of Public Health Sciences, Queen’s University, Kingston, ON K7L 3N6 Canada; 6 Child Health & Exercise Medicine Program, Department of Pediatrics, McMaster University, Hamilton, ON L8S 4K1 Canada; 7 Library and Media Services, Children’s Hospital of Eastern Ontario, Ottawa, ON K1H 8L1 Canada

## Correction

After publication of the article [1], it has been brought to our attention that an incorrect reference has been used in this article, both in the main body and additional file 2. The reference in question is #105 in the main body and #74 in additional file 2. Here it is cited as “Lindsay H, Brussoni M. Injuries and helmet use related to non-motorized wheeled activities among pediatric patients. Chronic Dis Inj Canada. 2014;34(2–3):74–81”.

The authors would like to indicate it should be “Lindsey, E. Physical activity play and preschool children’s peer acceptance: Distinctions between rough-and-tumble and exercise play. Early Educ Dev. 2014;25(3): 297–294”.

## References

[1] Carson V, Lee E, Hewitt L, Jennings C, Hunter S, Kuzik N (2017). Systematic review of the relationships between physical activity and health indicators in the early years (0-4 years). BMC Public Health.

