# Supplementary material for: Systematic review of the relationships between physical activity and health indicators in the early years (0-4 years)
Source: BMC Public Health. 2017 Nov 20;17(Suppl 5):854. doi: 10.1186/s12889-017-4860-0 (PMC5753397; doi:10.1186/s12889-017-4860-0)
Supplement: Supplementary file 1 — Search strategies for the systematic review. (DOCX 37 kb) [file 12889_2017_4860_MOESM1_ESM.docx]

**Additional File 1.** Search strategies.

**MEDLINE**

1. Physical Activity.mp.
2. exp Exercise/
3. exp Exercise Movement Techniques/
4. exp Exercise Therapy/
5. Physical Exertion/
6. exp "Physical Education and Training"/
7. exp Sports/
8. (sport$ or bicycl$ or swim$ or walk$ or run$ or jog$).tw,kf.
9. (physical$ adj2 activ$).tw,kf.
10. (aerobic adj2 (train$ or active$)).tw,kf.
11. "Play and Playthings"/ and (activ* or outdoor*).tw,kf.
12. ((activ* or outdoor*) adj3 play*).tw,kf.
13. playground*.tw,kf.
14. active.ti. and (space* or behavio?r* or transport* or commut* or neighbo?rhood* or park* or game* or gaming or lifestyle).mp.
15. (active adj3 (space* or behavio?r* or transport* or commut* or neighbo?rhood* or park* or game* or gaming or lifestyle)).tw,kf.
16. prone position*.mp. or floor time.tw,kf.
17. ((abdomen or stomach or tummy or belly) adj2 (time or play)).tw,kf.
18. or/1-17
19. exp obesity/
20. obes*.tw,kf.
21. exp overweight/
22. (overweight or over-weight).tw,kf.
23. exp Body Fat Distribution/
24. exp body composition/
25. (adipos* or fat).mp.
26. waist.mp.
27. Skinfold Thickness/
28. (skin fold* or skinfold*).tw,kf.
29. (body composition* or BMI or body mass index).tw,kf.
30. exp "body weights and measures"/
31. (bio-impedance analysis or BIA).tw,kf.
32. Absorptiometry, Photon/
33. (absorptiomet* or densitometr* or photodensitometr* or DXA or DEXA).tw,kf.
34. Physical Fitness/
35. (physical conditioning or fitness).tw,kf.
36. Range of Motion, Articular/
37. Postural Balance/
38. Metabolic Syndrome X/
39. Insulin Resistance/
40. ((metabolic adj2 syndrome) or syndrome x).tw,kf. and Risk Factors/
41. exp blood glucose/ or exp diabetes mellitus, type 2/
42. exp glucose intolerance/ or glucose tolerance test/
43. Hemoglobin A, Glycosylated/
44. HbA1c.tw,kf.
45. Bone Density/
46. (bone adj2 (density or mass)).tw,kf.
47. (bone mineral content or bone strength).tw,kf.
48. Bone development/
49. Osteogen*.mp.
50. exp Vitamin D Deficiency/
51. exp Cardiovascular Diseases/
52. ((cardiovascular or heart or vascular) adj2 risk$).tw,kf.
53. exp Hypertension/
54. (hypertens* or high blood pressure).tw,kf.
55. exp blood pressure determination/
56. blood pressure/
57. Hypercholesterolemia/
58. exp Hyperlipidemias/
59. (cholesterol or hypercholester* or hyperlipid* or dyslipid*).tw,kf.
60. exp Plagiocephaly/
61. Plagioceph*.tw,kf.
62. Torticollis/
63. exp Psychomotor Performance/
64. motor development.tw,kf.
65. Motor Activity/
66. Gross motor skill*.tw,kf.
67. (fine motor skill* or locomotor control or object control).tw,kf.
68. exp "Wounds and Injuries"/
69. exp Child Development Disorders/
70. Child Development/
71. Developmental milestone*.mp.
72. gd.fs.
73. Child Behavior Disorders/
74. Child Behavior/
75. exp Adaptation, Psychological/
76. (pro-social behav* or prosocial behav* or social behav*).tw,kf.
77. exp *Social Behavior/
78. ((behavio?ral adj (conduct or disorder*)) or conduct disorder*).tw,kf.
79. exp Aggression/
80. Interpersonal Relations/
81. Attention/
82. Attention Deficit Disorder with Hyperactivity/
83. concentrat*.tw,kf.
84. Cognitive develop*.tw,kf.
85. Achievement/
86. (academic achievement or educational achievement).tw,kf.
87. Educational Achievement/
88. School Admission Criteria/
89. (grade-point average or grade point average or GPA).tw,kf.
90. Educational measurement/
91. Educational status/
92. Language Development Disorders/
93. Language Development/
94. Communication/
95. Speech Perception/
96. Verbal Behavior/
97. Vocabulary/
98. exp Self Concept/
99. (self-esteem or self esteem).tw,kf.
100. Self Efficacy/
101. Self-Control/
102. (self regulation or self control).tw,kf.
103. Executive function/
104. exp Memory/
105. Depression/
106. exp Mood Disorders/
107. exp Anxiety Disorders/
108. Affective Symptoms/
109. Stress, Psychological/
110. "Quality of Life"/
111. Temperament/
112. (depression or depressive).tw,kf.
113. or/19-112
114. 18 and 113
115. limit 114 to ("infant (1 to 23 months)" or "preschool child (2 to 5 years)")
116. 114 and (pre-school* or preschool* or early childhood).tw,kf.
117. 115 or 116
118. limit 117 to (english or french)
119. limit 118 to yr="1860 - 2000"
120. remove duplicates from 119
121. limit 118 to yr="2001 - 2007"
122. remove duplicates from 121
123. limit 118 to yr="2008 - 2016"
124. remove duplicates from 123
125. 120 or 122 or 124
126. limit 125 to journal article
127. (randomized controlled trial.pt. or controlled clinical trial.pt. or randomized.ab. or placebo.ab. or clinical trials as topic.sh. or randomly.ab. or trial.ti.) not (animals/ not humans/)
128. 126 and 127
129. (2016041* or 201605* or 201606* or 201607* or 201608* or 201609* or 20161*).dc.
130. 128 and 129
131. Remove duplicates from 130

**EMBASE**

1. exp physical activity/ or exp exercise/ or exp kinesiotherapy/ or physical education/ or exp sport/
2. sport* or bicycl* or swim* or walk* or run* or jog*).tw,kw.
3. (aerobic adj2 (train$ or active$)).tw,kw.
4. Play/ and (activ* or outdoor*).tw,kw.
5. (((activ* or outdoor*) adj3 play*) or playground*).tw,kw.
6. active.ti. and (space* or behavio?r* or transport* or commut* or neighbo?rhood* or park* or game* or gaming or lifestyle).mp.
7. (active adj3 (space* or behavio?r* or transport* or commut* or neighbo?rhood* or park* or game* or gaming or lifestyle)).tw,kw.
8. (prone position* or floor time).tw,kw.
9. ((abdomen or stomach or tummy or belly) adj2 (time or play)).tw,kw.
10. or/1-9
11. exp obesity/ or exp adipose tissue/ or body composition/ or body fat/ or body fat distribution/ or skinfold thickness/ or exp body weight/ or body mass/
12. (obes* or overweight or over-weight or adipos* or fat or waist or skin fold* or skinfold* or body composition* or bmi or body mass index).tw,kw.
13. dual energy x ray absorptiometry/ or photon absorptiometry/
14. (bio-impediance analysis or bia or absorptiomet* or densitometr* or photodensitometr* or DXA or DEXA).tw,kw.
15. fitness/ or "range of motion"/ or body equilibrium/
16. (physical conditioning or fitness).tw,kw.
17. metabolic syndrome X/ or insulin resistance/ or non insulin dependent diabetes mellitus/ or glucose blood level/ or glucose intolerance/ or exp glucose tolerance test/ or hemoglobin A1c/
18. ((metabolic adj2 syndrome) or syndrome x).tw,kw. and Risk Factor/
19. HbA1c.tw,kw.
20. bone density/ or bone development/ or (bone adj2 (density or mass)).tw,kw. or (bone mineral content or bone strength).tw,kw. or osteogen*.mp.
21. vitamin d deficiency/ or exp cardiovascular disease/ or ((cardiovascular or heart or vascular) adj2 risk*).tw,kw.
22. exp hypertension/ or exp blood pressure measurement/ or exp blood pressure/ or hyperlipidemia/ or exp hypertriglyceridemia/
23. (hypertens* or high blood pressure or cholesterol or hypercholester* or hyperlipid* or dyslipid*).tw,kw.
24. Plagiocephaly/ or plagiocephal*.tw,kw.
25. torticollis/
26. exp psychomotor performance/
27. exp motor activity/
28. torticollis/ or exp motor activity/ or (motor development or gross motor skill* or fine motor skill* or locomotor control or object control).tw,kw.
29. exp wound/
30. exp developmental disorder/ or exp child development/ or developmental milestone.tw,kw.
31. exp behavior disorder/ or child behavior/ or adaptive behavior/ or exp aggression/ or exp *social behavior/ or human relation/
32. (prosocial behav* or social behav* or ((behavio?ral adj (conduct or disorder*)) or conduct disorder*)).tw,kw.
33. attention deficit disorder/ or exp attention/ or cognitive development/ or achievement/ or exp academic achievement/ or educational status/
34. (concentrat* or academic achievement or educational achievement or grade-point average or grade point average or GPA).tw,kw.
35. exp developmental language disorder/
36. language development/
37. exp interpersonal communication
38. speech perception/
39. exp verbal behavior/
40. exp developmental language disorder/ or language development/ or exp interpersonal communication/ or speech perception/ or exp verbal behavior/
41. exp self concept/
42. (self esteem or self esteem or self regulation or self control).tw,kw.
43. executive function/ or exp memory/ or exp mood disorder/ or exp anxiety disorder/ or emotional disorder/ or mental stress/ or exp "quality of life"/ or exp temperament/
44. (depression or depressive).tw,kw.
45. or/11-44
46. 10 and 45
47. limit 46 to (infant <to one year> or preschool child <1 to 6 years>)
48. 46 and (pre-school* or preschool* or early childhood).tw,kw.
49. 47 or 48
50. limit 49 to yr="1902 - 2005"
51. 49 not 50
52. remove duplicates from 50
53. remove duplicates from 51
54. 52 or 53
55. limit 54 to (english or french)
56. 55 and (Randomized Controlled Trial/ or Single-blind Procedure/ or Crossover Procedure/ or Double-blind Procedure/ or random$.tw. or factorial$.tw. or crossover$.tw. or cross over$.tw. or cross-over$.tw. or placebo$.tw. or (doubl$ adj blind$).tw. or (singl$ adj blind$).tw. or assign$.tw. or allocat$.tw. or volunteer$.tw.)
57. limit 56 to dd=20160401-20161110
58. remove duplicates from 57
59. limit 58 to embase

**PsycINFO**

1. physical activity/ or exp exercise/ or activity level/ or movement therapy/ or dance therapy/ or mind body therapy/ or energy expenditure/ or physical education/ or exp sports/
2. (sport* or bicycl* or swim* or walk* or run* or jog*).tw,id.
3. (physical* adj2 activ*).tw,id.
4. (aerobic adj2 (train* or active*)).tw,id.
5. (childhood play behavior/ or childhood play development/ or games/ or recreation/) and (activ* or outdoor*).tw,id.
6. ((activ* or outdoor*) adj3 play*).tw,id.
7. playgrounds/ or playground*.tw,id.
8. active.ti. and (space* or behavio?r* or transport* or commut* or neighbo?rhood* or park* or game* or gaming or lifestyle).tw,id.
9. (active adj3 (space* or behavio?r* or transport* or commut* or neighbo?rhood* or park* or game* or gaming or lifestyle)).tw,id.
10. (prone position* or floor time).tw,id.
11. ((abdomen or stomach or tummy or belly) adj2 (time or play)).tw,id.
12. or/1-11
13. exp overweight/ or body weight/ or weight gain/ or weight loss/ or body fat/ or body mass index/ or weight control/ or body size/
14. (obes* or overweight or over-weight or adipos* or fat or waist or skinfold* or skin fold* or body composition or bmi or body mass index).tw,id.
15. (bio-impedance analysis or BIA or absorptiomet* or densitometr* or photodensitometr* or DXA or DEXA).tw,id.
16. physical fitness/ or physical endurance/ or physical strength/ or "range of motion"/
17. (physical conditioning or fitness).tw,id.
18. metabolic syndrome/ or insulin/ or blood suger/ or type 2 diabetes/ or glucose metabolism/ or glucose/
19. ((metabolic adj2 syndrome) or syndrome x).tw,id. and Risk Factors/
20. (glycosylated hemoglobin A or HbA1c).tw,id.
21. (bone adj2 (density or mass or development)).tw,id.
22. (bone mineral content or bone strength or osteogen*).tw,id.
23. bones/
24. exp vitamin deficiency disorders/
25. exp Cardiovascular Disorders/ or exp hypertension/ or cholesterol/ or lipids/
26. ((cardiovascular or heart or vascular) adj2 risk*).tw,id.
27. (hypertens* or high blood pressure or cholesterol or hypercholester* or hyperlipid* or dyslipid*).tw,id.
28. torticollis/
29. Plagioceph*.tw,id.
30. exp motor development/ or exp motor processes/
31. (motor development or motor skill* or locomotor control or object control).tw,id.
32. injuries/ or exp head injuries/ or exp spinal cord injuries/ or exp wounds/
33. developmental disabilities/ or exp delayed development/ or exp intellectual development disorder/
34. developmental milestone*.tw,id.
35. behavior disorders/ or exp aggressive behavior/ or exp antisocial behavior/ or exp behavior problems/
36. exp social behavior/
37. interpersonal relationships/ or exp interpersonal interaction/
38. ((behavio?ral adj (conduct or disorder*)) or conduct disorder* or prosocial behav* or social behav*).tw,id.
39. exp attention/ or exp attention deficit disorder/ or concentration/ or distraction/ or concentrat*.tw,id.
40. exp cognitive development/
41. exp academic achievement/ or academic achievement prediction/ or academic aptitude/ or academic failure/ or educational attainment level/ or achievement/ or student admission criteria/ or educational measurement/
42. (cognitive develop* or academic achievement or educational achievement or grade-point average or grade point average or GPA).tw,id.
43. exp Language Development/ or exp Language Disorders/ or exp communication/ or speech perception/ or vocabulary/
44. self-perception/ or exp self-concept/ or self-efficacy/ or self-esteem/ or self-control/ or anger control/ or exp emotional control/ or emotional regulation/ or exp impulse control disorders/ or self-regulation/ (117472)
45. (self-esteem or self esteem or self regulation or self control).tw,id.
46. exp executive function/ or exp cognitive ability/ or exp memory/ or exp affective disorders/ or exp anxiety disorders/ or psychological stress/ or "quality of life"/ or exp Life Satisfaction/ or exp Well Being/ or personality/
47. (depression or depressive).tw,id.
48. or/13-47
49. 12 and 48
50. limit 12 to (2330 motor processes or 2340 cognitive processes or 2343 learning & memory or 2346 attention or 2800 developmental psychology or 2820 cognitive & perceptual development or 2840 psychosocial & personality development or 2900 social processes & social issues or 3020 group & interpersonal processes or 3040 social perception & cognition or 3211 affective disorders or 3230 behavior disorders & antisocial behavior or 3250 developmental disorders & autism or 3295 cardiovascular disorders)
51. 49 or 50
52. limit 51 to (140 infancy <2 to 23 mo> or 160 preschool age <age 2 to 5 yrs>)
53. (pre-school* or preschool* or early childhood).mp. [mp=title, abstract, heading word, table of contents, key concepts, original title, tests & measures]
54. 51 and 53
55. 52 or 54
56. Clinical Trials/ OR Treatment Effectiveness Evaluation/ or Placebo/ or Followup Studies/ or (placebo* OR random* OR comparative stud*).ti,ab. OR (clinical ADJ3 trial*).ti,ab. OR (research ADJ3 design) .ti,ab.OR (evaluat* ADJ3 stud*).ti,ab. OR (prospectiv* ADJ3 stud*).ti,ab. OR ((singl* OR doubl* OR trebl* OR tripl*) ADJ3 (blind* OR mask*)).ti,ab.
57. 55 and 56
58. 57 and (201604* or 201605* or 201606* or 201607* or 291608* or 201609* or 20161*).up.
59. remove duplicates from 58

**CENTRAL**

1. (health* or wellness or well being or wellbeing or quality of life or social behav* or prosocial behav* or behavio* conduct or conduct disorder* or aggression or delinquen* or cognitive dev* or academic achievement or educational achievement or grade point average or GPA or drop out or dropout or language dev* or verbal or vocabulary or self control or self regulation or self efficacy or executive function or memory or attention or concentrat* or psychomotor performance or motor development or motor skill* or motor activit* or movement or physical competenc* or physical* litera* or postural balance or psychosocial or psycho social or interpersonal or depression or depressive or depressed or mood disorder* or anxiety or distress or worry or psychological stress* or burnout or burn out or self esteem or self concept or (bone adj2 density) or (bone adj2 mass) or absorptiomet* or densitometr* or photodensitometr* or DXA or DEXA or hypertens* or high blood pressure or cholesterol or hypercholester* or hyperlipid* or dyslipid* or lipids* or lipoprotie* or obese or obesity or overweight or body mass index or BMI or waist or adipos* or fat or body composition or skin fold* or skinfold* or metabolic syndrome or insulin resistance or diabet* or glucose or HBA1C or fitness or physical condition* or cardiovascular or placioceph* or torticollis or child develop* or developmental milestone* or injur*).mp.
2. (((((exercise or (physical* adj2 activ*) or aerobic) adj2 train*) or aerobic) adj2 active*) or sport* or outdoor* or playground or play or playing or (rough adj2 tumble) or active recreation* or run* or walk* or jog* or bicycl* or biking or cycling or tricylc* or tummy time or floor time or prone position or crawl* or swim* or soccer or gymnastic*).mp.
3. (Infan* or pre-school* or preschool* or early childhood or child*).mp.
4. 1 and 2 and 3
5. limit 4 to medline records
6. limit 4 to embase records
7. 4 not (5 or 6)
8. 6 and (201604* or 201605* or 201606* or 201607* or 291608* or 201609* or 20161*).up.

**Pubmed**

((physical activity ) and (Infant or child* or preschool or pre-school) and (pubstatusaheadofprint or publisher[sb] or pubmednotmedline[sb]))

**SPORTdiscus**

| S7 | S6 with Limiters - Published Date: 20150101-20171231; Language: English, French; Publication Type: Academic Journal; Document Type: Article |
| --- | --- |
| S6 | S4 and S5 |
| S5 | random* OR RCT OR placebo* OR comparative stud* OR (clinical N3 trial*) OR (prospectiv* N3 stud*) OR (singl* N3 blind*) OR (singl* N3 mask*) OR (doubl* N3 blind*) OR (doubl* N3 mask*) OR (trebl* N3 blind*) OR (trebl* N3 mask*) OR (tripl* N3 blind*) OR (tripl* N3 mask*) OR crossover* OR cross over* OR cross-over |
| S4 | S1 AND S2 AND S3 |
| S3 | TI ( health* OR wellness OR well being OR wellbeing OR “quality of life” OR social behav* OR prosocial behav* OR behavio* conduct or conduct disorder* OR aggression OR delinquen* OR cognitive dev* OR academic achievement OR educational achievement OR grade point average OR GPA OR drop out OR dropout OR language dev* OR verbal or vocabulary OR self control or self regulation or self efficacy OR executive function OR memory OR attention or concentrat* OR psychomotor performance OR motor development OR motor skill* OR motor activit* OR movement OR physical competenc* OR physical* litera* OR postural balance OR psychosocial OR psycho social OR interpersonal OR depression OR depressive OR depressed OR mood disorder* or anxiety OR distress OR worry OR psychological stress* OR burnout OR burn out OR self esteem OR self concept OR (bone N2 density) OR (bone N2 mass) OR absorptiomet* or densitometr* or photodensitometr* or DXA or DEXA OR hypertens* OR high blood pressure OR cholesterol OR hypercholester* OR hyperlipid* OR dyslipid* OR lipids* OR lipoprotie* OR obese OR obesity OR overweight OR body mass index OR BMI OR waist OR adipos* OR fat OR body composition OR skin fold* or skinfold* OR metabolic syndrome OR insulin resistance OR diabet* OR glucose OR HBA1C OR fitness or physical condition* OR cardiovascular OR placioceph* OR torticollis OR child develop* or developmental milestone* or injur*) ) OR SU (health* OR wellness OR well being OR wellbeing OR “quality of life” OR social behav* OR prosocial behav* OR behavio* conduct or conduct disorder* OR aggression OR delinquen* OR cognitive dev* OR academic achievement OR educational achievement OR grade point average OR GPA OR drop out OR dropout OR language dev* OR verbal or vocabulary OR self control or self regulation or self efficacy OR executive function OR memory OR attention or concentrat* OR psychomotor performance OR motor development OR motor skill* OR motor activit* OR movement OR physical competenc* OR physical* litera* OR postural balance OR psychosocial OR psycho social OR interpersonal OR depression OR depressive OR depressed OR mood disorder* or anxiety OR distress OR worry OR psychological stress* OR burnout OR burn out OR self esteem OR self concept OR (bone N2 density) OR (bone N2 mass) OR absorptiomet* or densitometr* or photodensitometr* or DXA or DEXA OR hypertens* OR high blood pressure OR cholesterol OR hypercholester* OR hyperlipid* OR dyslipid* OR lipids* OR lipoprotie* OR obese OR obesity OR overweight OR body mass index OR BMI OR waist OR adipos* OR fat OR body composition OR skin fold* or skinfold* OR metabolic syndrome OR insulin resistance OR diabet* OR glucose OR HBA1C OR fitness or physical condition* OR cardiovascular OR placioceph* OR torticollis OR child develop* or developmental milestone* or injur*) |
| S2 | TI ( exercise or (physical* n2 activ*) OR aerobic N2 train* or aerobic N2 active* OR sport* or outdoor* OR playground or play or playing OR "rough and tumble" or "active recreation*" OR run* OR walk* OR jog* OR bicycl* or biking or cycling or tricylc* or "tummy time" or "floor time" or prone position or crawl* OR swim* or soccer or gymnastic* ) OR SU (exercise or (physical* n2 activ*) OR aerobic N2 train* or aerobic N2 active* OR sport* or outdoor* OR playground or play or playing OR "rough and tumble" or "active recreation*" OR run* OR walk* OR jog* OR bicycl* or biking or cycling or tricylc* or "tummy time" or "floor time" or prone position or crawl* OR swim* or soccer or gymnastic* ) |
| S1 | Infan* or pre-school* or preschool* or early childhood or SU child* |
