# Supplementary material for: Systematic review of the relationships between physical activity and health indicators in the early years (0-4 years)
Source: BMC Public Health. 2017 Nov 20;17(Suppl 5):854. doi: 10.1186/s12889-017-4860-0 (PMC5753397; doi:10.1186/s12889-017-4860-0)
Supplement: Supplementary file 2 — Supplementary Tables S1-S8. Summary of studies included in the systematic review for each health indicator sorted by (whenever possible) study design, age group, and physical activity measurement. (DOCX 177 kb) [file 12889_2017_4860_MOESM2_ESM.docx]

**Additional File 2.** Supplementary tables

**Table S1**. Summary of included studies for adiposity

| Refid #. Author (year); country | Study Design | Sample | Exposure | Outcome | Main Findings |
| --- | --- | --- | --- | --- | --- |
| De Vries et al. 2015 [1]; Netherlands | RCT | n=161; age (baseline): 2 wk. | Intervention group: Parents received recommendations from a nurse in regards to a physical activity stimulation program at ages 2 wk, 2, 4, 8, 11 mo.  Control group: Standard care with no activity recommendations. | Overweight/obese (>85^th^ percentile) and BMI (kg/m^2^) assessed via objectively measured height and weight at age 2.5 yr.  Waist circumference (cm), hip circumference (cm), and sum of 4 skinfolds (triceps, biceps, subscapular, supra-ilacal; mm) objectively measured at age 2.5 yr.  Body fat % (weight- fast free mass) objectively measured using bioelectrical impedance analyses at age 2.5 yr. | The mean sum of four skinfolds was significantly lower in the intervention (29.6±4.7) compared to the control group (32.4±6.0) at age 2.5 yr.  There were no significant differences in percentage overweight (intervention: 12±13.5; control: 8±14.8), BMI (intervention: 16.2±1.1; control: 16.4 ± 1.2), waist circumference (intervention: 48.8±2.3; control: 49.2±2.9), hip circumference (intervention: 50.2±2.6; control: 50.9±2.9), or body fat % (intervention: 14.1±3.4; control: 15.2±4.4) between intervention and control groups at age 2.5 yr.  Note: There were no significant differences in accelerometer-derived TPA (cpm) between intervention and control group at age 2.5 yr. |
| Bonvin et al. 2013 [2]; Switzerland | Clustered RCT | n=1467; mean age: 3.3 yr. | Intervention: Government-led physical activity program (no specific time component or curriculum) for 10 mo.  Control: Regular program. | Normal weight and overweight/obese (IOTF) and BMI (kg/m2) assessed via objectively measured height and weight at baseline and 10 mo follow-up. | No significant difference in BMI was observed between the mean individual changes in the intervention group (baseline: 16.3±1.4; follow-up: 16.1±1.3) and the control group (baseline: 16.2±1.2; follow-up: 16.2±1.3).  No significant difference in % overweight was observed between the intervention group (baseline: 15.7%; follow-up: 17.5%) and the control group (baseline: 11%; follow-up: 14.1%; OR=0.74, 95% CI: 0.48, 2.76).  Note: No significant difference in TPA (cpm) were observed between the mean individual changes in the intervention group (baseline: 620 ±278; follow-up: 765±340) and the control group (baseline: 600 ±206; follow-up: 711±219). No significant differences in MVPA (epochs/hr) were observed between the mean individual changes in the intervention group (baseline: 29.2±14; follow-up: 37.2±17.1) and the control group (baseline: 28.1±12.5; follow-up: 35.9±13.7). No significant differences in VPA (epochs/hr) were observed between the mean individual changes in the intervention group (baseline: 8.1±6.1; follow-up: 10.3±7.5) and the control group (baseline: 7.4±5.3; follow-up: 9.2±6.2). |
| Jones et al. 2011 [3]; Australia | Clustered RCT | n=97; mean age (baseline): 4.13 yr. | Intervention: Structured activities  designed to improve motor  skills - 3 times/wk for  20 wk in childcare.  Control: Usual  Care. | BMI (kg/m^2^) assessed via objectively measured height and weight at baseline and 6 mo follow-up. | No significant difference in the 6 mo changes in BMI were observed between intervention and control groups (adjusted mean difference=-0.8; 95%CI: -0.33, 0.17, p=0.53).  *Note:* Change in TPA from baseline to the last two wk of the intervention assessed via counts/min was significantly higher in the intervention compared to the control group (adjusted mean difference=110.48, p=0.01). |
| Annesi et al. 2013 [4]; United States | Clustered RCT | n=1154; mean age (baseline): 4.4 yr. | Intervention: Pre-school teacher administered 30 min of daily structured physical activity plus cognitive-behavioral training and resources for 9 mo.  Control: Pre-school teacher administered 30 min of daily structured physical activity for 9 mo. | BMI (kg/m^2^) assessed via height and weight at baseline, 1, 5, 9 mo follow-up.  Note: Unclear if objectively or subjectively measured. | Over the 9 mo, a significantly larger reduction in BMI was observed in the intervention group (Mo 1: 16.31±1.64; Mo 9: 16.17±1.52) compared to the control group (Mo 1: 16.27±1.76; Mo 9: 16.23±1.87). A significant with-in group reduction in BMI was only observed in the intervention group (p<0.001).  Note: Mean accelerometer-derived MVPA (% of time) and VPA (% of time) across 1, 5, 9 mo follow-up measurements was significantly higher in the intervention compared to the control group (MVPA: 23.9 vs. 22.5 %; VPA: 17.5 vs. 15.7 %). |
| Mo-suwan et al. 1998 [5]; Thailand | Clustered RCT | n=310; mean age: 4.5 yr. | Intervention: 15 min walk in the morning and 20 min aerobic dance session in the afternoon - 3 times/wk for 29.3 to 30 wk. This was in addition to routine school physical activities.  Control: Routine school physical activities. | BMI (kg/m^2^) assessed via objectively measured height and weight in June (baseline), September, November, and February.  Weight/height (kg/m^3^) and tricep skinfold thickness (mm) objectively measured in June (baseline), September, November, and February. | No significant differences in the final: BMI (intervention: 15.76±2.46; control: 15.94±2.26), weight/height (intervention: 14.19±1.89; control: 14.43±1.98), or tricep skinfold thickness (intervention: 10.3±3.9; control: 10.4±4.1) were observed between intervention and control groups.  No significant differences in the slope of: BMI (intervention: 20.63±1.04^3^; control: 20.54±1.00), weight/height (intervention: 21.61±1.00; control: 21.56±0.94), or tricep skinfold thickness (intervention: 0.48±2.25; control: 0.18±2.32) were observed between intervention and control groups.  Note: It is unknown if the intervention resulted in a significant change in physical activity. |
| Monsalves-Alvarez et al. 2015 [6]; Chile | Non-randomized Intervention | n=81; mean age: 4.0 yr. | Intervention: Physical activity classes: 45 min total (15 min sessions) 3 times/wk over 6 mo. | BMI (kg/m^2^) and BMI z-score (criteria not stated) assessed via objectively measured height and weight at baseline and 6 mo follow-up. | *Boys:* There was no significant difference in BMI (baseline: 16.7±1.5; follow-up: 16.8±1.5 or BMI z-score (baseline: 1.09±0.8; follow-up: 1.00±0.8) from baseline to 6 mo follow-up.  *Girls:* There was no significant difference in BMI (baseline: 16.6±2; follow-up: 16.5±1.9) or BMI z-score (baseline: 0.82±1.2; follow-up: 0.79±1.1) from baseline to 6 mo follow-up.  Note: It is unknown if the intervention resulted in a significant change in physical activity. |
| Krombholz, 2012 [7]; Germany | Non-randomized Intervention | n=559; mean age (baseline): intervention group, 55.1 mo; control group, 54.4 mo. | Intervention: Physical education and structured physical activity session: 45 min session at least 1 times/wk. Physical activity sessions: 20 min on other days for 20 mo.  Control: Usual curriculum. Included physical education and structured physical activity session: 45 min session 1 time/wk. | BMI (kg/m^2^) assessed via objectively measured height and weight at baseline, 11 mo follow-up and 20 mo follow-up.  Skinfold thickness (abdomen, scapula and triceps; mm) objectively measured at baseline, 11 mo follow-up and 20 mo follow-up. | The intervention and control group did not significantly differ in change in BMI over the three measurement periods of baseline (intervention: 15.78±1.59; control: 15.56±1.27), 11 mo follow-up (intervention: 15.91±1.80; control: 15.58±1.42), and 20 mo follow-up (intervention: 15.89±1.95; control: 15.70±1.58).  The intervention and control group did not significantly differ in change in skinfold thicknesses over the three measurement periods of baseline (intervention: 4.79±1.91; control: 4.39±1.42), 11 mo follow-up (intervention: 5.09±2.40; control: 4.56±2.05), and 20 mo follow-up (intervention: 5.85±3.16; control: 5.45±2.68).  Note: It is unknown if the intervention resulted in a significant change in physical activity. |
| Sijtsma et al. 2013 [8]; Netherlands | Cross-sectional and Longitudinal | n (cross-sectional) =1722  n (longitudinal) =1283; mean age (baseline): 41 wk. | Unrestricted moving time (hr/day) assessed via proxy-report questionnaire at baseline at age 9 mo. | Weight-for-height z-score and waist- circumference-for- age z-score (sample-specific) objectively measured at ages 9 mo and 24 mo. | *Cross-sectional:* No significant differences were observed in weight-for-height z-score (<5hr/day: 0.04±0.97; ≥5 hr/day: 0.06±1.01) or waist circumference-for-age z-score (<5hr/day: 0.04±0.99; ≥5 hr/day: −0.05±1.01) at age 9 mo by unrestricted moving time groups at age 9 mo.  *Longitudinal:* No significant differences were observed in weight-for-height z-score (<5hr/day: 0.04±0.98; ≥5 hr/day: −0.02±0.99) or waist circumference-for-age z-score (<5hr/day: 0.03±0.97; ≥5 hr/day: 0.00±1.01) at age 24 mo by unrestricted moving time groups at age 9 mo.  Significant differences were observed in the changes of weight-for-height z-score (<5hr/day: 0.04±0.80; ≥5 hr/day: −0.11±0.70) but not waist circumference-for-age z-score (<5hr/day: −0.02±1.14 ≥5 hr/day: 0.01±1.13) from age 9 to 24 mo by unrestricted moving time groups at age 9 mo. |
| Carter et al. 2011 [9]; New Zealand | Cross-sectional and Longitudinal | n=244; age (baseline): 3 yr. | Accelerometer-derived TPA (cpm) assessed at ages 3, 4, 5 yr. | BMI (kg/m^2^) assessed via objectively measured height and weight at ages 3, 4, 5, 6, 7 yr.  Fat mass index (fat mass (kg)/height (m)^2^) and fat free mass index (fat free mass (kg)/height (m)^2^) objectively measured using dual energy x ray absorptiometry (DXA) at ages 5 and 7 yr. | *Cross-sectional:* TPA at ages 3, 4, 5 yr was not significantly associated with BMI at age 3, 4, 5 yr (β=−0.02; 95%CI: −0.24, 0.20).  *Longitudinal:* TPA averaged over ages 3, 4, 5 yr was not significantly associated with BMI (β=0.12; 95%CI: −0.11, 0.34) fat mass index (β= −0.12; 95%CI: −0.39, 0.15) and fat free mass index (β= −0.04; 95%CI: −0.16, 0.09) at age 7 yr. |
| DuRant et al. 1994 [10]; United States | Longitudinal | n=123; mean age (baseline): 4.04 yr. | Physical activity objectively measured with the Children’s Activity Rating Scale between baseline and 1 yr follow-up. | Waist to hip ratio objectively measured at baseline and 1 yr follow-up. | Mean activity level was significantly correlated with waist to hip ratio (time point unknown; r = −0.24; p ≤0.02). |
| Klesges et al. 1995 [11]; United States | Longitudinal | n=146; mean age (baseline): 4.4 yr. | Structured, leisure, and aerobic activity (much less, about the same, much more than others) assessed via proxy-report questionnaire at baseline, 1 yr follow-up and 2 yr follow-up. | BMI (kg/m^2^) assessed via objectively measured height and weight at baseline, 1 yr follow-up and 2 yr follow-up. | Baseline aerobic activity (but not structured or leisure; data not shown) was significantly associated with change in BMI over the 2 yr follow-up (β= −0.319; p=0.0333).  Change in structured, leisure, and aerobic activity from baseline to 1 yr follow-up and from 1 yr to 2 yr follow-up (β = −0.319; p=0.0867) was not significantly associated with change in BMI over the 2 yr follow-up (other data not shown). |
| Butte et al. 2016 [12]; United States | Cross-sectional and Longitudinal | n=119; mean age: 4.6 yr. | Accelerometer-derived TPA (counts x 10^4^/day), MVPA (min/day) at baseline.  Activity energy expenditure objectively measured with doubly labeled water at baseline. | BMI (kg/m^2^) assessed via objectively measured height and weight at baseline and 1-yr follow-up.  Fat free mass (kg), fat mass (kg) percent fat mass (%) objectively measured using dual energy X-ray absorptiometry at baseline and 1-yr follow-up. | *Cross-sectional:* MVPA was significantly associated with percent fat mass (β= −0.006; SE=0.026) but not BMI (β= −0.005; SE=0.007), fat free mass (β=0.011; SE=0.009), fat mass (β= −0.015; SE=0.008).  Activity energy expenditure was significantly associated with BMI (β=0.003; SE=0.002) and fat free mass (β=0.004; SE=0.001) but not fat mass (β= 0.003; SE=0.002) or percent fat mass (β= 0.001; SE=0.005).  No significant associations were observed between TPA and BMI (β= −0.005; SE=0.007), fat free mass (β= −0.011; SE=0.009), fat mass (β= −0.008; SE=0.008), and percent fat mass (β= −0.001; SE=0.028).  *Longitudinal:* MVPA was significantly associated with 1-yr changes in fat free mass (β=0.008; SE=0.003) but not BMI (β=0.007; SE=0.003), fat mass (β=0.0004; SE=0.003), or percent fat mass (β= −0.001; SE=0.013).  Activity energy expenditure was significantly associated with 1-year changes in BMI (β=0.002; SE=0.001); fat free mass (β=0.002; SE=0.001), fat mass (β=0.001; SE=0.001) but not percent fat mass (β= 0.001; SE=0.002).  TPA was not significantly associated with 1-yr changes in BMI (β=0.006; SE=0.003), fat free mass (β=0.007; SE=0.004), fat mass (β= -0.003; SE=0.004), and percent fat mass (β= -0.018; SE=0.010). |
| Huynh et al. 2011 [13]; Vietnam | Longitudinal | n=526; mean age (baseline): 56.3 mo. | VPA (hr/day) assessed via proxy-report questionnaire at baseline, 6 mo follow-up, 12 mo follow-up. | BMI (kg/m^2^) assessed via objectively measured height and weight at baseline, 6 mo follow-up, 12 mo follow-up.  Sum of skinfold thicknesses (triceps, subscapular, suprailiac; mm) at baseline, 6 mo follow-up, 12 mo follow-up. | *Boys:* VPA was not significantly associated with change in BMI (β=0.11; 95%CI: 0.29, 0.07) or change in sum of skinfolds (β=0.01; 95%CI: 0.37, 0.39).  *Girls:* VPA was not significantly associated with change in BMI (β=0.001; 95%CI: 0.06, 0.06) or change in sum of skinfolds (β=0.38; 95%CI: 0.77, 0.01). |
| De Coen et al. 2014 [14]; Belgium | Longitudinal | n=568; mean age (baseline): 4.95 yr. | Physical activity at home (hr/wk) and structured physical activity (sports club, after school sports; hr/wk) assessed via proxy-report questionnaire at baseline. | Non-overweight and overweight (Flemish reference data; ≥1 SD) assessed via objectively measured height and weight (BMI) at baseline, 18 mo follow-up and 30 mo follow-up. | ≤6 hr/wk of physical activity at home compared to >6 hr/wk was not significantly associated with an increased likelihood of being overweight at 18 mo follow-up (OR=1.10; 95%CI: 0.66, 1.84) or 30 mo follow-up (OR=0.82; 95%CI: 0.49, 1.38).  ≤4 hr/wk of structured physical activity compared to >4 hr/wk was not significantly associated with an increased likelihood of being overweight at 18 mo follow-up (OR=0.78; 95%CI: 0.20, 3.01) or 30 mo follow-up (OR=1.03; 95%CI: 0.38, 2.76). |
| He et al. 2000 [15]; China | Case-control | n=374; age range: 0.1-2.9 yr. | Outdoor activity (hr/day) assessed via proxy-report questionnaire. | Cases (obese; weight that exceeded the standard weight  for height, age and sex by more than 20% or,  a height-adjusted weight over 120% of  the National Center for Health Statistics mean) and controls (non-obese) assessed via objectively measured height and weight (BMI). | Outdoor activity ≥ 2hr/day compared to <2 hr/day was not significantly associated with the likelihood of being in the case group (OR=1.13; 95%CI: 0.63, 2.06; p=0.79). |
| Takahashi et al. 1999 [16]; Japan | Case-control | n=1281; age 3 yr. | Physical activity (active, not-active) and outdoor play (hr/day) assessed via proxy report questionnaire. | Non-obese (controls) and obese (cases; BMI >18) assessed via objectively measured height and weight (BMI). | Participants who were not-active compared to those that were active were not significantly more likely to be obese (OR=1.76; p≥0.05).  Participants who engaged in <1 hr/day in outdoor play compared ≥1 hr/day were significantly more likely to be obese (OR=1.38; 95%CI: 1.11, 1.72). |
| Kain & Andrade 1999 [17]; Chile | Case-control | n=616; age :4 yr. | MPA (1.8-3.0 METs; hr/day) and intense physical activity (>3.0 METs; hr/day) assessed via proxy-report questionnaire. | Cases (obese; WHO); controls (non-obese) assessed via objectively measured height and weight (BMI). | Boys: No significant differences were observed in MPA (Obese: 3.2; Non-obese: 2.8) and intense physical activity (Obese: 1.9; Non-obese: 2.0) between obese and non-obese groups.  *Girls:* No significant differences were observed in MPA (Obese: 3.7; non-obese: 3.6) and intense physical activity (Obese: 1.1, non-obese: 1.2) between obese and non-obese groups. |
| Shapiro et al. 1984 [18]; United States | Cross-sectional* | n=170; age (baseline): 6 mo. | TPA assessed via proxy-report log at ages 6 mo and 1, 2, 3, 4 yr. | Sum of skinfolds (triceps, subscapular, suprailiac, and chest) objectively measured at ages 6 mo and at 1, 2, 3, 4 yr. | TPA was significantly correlated with sum of skinfolds at age 6 mo (r=-0.11; p<0.01). Significant correlations were not observed at ages 1 (r=-0.04), 2 (r=-0.08), 3 (r=-0.1), and 4 (r=-0.06) yr (p≥0.05). |
| LaRowe et al. 2010 [19]; United States | Cross-sectional | n=135; age range: 2-5 yr. | Accelerometer-derived of LPA (hr/day) and MVPA (min/day). | Non-overweight and overweight (CDC; ≥ 85^th^ percentile) assessed via objectively measured height and weight (BMI). | LPA did not significantly differ between non-overweight (5.0±0.16) and overweight groups (5.00±0.22; p=0.94).  MVPA did not significantly differ between non-overweight (15.9±1.41) and overweight groups (18.1±0.23; p=0.95). |
| Chen et al. 2011 [20]; United States | Cross-sectional | n=2036; age range: 2-<6 yr. | Physical activity (hr/wk) assessed via proxy-report interview. | Normal weight (<85^th^ percentile), overweight (85^th^ to <95^th^), obese (≥ 95^th^ percentile) assessed via objectively measured height and weight (BMI). | Participants who engaged in ≥7 hr/wk compared to <7 hr/wk of physical activity were significantly less likely to be overweight (OR=0.64; 95%CI: 0.48, 0.85) and obese (OR=0.64; 95%CI: 0.45. 0.90). |
| Johansson et al. 2015 [21]; Sweden | Cross-sectional | n=120; mean age: 2.03 yr. | Accelerometer-derived TPA (CPM for vertical axis and vector magnitude), TPA (steps/day), low-intensity physical activity (min/day), high-intensity physical activity (min/day), 5 min bouts of low-intensity physical activity (min and frequency) and high-intensity physical activity (min and frequency). | Normal weight and overweight (IOTF) assessed via objectively measured height and weight (BMI). | There were no significant differences between normal weight and overweight groups in TPA (vertical: normal weight= 1817±374 vs. overweight= 1841±506, p=0.82; vector magnitude: normal weight= 3041±526 vs. overweight= 3163±488, p=0.38; steps: normal weight= 11175±1649 vs. overweight= 11251±1422, p=0.86), low-intensity physical activity (min: normal weight= 265±34 vs. overweight= 370±36, p=0.59; no of 5 min bouts: normal weight= 2.2±1.5 vs. overweight= 1.9 ± 1.4, p=0.44; total time in 5 min bouts: normal weight= 12±9 vs. overweight= 10±8, p=0.40), and high-intensity physical activity (min: normal weight= 84±23 vs. overweight= 89±23, p=0.83; no of 5 min bouts: normal weight= 0.2±0.3 vs. overweight= 0.2±0.3, p=0.93; total time in 5 min bouts: normal weight= 2.5±14.0 vs. overweight= 1.0±1.6, p=0.68). |
| Nelson et al. 2006 [22]; United States | Cross-sectional | n=316; age range: 2-4 yr. | Active play/exercise (min/day) assessed via proxy-report questionnaire. | At risk for overweight/ overweight (CDC; ≥85^th^ percentile) assessed via proxy-reported height and weight (BMI). | Children who participated in <30 min/day compared to ≥30 min/day of active play/exercise were significantly more likely to be at risk for overweight/overweight (OR=1.80; 95%CI: 1.10, 2.94). |
| Østbye et al. 2013 [23]; United States | Cross-sectional | n=208; age range: 2-5 yr. | Accelerometer-derived MVPA (min/day) | Healthy weight and overweight (CDC; ≥85^th^ percentile) assessed via height and weight (BMI).  Note: Unclear if objectively or subjectively measured. | No significant difference in MVPA were observed between healthy weight (18.0±1.09) and overweight groups (15.8±1.57; p≥0.05). |
| Kagamimori et al. 1999 [24]; Japan | Cross-sectional | n=8834; age: 3 yr. | Physical activity (active, moderately active, inactive) and outdoor play (hr/day) assessed via proxy report questionnaire. | Non-obese (Kaup index <18) and obesity (Kaup index ≥ 18) assessed via objectively measured height and weight (BMI). | A significantly higher proportion of children in the obese group (49.6%) were physical inactive compared to the non-obese group (44.9%; p<0.05).  No significant differences in infrequent outdoor play (<1 hr/day) were observed between the obese (59.1%) and non-obese groups (58.1%; p≥0.05). |
| Trost et al. 2003 [25]; United States | Cross-sectional | n=245; age range: 3-5 yr. | Mean activity rating and % time in MVPA at preschool assessed via direct observation using OSRAP.  Accelerometer-derived total counts, MVPA (intervals), and VPA (intervals) at preschool. | Overweight (CDC; ≥85 percentile) assessed via objectively measured height and weight (BMI). | *Boys:* Overweight boys had significantly lower mean physical activity compared to non-overweight boys for all variables (mean activity rating: 2.40±0.20 vs 2.60±0.19; % time in MVPA: 39.0±12.5 vs. 47.6±12.7; total counts/hr: 50.5±14.4 vs. 60.0±14.5; MVPA intervals: 27.2±10.5 vs. 33.7±8.5 and VPA intervals: 4.9±3.1 vs. 6.7±2.8).  *Girls:* No significant differences in physical activity variables between overweight and non-overweight girls (p≥0.05; mean activity rating: 2.50±0.19 vs 2.49 ±0.20; % time in MVPA: 42.2±12.8 vs. 41.6±12.5; total counts/hr: 51.9±15.8 vs. 52.1±15.7; MVPA intervals: 28.3±10.8 vs. 28.5±11.1 and VPA intervals: 4.7±3.0 vs. 5.6±3.7). |
| Lioret et al. 2007 [26]; France | Cross-sectional | n=593; age range: 3-5 yr. | LTPA (no LTPA, intermediate, high (80^th^ percentile)) assessed via proxy-report questionnaire. | Non-overweight, overweight/obesity (IOTF) assessed via proxy-reported height and weight (BMI). | Children who participated in intermediate LTPA (OR=0.2; 95%CI: 0.1, 0.7) were significantly less likely to be overweight/obese compared to children who participated in no LTPA.  Children who participated in high LTPA (OR=0.5; 95%CI: 0.2, 1.3) were not significantly less likely to be overweight/obese compared to children who participated in no LTPA. |
| Kuzik & Carson, 2016 [27]; Canada | Cross-sectional | n=100; mean age: 38.5 mo. | Accelerometer-derived LPA (hr/day) and MVPA (hr/day) at child care.  MVPA (hr/day) outside of child care assessed via proxy-report questionnaire. | BMI z-score (WHO) assessed via objectively measured height and weight. | LPA (β=0.31; 95%CI: -0.22, 0.84) and MVPA (β=0.41; 95%CI: -0.26, 1.08) in child care and MVPA outside of child care (β=-0.03; 95%CI: -0.40, 0.34) were not significantly associated with BMI z-score (p≥0.05). |
| Burdette & Whitaker, 2005 [28];  United States | Cross-sectional | n=2620; mean age: 39 mo. | Outdoor play time (min/day) assessed via proxy-report interview. | BMI (kg/m^2^) assessed via objectively measured height and weight. | Outdoor play time was not significantly correlated with BMI (data not shown). |
| Bonvin et al. 2012 [29]; Switzerland | Cross-sectional | n=251; mean age 3.4 yr. | Accelerometer-derived child care TPA (cpm), MVPA (epoch/hr), VPA (epoch/hr). | Healthy weight and overweight/obesity (IOTF) assessed via objectively measured height and weight (BMI). | Between healthy weight and overweight groups, TPA (610±211 vs. 587±201), MVPA (29±13 vs. 29±13), VPA (8±6 vs. 7±5) at child care were not significantly different (p≥0.05). |
| Ansari et al. 2015 [30]; United States | Cross-sectional* | n=2810; mean age: 45 mo. | Outdoor play (min/day) of class assessed via proxy-report questionnaire. | BMI (kg/m^2)^ and non-obese (BMI percentile <95) and obese (BMI percentile ≥95) assessed via objectively measured height and weight. | Outdoor play was significantly associated with BMI (β=-0.05; 95%CI: -0.09, -0.01).  Children who engaged in more outdoor play were significantly less likely to be obese (OR=0.99; 95%CI: 0.98, 0.99). |
| Sääkslahti et al. 1999 [31]; Finland | Cross-sectional | n=105; mean age: 3.75 yr. | Very active indoor play, very active outdoor play, high activity of play activities (indoors and outdoors) on the weekend  assessed via proxy-report log. | BMI (kg/m^2^) and weight percentiles (Finnish growth carts) were assessed via objectively measured height and weight. | No significant correlations observed between any of the play variables and BMI (all r ≤ ±0.09) and weight percentiles (all r ≤ ±0.09). |
| Sijtsma et al. 2015 [32]; Netherlands | Cross-sectional | n=298; mean age: 3.9 yr. | Accelerometer-derived TPA (cpm), LPA (% of time), MPA (% of time), and VPA (% of time). | BMI z-score (Netherlands reference data) assessed via objectively measured height and weight.  Waist circumference z-scores (Netherlands reference data) objectively measured. | TPA was significantly correlated with BMI z-score (r=0.147; p=0.042) but not waist circumference z-score (r=0.143; p=0.072).  LPA was not significantly correlated with BMI z-score (data not shown) but was significantly correlated with waist circumference z-score (r=-0.173; p=0.029).  MPA was significantly correlated with BMI z-score (r=0.149; p=0.039) and waist circumference z-score (r=0.191; p=0.016).  VPA was significantly correlated with BMI z-score (r=0.159; p=0.027) and waist circumference z-score (r=0.262; p=0.001). |
| Jouret et al. 2007 [33]; France | Cross-sectional | n=593; mean age: 3.9 yr. | Participation in organized sport (yes/no) assessed via proxy-report questionnaire. | Overweight (French reference curves; ≥90^th^ percentile) assessed via objectively measured height and weight (BMI). | *Boys:* Children that participated in organized sport compared to those that did not were not significantly more likely to be overweight (OR=0.74; 95%CI: 0.21, 2.60; p=0.6371).  *Girls:* Children who participated in organized sport compared to those that did not were significantly more likely to be overweight (OR=3.88, 95%CI: 1.68, 8.90). |
| Sijtsma et al. 2015 [34]; Netherlands | Cross-sectional | n=759; mean age: 3.9 yr. | Outdoor play (min/day) assessed via proxy-report questionnaire. | BMI (kg/m2) assessed via objectively measured height and weight (BMI). | Outdoor time was not significantly correlated with BMI (r=-0.010; p=0.794). |
| Pallan et al. 2013 [35]; England | Cross-sectional | n=5817 (175 schools); age range: 4-5 yr. | Physical education (10 min/wk) at the school level assessed via proxy-report questionnaire. | BMI z-score (UK 1990 BMI reference data) assessed via objectively measured height and weight. | More 10 min/wk increments of physical education at the school level was significantly associated with lower BMI z-scores at the individual level (β=-0.026; 95%CI: -0.047, -0.005). |
| Collings et al. 2013 [36]; England | Cross-sectional | n=398; mean age: 4.1 yr. | Accelerometer-derived LPA (min/day), MPA (min/day), VPA (min/day), MVPA (min/day). | Body composition (percentage of body fat, fat mass index, trunk fat mass index, lean mass index) objectively measured using dual-energy X-ray absorptiometry. | LPA was significantly associated with percentage of body fat (β=0.53; 95%CI: 0.0091, 0.98) and fat mass index (% change=2.60; 95%CI: 0.38, 5.00) but not with trunk fat mass index (β=2.50; 95%CI: -0.40, 5.00), lean mass index (β=-0.033; 95%CI: -0.12, 0.053).  MPA was not significantly associated with percentage of body fat (β=−0.16; 95%CI: −0.54, 0.22), fat mass index (β=-0.69; 95%CI: -2.62, 1.27), trunk fat mass index (β=−0.32; 95%CI: -2.83, 2.25), and lean mass index (β= 0.022; 95%CI: -0.046, 0.089).  MVPA was significantly associated with percentage of body fat (β=−0.53; −0.98, −0.091), fat mass index (% change=−2.60; 95%CI: −5.00, −0.38) but not with trunk fat mass index (β= −2.50; 95%CI: -5.00, 0.40) or lean mass index (β=0.033; 95%CI: −0.053, 0.12).  VPA was significantly associated with percentage of body fat (β=−0.36; 95%CI: −0.55, −0.17), fat mass index (% change= −1.75; 95%CI: −2.65, −0.85), trunk fat mass index (% change= -1.90; 95%CI: −3.10, −0.68) but not with lean mass index (β= 0.014; 95%CI: −0.023, 0.052).  A significant p-trend was observed for quartiles of VPA (<11, 11-21, 22-36, >37 min/day) for percentage of body fat, fat mass index, and trunk fat mass index). |
| Williams et al. 2008 [37]; United States | Cross-sectional | n=198; mean age: 4.2 yr. | Accelerometer-derived LPA, MVPA, VPA (% of time). | BMI z-score assessed via objectively measured height and weight. | MVPA was significantly correlated with BMI z-score (r=0.14; p<0.05). LPA (r=0.01) and VPA (r=0.13) were not significantly correlated with BMI z-score (p≥0.05). |
| Watanabe et al. 2011 [38]; Japan | Cross-sectional | n=1867; mean age: 4.2 yr. | Outdoor play (hr/day) assessed via proxy-report questionnaire. | Non-overweight, overweight/obese (IOTF) assessed via objectively measured height and weight (BMI). | Children who spent <1 hour compared to >1 hour playing outside were not significantly more likely to be overweight/obese (OR=1.06; 95%CI: 0.64, 1.74). |
| Hajian-Tilaki et al. 2013 [39]; Iran | Cross-sectional | n=760; mean age (boys): 4.23 yr; mean age (girls): 4.24 yr. | Outdoor physical activity (playing, riding bicycle, walking; hr/wk) assessed via proxy-report questionnaire. | Non-overweight and Overweight/obesity (CDC; ≥85^th^ percentile) assessed via objectively measured heights and weights. | Children participating in moderate (7-20hr/wk; OR=0.77; 95%CI: 0.54, 1.10, p=0.16) and high (≥21hr/wk; OR=0.98; 95%CI: 0.56, 1.72, p=0.96) levels of outdoor physical activity, compared to low (<7hr/wk) levels, were not significantly more likely to be overweight/obese. |
| Jones et al. 2009 [40]; Australia | Cross-sectional | n=138; mean age: 4.3 yr. | Accelerometer-derived MVPA (min/day) and TPA (cpm).  Weekend and weekday active play (hr/day) assessed via proxy-report questionnaire. | Non-overweight and overweight/obese (IOTF) assessed via objectively measured height and weight (BMI). | Active play on weekdays was significantly higher in the non-overweight group (3.49 ± 2.10) compared to the overweight/obese group (2.43±1.12).  No significant differences were observed between non-overweight and overweight/obese groups in TPA (865.71±226.18 vs. 961.08±213.05), MVPA (32.95±25.24 vs. 28.99±22.55), and active play on the weekend (4.00±1.91 vs. 3.90±1.65). |
| Pfeiffer et al. 2009 [41]; United States | Cross-sectional | n=331; mean age: 4.3 yr. | Accelerometer-derived MVPA and TPA (min/hr). | BMI z-score (CDC) assessed via objectively measured height and weight. | MVPA was significantly positively correlated with BMI z-score (r=0.18; p≤0.001).  TPA was significantly positively correlated with BMI z-score (r=0.16; p≤0.01). |
| Lin et al. 2016 [42]; Taiwan | Cross-sectional | n=264; mean age: 52.6 mo. | Duration and frequency of physical activity assessed via the proxy-reported Preschool- Aged Children's Physical Activity Questionnaire. | Normal weight, overweight, and obesity assessed via objectively measured height and weight (BMI). | Physical activity was lower in overweight and obese children compared to normal-weight children (91.8 vs. 167.3 min; p<0.001). |
| Jago et al. 2005 [43]; United States | Cross-sectional* | n=142; mean age: 4.4 yr. | MVPA (min/hr) assessed via heart rate monitor. | BMI ((kg/m^2^) assessed via objectively measured height and weight. | MVPA was not significantly correlated with BMI (r=−0.027; p≥0.05). |
| Klesges et al. 1990 [44]; United States | Cross-sectional | n=137; mean age (baseline): 4.44 yr. | Accelerometer-derived TPA (standardized activity score). | Relative weights (comparison of heights and weight to national norms in the United States) assessed via objectively measured height and weight.  Skin-fold thicknesses (girls: triceps, suprailiac, abdomen; boys: triceps, subscapular, chest) objectively measured.  Waist and hip circumference objectively measured. | TPA was significantly correlated with hip circumference (r=0.18; p<0.05).  No other significant correlations were observed between TPA and adiposity measures (all r ≤ ±0.14; p≥0.05). |
| Leppänen et al. 2016 [45]; Sweden | Cross-sectional | n=307; mean age: 4.48 yr. | Accelerometer-derived LPA (min/day), MPA (min/day), VPA (min/day), MVPA (min/day), and 25th, 50th, 75th, 90th and 95th percentiles of 10-s sum of vector magnitude. | BMI (kg/m^2^) assessed via objectively measured height and weight.  Fat mass (%), fat mass index (kg m^− 2^), fat free mass index (kg m^− 2^) objectively measured using air-displacement plethysmography through the pediatric option for BodPod. | 95^th^ percentile of vector magnitude was significantly associated with fat free mass index (β=0.07; 95%CI: 0.03, 0.10) but not with BMI (β=0.04; 95%CI: -0.01, 0.10), percent fat mass (β=−0.17; 95%CI: −0.34, 0.00), fat mass index (β=−0.02; 95%CI: -0.05, 0.02) or waist circumference (β=0.07; 95%CI: −0.07, 0.21).  90^th^ percentile of vector magnitude was significantly associated with percent fat mass (β=−0.25; 95%CI: −0.51, −0.00) and fat free mass index (β=0.08; 95%CI: 0.03, 0.13) but not with BMI (β=0.05; 95%CI: −0.03, 0.12), fat mass index (β=-0.03; 95%CI: −0.08, 0.03), or waist circumference (β=0.12; 95%CI: −0.08, 0.32).  No significant associations were observed between 25^th^ and 50^th^ percentiles of vector magnitude with any of the adiposity measures (data not shown).  No significant associations were observed between 75^th^ percentiles of vector magnitude and BMI (β= 0.04; 95%CI: −0.07, 0.15), percent fat mass (β=−0.26; 95%CI: −0.63, 0.12), fat mass index (β=−0.02; 95%CI: -0.10, 0.05), fat free mass index (β= 0.08; 95%CI: −0.00, 0.15) or waist circumference (β= 0.17; 95%CI: −0.13, 0.46).  No significant associations were observed between LPA with any of the adiposity measures (data not shown).  MPA was not significantly associated with BMI (β=−0.00; 95%CI: −0.04, 0.04), percent fat mass (β=−0.04; 95%CI: −0.17, 0.10), fat mass index (β=−0.00; 95%CI: -0.03, 0.03), fat free mass index (β=0.00; 95%CI: −0.03, 0.03), and waist circumference (β=0.07; 95%CI: −0.04, 0.18).  MVPA was significantly associated with percent fat mass (β= −0.25; 95%CI: −0.45, −0.05) and fat free mass index (β=0.06; 95%CI: 0.01, 0.10) but not with BMI (β=0.02; 95%CI: −0.04, 0.08), fat mass index (β=−0.04; 95%CI: -0.08, 0.01), or waist circumference (β=0.06; 95%CI: −0.10, 0.22).  VPA was significantly associated with fat free mass index (β=0.19; 95%CI: 0.08, 0.31) but not with BMI (β=0.12; 95%CI: −0.05, 0.29), percent fat mass (β -0.51; 95%CI: −1.08, 0.05), fat mass index (β=−0.06; 95%CI: −0.17, 0.06), and waist circumference (β=0.05; 95%CI: −0.40, 0.50). |
| España-Romero et al. 2013 [46]; United States | Cross-sectional | n=357; mean age: 4.5 yr. | Accelerometer-derived MVPA (min/hr). | BMI z-score (CDC) assessed via objectively measured height and weight.  Waist circumference (cm) and percentiles of waist circumference (NHANES III) objectively measured. | *Boys:* MVPA was significantly associated with BMI z-score (β=0.080; SE=0.039) but not with waist circumference (β=0.233; SE=0.160).  MVPA was not significantly associated with BMI z-score at the 10^th^ (β=0.012; SE=0.089), 25^th^ (β=0.053; SE=0.053), 75^th^ (β=0.101; SE=0.055), 90^th^ percentiles (β=0.134; SE=0.115) or waist circumference at the 10^th^ (β=0.040; SE=0.239), 25^th^ (β=0.288; SE=0.153), 50^th^ (β=0.083; SE=0.175), 75^th^ (β=0.380; SE=0.223), 90^th^ percentiles (β=0.527; SE=0.491) (p≥0.05). MVPA was significantly associated with BMI z-score at the 50^th^ percentile (β=0.097; SE=0.048).  *Girls:* MVPA was not significantly associated with BMI z-score (β= −0.024; SE=0.036) or waist circumference (β= −0.190; SE=0.187).  MVPA was not significantly associated with BMI z-score at the 10^th^ (β= −0.010; SE=0.054), 25^th^ (β=0.003; SE=0.053), 50^th^ (β= -0.008; SE=0.039), 75^th^ (β= −0.036; SE=0.047), 90^th^ percentiles (β= −0.105; SE=0.070) or waist circumference at the 10^th^ (β= −0.096; SE=0.228), 25^th^ (β= 0.118; SE=0.227), 50^th^ (β= −0.092; SE=0.130), 75^th^ (β= -0.160; SE=0.221) percentiles (p≥0.05). MVPA was significantly associated with waist circumference at the 90^th^ percentile (β=−0.599; SE=0.302). |
| Cox et al. 2012 [47]; Australia | Cross-sectional | n=135; mean age: 4.5 yr. | LPA and MVPA (min/day) assessed via the proxy-report Physical Activity Questionnaire for Preschool-aged Children (Pre-PAQ). | BMI z-scores (CDC) assessed via proxy-reported height and weight. | LPA (r=0.02) and MVPA (r=0.02) were not significantly correlated with BMI z-scores (p≥0.05). |
| Söderström et al. 2012 [48]; Sweden | Cross-sectional | n=169; mean age: 4.5 yr | TPA (steps/min) objectively measured using pedometers.  Outdoor time clocked at the day care center. | Normal or overweight/obese (IOTF) assessed via objectively measured height and weight (BMI).  Waist circumference (cm) objectively measured. | TPA (p=0.14) and outdoor time (p=0.07) were not significantly different between normal or overweight/obese groups and waist circumference groups (note: waist circumference groups not defined; p≥0.05). |
| Jiang et al. 2006 [49]; China | Cross-sectional | n=930; mean age: 4.6 yr. | VPA (hr/day) outside of kindergarten assessed via proxy-report questionnaire. | Non-overweight, overweight/obesity (IOTF) assessed via objectively measured height and weight (BMI). | VPA (<1hr/day) was not significantly associated with overweight (data not shown; p≥0.05). |
| Cardon et al. 2016 [50]; Belgium, Bulgaria, Germany, Greece, Poland, and Spain | Cross-sectional | n=3301; mean age (boys): 4.75 yr; mean age (girls): 4.76 yr. | Steps (steps/day) objectively measured with a pedometer or accelerometer, depending on the country.  Outdoor play on weekdays (hr/day) and weekend days (hr/day) assessed via proxy-report questionnaire. | BMI (kg/m^2^) and normal weight and overweight/obese (IOTF) assessed via objectively measured heights and weight. | *Boys:* There was no associations observed between steps/day on weekdays (β=0.003, 95%CI: 0.000, 0.000) or weekend days (β=−0.047, 95%CI: 0.000, 0.000) and hr/day of outdoor play on weekdays (β=−0.031, 95%CI: −0.065, 0.021) or weekend days (β= 0.019, 95%CI: −0.014, 0.036) and BMI.  Engaging in <11500 steps, compared to ≥11500 steps on weekdays (OR=1.269, 95%CI: 0.905, 1.780) and weekend days (OR=1.036, 95%CI: 0.721, 1.488) was not significantly associated with being overweight/obese (p≥0.05).  Engaging in <2hr/day of active play outdoors, compared to ≥2hr/day, on weekdays (OR=1.299, 95%CI: 0.917, 1.840) was not significantly associated with being overweight/obese (p≥0.05).  Engaging in <4hr/day of active play outdoors, compared to ≥4hr/day, on weekend days (OR=1.060, 95%CI: 0.748, 1.501) was not significantly associated with being overweight/obese (p≥0.05).  *Girls:* There was no associations observed between steps/day on weekdays (β=0.012, 95%CI: 0.000, 0.000) or weekend days (β= −0.014, 95%CI: 0.000, 0.000) and hr/day of outdoor play on weekdays (β= 0.015, 95%CI: -0.035, 0.064) or weekend days (β= −0.011, 95%CI: −0.036, 0.020) (p≥0.05) and BMI.  Engaging in <11500 steps, compared to ≥11500 steps on weekdays (OR=0.976, 95%CI: 0.683, 1.393) or weekend days (OR=0.826, 95%CI: 0.571, 1.194) was not significantly associated with being overweight/obese (p≥0.05).  Engaging in <2hrs/day, compared to ≥2hr/day, on weekdays (OR=0.930, 95%CI: 0.663, 1.315) was not significantly associated with being overweight/obese (p≥0.05).  Engaging in <4hr/day, compared to ≥4hr/day on weekend days (OR=1.057, 95%CI: 0.754, 1.481) was not significantly associated with being overweight/obese (p≥0.05). |
| Eijkemans et al. 2008 [51]; Netherlands | Cross-sectional* | n=305; mean age: 4.9 yr. | Accelerometer-derived TPA (cpm), VPA (min/day), MVPA (# of episodes). | Underweight, normal weight, overweight, obese (IOTF) assessed via objectively measured height and weight (BMI). | The obese group compared to the normal weight group participated in significantly less VPA (GMR=0.49; 95%CI: 0.26, 0.92) and MVPA (GMR=0.35; 0.17, 0.74) but not TPA (GMR=0.88; 95%CI: 0.73, 1.06).  No significant differences were observed in TPA (GMR=0.98; 95%CI: 0.90, 1.07), VPA (GMR=0.89; 95%CI: 0.68, 1.71) and MVPA (GMR=1.07; 95%CI: 0.77, 1.47) between underweight and normal weight group (reference group).  No significant differences were observed in TPA (GMR=1.03; 95%CI: 0.93, 1.14), VPA (GMR=0.92; 95%CI: 0.66, 1.28) and MVPA (GMR=0.99; 95%CI: 0.67, 1.47) between overweight and normal weight groups (p≥0.05). |
| Sääkslahti et al. 2003 [52];  Finland | Cross-sectional* | n=155; mean age: 4.9 yr. | High-activity playing (indoor and outdoor; hr/day) assessed via proxy-report log. | BMI (kg/m^2^) assessed via objectively measured height and weight. | *Boys*: High-activity playing was not significantly correlated with BMI (r=-0.16; p≥0.05.)  *Girls*: High-activity playing was not significantly correlated with BMI (r=-0.15; p≥0.05). |
| Anderson et al. 2008 [53]; United States | Cross-sectional | n=777; mean age: 59.6 mo. | Active play (times/wk) assessed via proxy-repot questionnaire. | Non-obese and obese (CDC; ≥95 percentile) assessed via objectively measured height and weight (BMI). | *Boys*: the proportion of boys in the low active play group (<7 times/wk) was not significantly different between non-obese (OR=25.3; 95%CI: 19.3, 31.2) and obese groups (OR=37.6; 95%CI: 19.9, 55.3).  *Girls*: the proportion of girls in the low active play group (<7 times/wk) was not significantly different between non-obese (OR=36.0; 95%CI: 30.1, 41.9) and obese groups (OR=32.2; 95%CI: 18.3, 46.1). |
| de Carvalho Cremm et al. 2011 [54]; Brazil | Cross-sectional | n=302; age: <6 yr. | Active transportation (walks/bikes) to school (yes/no) and physical activity (yes/no) assessed via proxy report interview. | Overweight (WHO; ≥1 SD) assessed via objectively measured height and weight (BMI). | Active transportation (OR=1.70; 95%CI: 1.0, 2.91) and physical activity (p>0.20) were not associated with overweight (p≥0.05). |

β = standardized beta; BMI = body mass index; CDC = Centers for Disease Control and Prevention; cpm = counts per minute; GMR = geometric mean ratio; hr = hour; IOTF = International Obesity Task Force; kg = kilogram; LPA = light-intensity physical activity; LTPA = leisure-time physical activity; m = meter; METs = metabolic equivalent of task; min = minute; mo = month; MPA = moderate-intensity physical activity; MVPA = moderate- to vigorous-intensity physical activity; NHANES = National Health and Nutrition Examination Survey; OR = odd ratio; OSRAP = the observation system for recording activity in preschools; RCT = randomized controlled trial; SD = standard deviation; SE= standard error; TPA = total physical activity; UK = United Kingdom; VPA = vigorous-intensity physical activity; WHO = World Health Organization; wk = week; yr = year; 95%CI = 95% confidence interval.

*Longitudinal study but only cross-sectional data eligible for this review.

**Table S2.** Summary of studies for motor development

| Refid #. Author (year); country | Study Design | Sample | Exposure | Outcome | Main Findings |
| --- | --- | --- | --- | --- | --- |
| De Vries et al. 2015 [1]; Netherlands | RCT | n=161; age (baseline): 2 wk. | Intervention group: Parents received recommendations from a nurse in regards to a physical activity stimulation program at age 2 wk, 2, 4, 8, 11 mo.  Control group: Standard care with no activity recommendations. | Motor development was objectively measured using the Dutch Second Edition of the Bayley Scales of Infant and Toddler, Third Edition (Bayley-III; Range: 1-19; higher better) at age 2.5 yr. | There were no significant differences in motor development between the intervention (10.2±1.7) and control (10.6±2.3) groups at age 2.5 yr (p=0.20).  Note: There were no significant differences in accelerometer-derived TPA (cpm x 10^6^) between intervention (2.26±0.53) and control groups (2.26±0.58) at age 2.5 yr (p=0.99). |
| Porter, 1972 [55]; Philippines | RCT | n=130; mean age: 18.3 wk. | Intervention: Planned passive cycling - 2x5 min sessions alternated with equal rest, 2 times/day, 6 day/wk, for 2 mo.  Control: Regular child-rearing practices. | Motor development was objectively measured using the Gessell Development Schedules- Development Quotient (units unknown) at baseline, 1 mo follow-up, and 2 mo follow-up. | The intervention group had significant gains in motor development (15.55±21.33 at 1 mo follow-up and 29.03±22.19 at 2 mo follow-up) compared to the control group (1.40±8.69 at 1 mo follow-up and 12.01±17.47 at 2 mo follow-up).  Note: It is unknown if the intervention resulted in a significant change in physical activity. |
| Teixeira Costa et al. 2015 [56]; Portugal | RCT | n=324; age range: 3-5 yr. | Intervention: Structured physical education program – 45 min, 2 times/wk for 24 wk.  Control: Standard program of preschool education that did include some physical activity but not structured. | Psychomotor skills (coordination and balance, laterality; units unknown) objectively measured at baseline and 24 wk follow-up. | There was a significant group (intervention vs. control) by test (baseline and 24 wk follow-up) interaction for coordination and balance and laterality (all p<0.001), which showed increases in the psychomotor skills were significantly larger in the experimental group compared to the control group.  Coordination and balance: 3 year olds (intervention group mean difference: 3.34; control group mean difference: 1.15); 4 year olds (intervention group mean difference: 2.58; control group mean difference: 0.34); 5 year olds (intervention group mean difference: 2.68; control group mean difference: 0.32).  Laterality: 3 year olds (intervention group mean difference: 2.32; control group mean difference: 1.06); 4 year olds (intervention group mean difference: 1.45; control group mean difference: 0.42); 5 year olds (intervention group mean difference: 1.65; control group mean difference: -0.20).  Note: It is unknown if the intervention resulted in a significant change in physical activity. |
| Mostafavi et al. 2013 [57]; Iran | RCT | n=90; mean age (intervention): 59.7 mo; mean age (control 1): 58.1 mo; mean age (control 2): 59.0 mo. | Intervention group: Sports, Play and Active Recreation for Kids (SPARK) physical education program – 3 times/wk for 8 wk.  Control group 1: gymnastics – 3 times/wk for 8 wk.  Control group 2 – routine physical education program | Fundamental motor skills (total, locomotor and object control) objectively measured using the Test of Gross Motor Development (TGMD-2; units unknown) at baseline and 8 wk follow-up. | Baseline and 8 wk follow-up total scores were significantly different in the SPARK group (14.2±7.1) but not the gymnastics (3.1±0.9) or routine activity (2.9±0.11) groups.  Baseline and 8 wk follow-up locomotor skills were significantly different in the SPARK group (2.5±2.0) but not the gymnastics (0.4±0.1) or routine activity (0.8±0.0) groups.  Baseline and 8 wk follow-up object control skills were significantly different in the SPARK group (2.2±1.7) but not the gymnastics (0.8±0.3) or routine activity (0.9±0.1) groups.  Follow-up total scores were significantly higher in the intervention group compared to the gymnastic (mean difference: −12.93; 95%CI: −15.57, −10.29) and routine activity (mean difference= −13.85; 95%CI: −16.44, −11.26) groups (p<0.001).  Follow-up objective control skills were significantly higher in the intervention group compared to the gymnastic (mean difference= −2.44; 95%CI: −3.17, −1.72) and routine activity (mean difference= −2.39; 95%CI: −3.1, 1.68) groups.  Follow-up locomotor skills were significantly higher in the intervention group compared to the routine activity group (mean difference= −2.28; 95%CI: −2.93, −1.63).  Follow-up locomotor skills were significantly higher in the gymnastics group compared to the intervention group (mean difference=1.89; 95%CI: −2.56, −1.72).  Note: It is unknown if the intervention resulted in a significant change in physical activity. |
| Bonvin et al. 2013 [2]; Switzerland | Clustered RCT | n=1467; mean age: 3.3 yr. | Intervention: Government-led physical activity program (no specific time component or curriculum) for 10 mo.  Control: Regular program. | Motor skills objectively measured using measures adapted from the Zurich Neuromotor Assessment test (range 0-20; higher better) at baseline and 10 mo follow-up. | No significant difference in motor skills were observed between the mean individual changes in the intervention group (baseline: 12.4±3.5; follow-up: 14.2±2.9) and the control group (baseline: 12.5±3.5; follow-up: 14.2±2.8).  Note: No significant difference in TPA (cpm) were observed between the mean individual changes in the intervention group (baseline: 620 ±278; follow-up: 765±340) and the control group (baseline: 600 ±206; follow-up: 711±219). No significant differences in MVPA (epochs/hr) were observed between the mean individual changes in the intervention group (baseline: 29.2±14; follow-up: 37.2±17.1) and the control group (baseline: 28.1±12.5; follow-up: 35.9±13.7). No significant differences in VPA (epochs/hr) were observed between the mean individual changes in the intervention group (baseline: 8.1±6.1; follow-up: 10.3±7.5) and the control group (baseline: 7.4±5.3; follow-up: 9.2±6.2). |
| Jones et al. 2011 [3]; Australia | Clustered RCT | n=97; mean age (baseline): 4.13 yr. | Intervention: Structured activities  designed to improve motor  skills - 3 times/wk for  20 wk in childcare.  Control: Usual  care. | Movement skill development (run (range: 0-8), hop (range 0-10), jump (range: 0-8), catch (range 0-6), and kick (range: 0-8) was objectively measured using the Test of Gross Motor Development  (second edition) at baseline and 6 mo follow-up. | Greater improvements were observed in the intervention group compared with the control group from baseline to 6 mo follow-up in jumping skill development (adjusted mean difference=1.41; 95%CI: 0.69, 2.13) and sum of the five motor skills development (adjusted mean difference=2.08; 95%CI: 0.76, 3.40) .No significant differences were found between the intervention and control groups from baseline to 6 mo follow-up in running (adjusted mean difference=0.02; 95%CI: −0.05, 0.57), hopping (adjusted mean difference=0.65; 95%CI: −0.11, 1.42), catching (adjusted mean difference=0.16, 95%CI: −0.32, 0.63), and kicking (adjusted mean difference=0.42, 95%CI: −0.18, 1.01).  *Note:* Change in TPA from baseline to the last two wk of the intervention assessed via counts/min were significantly higher in the intervention compared to the control group (adjusted mean difference=110.48, p=0.01). |
| Draper et al. 2012 [58]; South Africa | Non-randomized Intervention | n=118; mean age (intervention): 4.6 yr; (control): 4.4 yr. | Intervention: Supervised free play to structured activities for 3-4 sessions/wk, 45-60 min/session for 8 mo.  Control: Usual care. | Gross motor skills (locomotor: run, gallop, hop, leap, horizontal jump, slide, total; object control: strike a ball, bounce a ball, catch, kick, overhand throw, understand roll, total; units unknown) objectively measured using the Test of Gross Motor Development Version-2 (TGMD-2) at 7 mo follow-up. | Significant differences were observed in the total locomotor scores between the intervention (43.9±5.65) and control (37.9±9.71) groups (p<0.005) at 7 mo follow-up.  Significant differences existed in sliding between the intervention (7.1±2.36) and control (5.1±3.16) groups (p<0.001). No significant differences existed in running, (intervention: 7.6±0.95; control: 6.9±1.86), galloping (intervention: 6.4±3.01; control: 5.4±3.38), hopping (intervention: 9.7±0.87; control: 8.7±2.49), leaping (intervention: M=5.8±0.72; control: 5.1±1.84), and horizontal jumping (intervention: 7.4±1.39; control: 6.5±2.43) at 7 mo follow-up.  Significant differences existed in the object control total score between the intervention (42.10±5.03) and control (35.12±10.10) groups (p<0.01) at 7 mo follow-up.  Significant differences existed in striking a ball (intervention: 8.9±1.69; control: 6.9±2.90; p<0.001) and catching (intervention: 5.4±1.16; control: 4.3±1.84, p<0.01). No significant differences existed in bouncing a ball (intervention: 5.2±2.77; control: 4.5±3.06), kicking (intervention: 7.4 ± 1.40; control: 6.1±2.75), overhead throwing (intervention: 7.7±1.12; control: 6.7±2.16), and underhand rolling (intervention: 7.7±0.73; control: 6.7±2.36) at 7 mo follow-up.  *Note:* It is unknown if the intervention resulted in a significant change in physical activity. |
| Livonen et al. 2011 [59]; Finland | Non-randomized Intervention | n=84; mean age: 55.4 mo. | Intervention: Physical education class 45 min, 2 times/wk with a physical education curriculum for 8 mo.  Control: Physical education class 60 min/wk with no specific structured program. | Fundamental movement skills (dynamic balance (sec), running speed (sec), length of standing broad-jump (cm), manipulative skills (sum variable of throwing-catching combination, throwing at target and kicking ball at target; range: 0-25) objectively measured using the APM- Inventory at baseline (T1), T2, T3, and 12 mo follow-up (T4). | *Boys:* No significant differences in the 4 measurements were observed between the intervention and control group for dynamic balance (intervention: T1: 19.1±7.2, T2: 16.9±6.6, T3: 15.6±5.4, T4: 14.2±6.2; control: T1: 22.6±8.1, T2: 20.8±5.8, T3: 18.0±5.1, T4: 14.2±4.1) standing broad-jump (intervention: T1: 85.0±21.1, T2: 92.6±18.8, T3: 102.1±17.9, T4: 101.3±21.6; control: T1: 79.5±19.6, T2: 91.3±21.2, T3: 100.6±19.9, T4: 110.6±14.2), and manipulative skills (intervention: T1: 12.4±4.2, T2: 14.9±4.0, T3: 16.2±4.9, T4: 16.6±4.9; control: T1: 12.5±3.3, T2: 14.6±4.7, T3: 14.2±4.1, T4: 17.6±5.1).  Running speed significantly improved in the intervention group (T2: 3.1±0.3; T3: 2.9±0.2) but not the control group (T2: 2.9±0.3; T3: 2.9±0.2) between measurement 2 and 3 only but not at the other time points (intervention: T1: 3.3±0.4, T4: 2.8±0.2; control: T1: 3.3±0.4, T4: 2.8±0.1).  *Girls:* No significant differences in the 4 measurements were observed between the intervention and control group for dynamic balance (intervention: T1: 21.0±10.7, T2: 19.1±10.4, T3: 16.3±8.8, T4: 13.0±7.0; control: T1: 24.1±12.8, T2: 22.9±6.6, T3: 19.6±8.7, T4: 14.6±5.2), running speed (intervention: T1: 3.3±0.5, T2: 3.2±0.6, T3: 3.0±0.3, T4: 2.8±0.2; control: T1: 3.3±0.4, T2: 3.0±0.3, T3: 3.1±0.3, T4: 2.8±0.2), standing broad-jump (intervention: T1: 79.0±22.0, T2: 82.3±21.6, T3: 97.9±20.8, T4: 104.8±17.8; control: T1: 75.3±17.0, T2: 78.0±15.3, T3: 86.4±13.2, T4: 97.4±12.7), and manipulative skills (intervention: T1: 10.6±4.4, T2: 12.5±4.2, T3: 14.4±5.4, T4: 16.9±3.6; control: T1: 9.8±4.5, T2: 12.0±5.0, T3: 13.0±4.6, T4: 16.8±4.6).  Note: It is unknown if the intervention resulted in a significant change in physical activity. |
| Venetsanou et al. 2004 [60]; Greece | Non-randomized Intervention | n=66; mean age: 59.79 mo. | Intervention: Dance program – 45 min, 2 times/wk for 20 wk.  Control: Regular kindergarten curriculum | Motor proficiency objectively measured using the Motoriktestfürvier-bissechsjährige Kinder (MOT 4–6) test battery (Range 0-34) at baseline and 20 wk follow-up. | A significant difference in the baseline measure of motor proficiency was not observed between intervention and control group (mean difference=0.51; p>0.05). A significant difference in the 20 wk follow-up measure of motor proficiency was observed between intervention and control groups (mean difference=3.74; p<0.001).  Note: It is unknown if the intervention resulted in a significant change in physical activity. |
| Monsalves-Alvarez et al. 2015 [6]; Chile | Non-randomized Intervention | n=81; mean age: 4.0 yr. | Intervention: Physical activity classes: 45 min total (15 min sessions) 3 times/wk over 6 mo. | Motor skills: 12-meter run (segments/weight) and standing long jump (cm/height) objectively measured at baseline and 6 mo follow-up. | *Boys:* There was a significant improvement in the 12-meter run (baseline: 0.45±0.69; follow-up: 0.30±0.07) and standing long jump (baseline: 0.43±0.17; follow-up: 0.71±0.13) from baseline to 6 mo follow-up.  *Girls:* There was a significant improvement in the 12-meter run (baseline: 0.37±0.08; follow-up: 0.33±0.07) and standing long jump (0.35±0.14 to 0.55±0.19) from baseline to 6 mo follow-up.  Note: It is unknown if the intervention resulted in a significant change in physical activity. |
| Sigmundsson et al. 2010 [61]; Iceland | Non-randomized Intervention | n=38; age range (baseline): 2-7 mo. | Intervention: Baby swimming lesson program for 2 hr/wk for at least 4 mo.  Control: No baby swimming experience. | Motor ability (total, manual dexterity (posting coins, threading beads, bicycle trail), ball skills (catching bean bag, rolling ball into goal), balance (one-leg balance, jumping over cord, walking heels raised)) objectively measured using the Movement Assessment Battery for Children (units unknown) at age 4 yr. | There was no significant difference between intervention and control groups in total motor ability (intervention: 4.7±3.5; control: 6.5±4.3), overall manual dexterity (intervention: 0.5±1.0; control: 0.4±0.7), posting coins (intervention: 0.1±0.4; control: 0.2±0.6), threading beads (intervention: 0.4 ± 0.9; control: 0.1±0.3), bicycle trail (intervention: 0±0; control: 0.1±0.4), overall ball skills (intervention: 2.8±1.6; control: 3.8±2.5), catching bean bag (intervention: 2.2±1.6; control: 2.9±2.0), rolling ball into goal (intervention: 0.6±0.9; control: 0.9±1.3), overall balance (intervention: 1.5±2.2; control: 2.3±2.5), jumping over cord (intervention: 1.5±2.2; control: 1.7±2.0), walking heels raised (intervention: 0±0; control: 0.2±0.5) at age 4 yr. The intervention group compared to the control group had significantly better one-leg balance (intervention: 0.02±0.1; control: 0.4±0.8) at age 4 yr.  Note: It is unknown if the intervention resulted in a significant change in physical activity. |
| Krombholz, 2012 [7]; Germany | Non-randomized Intervention | n=559; mean age (baseline): intervention group, 55.1 mo; control group, 54.4 mo. | Intervention: Physical education and structured physical activity session: 45 min session at least 1 time/wk and physical activity sessions: 20 min on other days for 20 mo.  Control: Usual curriculum. Included physical education and structured physical activity session: 45 min session 1 time/wk. | Motor performance (motor coordination, physical fitness, dexterity) objectively measured using the Motor Test Battery (MoTB 3-7; SD) at baseline, 11 mo follow-up and 20 mo follow-up. | The intervention group improved their motor performance significantly more than the control group (interaction between trial (baseline, 11 mo follow-up, 20 mo follow-up) and group: p=0.001) over the three measurement periods of baseline (-0.08±0.45), 11 mo follow-up (0.03±0.46), 20 mo follow-up (0.12±0.49).  Note: It is unknown if the intervention resulted in a significant change in physical activity. |
| Kuo et al. 2008 [62]; Taiwan | Cross-sectional and Longitudinal | n (cross-sectional) =134;  n (longitudinal) =197; mean age (baseline): 4 mo. | Wakeful prone experience (yes/no) and duration (0, 1-19, 20-30, ≥40 min/day) at age 4 mo and wakeful prone preference (yes/no) at age 6 mo assessed via proxy-report questionnaire. | Gross and fine motor development objectively measured using the Comprehensive Developmental Inventory for Infants and Toddlers (CDIIT; units unknown) at ages 6 and 24 mo.  Achievement of developmental milestones (prone-specific: rolling, crawling-on-abdomen, crawling-on-4s; Non-prone milestones: transferring objectives, sitting, walking; mo) assessed via proxy-report questionnaire at ages 4, 6, 12, 24 mo. | *Cross-sectional:*  The prone preference group at age 6 mo had significantly higher gross motor development than the non-prone preference group at age 6 mo (prone: median=104, IQR=95-113 vs. non-prone: median=97, IQR=90-106; p=0.037). No significant differences existed between prone and non-prone preference for fine motor skills (prone: median=88, IQR: 82-106 vs non-prone: median=91, IQR: 84-99) at age 6 mo.  *Longitudinal:*  Prone and non-prone experience groups at age 4 mo did not have significantly different gross motor skills at ages 6 mo (prone: median=97, IQR=91-106 vs. non-prone: median=97, IQR=91-103); and 24 mo (prone: median=81, IQR=75-92 vs. non-prone: median=86, IQR=80-94), and fine motor skills at ages 6 mo (prone: median=91, IQR=84-99 vs. non-prone: median=91, IQR=84-99) and 24 mo (prone: median=95, IQR=88-102 vs. non-prone: median=95, IQR=88-102) (p≥0.05).  Higher duration of prone position at age 4 mo was significantly associated with higher gross motor development at age 6 mo (0 min/d: median=97, IQR=90-103; 1-19 min/d: median=97, IQR=90-106; 20-39 min/d: median=102, IQR=97-106; ≥40 min/d: median=106, IQR=95-111; p=0.018) but not at age 24 mo (0 min/d: median=86, IQR=80-94; 1-19 min/d: median=81, IQR=75-92; 20-39 min/d: median=85, IQR=76-93; ≥40 min/d: median=81, IQR=76-97). No differences between groups were observed for fine motor skills at ages 6 mo (0 min/d: median=91, IQR=84-99; 1-19 min/d: median=91, IQR=84-99; 20-39 min/d: median=84, IQR=84-99; ≥40 min/d: median=98, IQR=86-104) or at ages 24 mo (0 min/d: median=95, IQR=88-102; 1-19 min/d: median=95, IQR=88-102; 20-39 min/d: median=91, IQR=88-102; ≥40 min/d: median=99, IQR=93-107) (p≥0.05).  No significant differences existed between prone and non-prone preference for gross motor skills at age 24 mo (median=81, IQR=79-86 vs. median=85, IQR=79-86). No significant differences between groups were observed for fine motor development at age 24 mo (median=91, IQR=81-109 vs. median=95, IQR=88-102) (p≥0.05).  Infants in the prone experience group at age 4 mo crawled on abdomen significantly earlier than the non-prone experience group (7.0 mo vs. 7.3 mo; p<0.0167). No significant differences between groups were observed for the other milestones (rolling: 4.0 mo vs. 5.0 mo; crawling-on-4s: 8.0 mo vs. 9.0 mo; transferring objects: 5.0 mo vs. 5.0 mo; sitting: 7.0 mo vs. 7.0 mo; walking: 12.0 mo vs. 12.0 mo; p≥0.05).  Higher duration of prone position at age 4 mo was significantly associated with achieving rolling (0min/d: 5.0 mo, 1-19min/d: 4.0 mo; 20-39min/d: 5.0 mo; ≥40min/d: 3.5 mo), crawling-on-abdomen (0min/d: 7.3 mo, 1-19min/d: 7.0 mo; 20-39min/d: 6.0 mo; ≥40min/d: 5.0 mo), crawling-on-4s (0min/d: 9.0 mo, 1-19min/d: 8.0 mo; 20-39min/d: 7.3 mo; ≥40min/d” 6.5 mo), and sitting (0min/d: 7.0 mo, 1-19min/d: 7.0 mo; 20-39min/d: 6.0 mo; ≥40min/d: 6.0 mo) milestones earlier (p<0.0167)but not for transferring objectives (0min/d: 5.0 mo, 1-19min/d: 5.0 mo; 20-39min/d: 5.0 mo; ≥40min/d: 4.5 mo) or walking milestones (0min/d: 12.0 mo, 1-19min/d: 12.0 mo; 20-39min/d: 12.0 mo; ≥40min/d: 12.0 mo; p≥0.05).  The prone preference group at age 6 mo achieved crawling-on-abdomen (5.0 mo vs. 7.0 mo) and crawling-on-4s (6.0 mo vs. 9.0 mo) significantly earlier that the non-prone preference group (p<0.0167). No significant differences between groups were observed for sitting (6.5 mo vs. 7.0 mo) and walking (12.0 mo vs. 12.0 mo) milestones (p≥0.05). |
| de Kegel et al. 2012 [63]; Belgium | Cross-sectional | n=210; age range: 0-18 mo. | Play time in prone position (min/day) in first 6 mo and from 6 mo measured via proxy-report questionnaire. | Motor development objectively measured using the Alberta Infant Motor Scales (units unknown; AIMS). | Children in the prone position ≥30 min/day compared to <30 min/day (≤15 min/day and never) in the first 6 mo of life has higher motor development scores (1.96±5.94 vs. −0.92±6.02; p=0.002).  Children in the prone position ≥30 min/day compared to <30 min/day after the first 6 mo of life had higher motor development scores (1.71±6.43 vs. −1.32±7.50; p=0.013). |
| Dudek-Shriber & Zelazney, 2007 [64]; United States | Cross-sectional | n=100; mean age: 134.8 days. | Prone position while awake (hr/day) assessed via proxy-report questionnaire. | Motor development objectively measured using the Alberta Infant Motor Scale (units unknown; AIMS). | Infants who spent ≥1 hr/day compared to <1hr/day in the prone position while awake were significantly more likely to achieve 7/21 prone (Forearm support (1): OR=19.33; Forearm support (2): OR=5.41; Extended arm support: OR=3.60; Roll prone to supine: OR=3.69; Swimming: OR=4.14; Reaching: OR=3.42; Pivoting: OR=4.02), 3/9 supine (Supine hands to knees: OR = 1.84; Supine active extension: OR=2.21; Roll supine to prone (without rotation): OR=2.01), 3/12 sitting (Sit propped with arms: OR=4.12; un-sustained sit: OR=2.82; Sit with arm support: OR=3.46) milestones. |
| Matheny & Brown, 1971 [65]; United States | Cross-sectional* | n=112 (56 pairs of twins); age 12 mo. | Activity assessed via proxy-report interview. | Fine motor coordination (units unknown) assessed via proxy-report interview. | Amount of activity was significantly negatively associated with fine motor coordination (χ^2^=10.88; p<0.05). |
| Johansson et al. 2015 [21]; Sweden | Cross-sectional | n=120; mean age: 2.03 yr. | Accelerometer-derived TPA (CPM for vertical axis and vector magnitude), TPA (steps/day), low-intensity physical activity (min/day), high-intensity physical activity (min/day), 5 min bouts of low-intensity physical activity (min and frequency) and high-intensity physical activity (min and frequency). | Motor skills were objectively measured using the neurological examination technique for toddler-age (Low: <53; High ≥ 53). | There were no significant differences in TPA (vertical: low: 1922±443 vs. high: 1797±377, p=0.17; vector magnitude: low: 3236±541 vs. high: 3017±506, p=0.07; steps: low: 11527±1698 vs. high: 11114±1581, p=0.27), low-intensity physical activity (min: low: 275±29 vs. high: 264±34, p=0.15; no of 5 min bouts: low: 2.1±1.4 vs. high: 2.1±1.5, p=0.84; total time in 5 min bouts: low: 11±8 vs. high: 12±9, p=0.72), and high-intensity physical activity (min: low: 90±26 vs. high: 83±22, p=0.18; no of 5 min bouts: low: 0.2±0.3 vs. high: 0.2±0.3, p=0.66; total time in 5 min bouts: low: 1.3±2.0 vs. high: 1.1±1.7, p=0.63) between low and high motor skills groups. |
| Sääkslahti et al. 1999 [31]; Finland | Cross-sectional | n=105; mean age: 3.75 yr. | Very active indoor play, very active outdoor play, high activity of play activities (indoors and outdoors) on the weekend assessed via proxy-report log. | Fundamental movement skills (walking, running, standing broad jump, agility, throwing at target (2 meter), throwing at target (3 meter), and throwing-catching combination) were objectively measured using the APM-Inventory. | No significant correlations were observed between the play variables and walking, standing broad jump, agility, throwing at target (2 meters), and throwing-catching combination (all r ≤ ±0.16; p≥0.05).  Very active indoor play was significantly correlated with throwing at target (3 meter; r=0.24; p=0.014) but no other measures of fundamental movement skills (all r ≤ ±0.16; p≥0.05).  High level of play activities was significantly correlated with running speed (r=-0.21; p=0.037) but no other measures of fundamental movement skills (all r ≤ ±0.15; p≥0.05). |
| Fisher et al. 2005 [66];  Scotland | Cross-sectional | n=394; mean age: 4.2 yr. | Accelerometer-derived TPA (cpm), LPA (% of time), and MVPA (% of time). | Fundamental movement skills objectively measured using the movement assessment battery. | TPA and MVPA were significantly positively correlated with fundamental movement skills (cpm: r=0.10; p=0.039; MVPA: r=0.18; p<0.01). LPA was not significantly correlated with fundamental movement skills (r=0.02, p=0.625).  TPA (p=0.16) and LPA (p=0.54) were not significantly different across quartiles of fundamental movement skills. MVPA was significantly different across quartiles of fundamental movement skills (p=0.001). |
| Williams et al. 2008 [37]; United States | Cross-sectional | n=198; mean age: 4.2 yr. | Accelerometer-derived LPA, MVPA, VPA (% of time). | Total, locomotor, and objective control motor skills objectively measured using the Children’s Activity and Movement in Preschool Study (CHAMPS) Motor Skill Protocol (CMSP). | No significant difference in LPA between tertiles of total (low: 32.5±0.6; intermediate: 32.6±0.6; high: 32.9±0.6), locomotor (low: 32.6±0.6; intermediate: 31.9±0.6; high: 33.5±0.6), and objective control (low: 32.5±0.6; intermediate: 32.7±0.6; high: 33.0 ±0.7) motor skills (p>0.05).  MVPA (low: 11.4±0.5; intermediate: 12.8±0.5; high: 13.4±0.5) and VPA (low: 3.8±0.3; intermediate: 4.6±0.3; high: 5.0±0.3) were significantly lower in the low tertile of total motor skills compared to intermediate and high tertiles.  MVPA (low: 11.6±0.5; high: 13.4±0.5) and VPA (low: 3.8±0.3; high: 4.9±0.3) were significantly lower in the low tertile of locomotor skills compared to the high tertile. No significant differences were observed in MVPA (low: 11.6±0.5; intermediate: 12.5±0.5) and VPA (low: 3.8±0.3; intermediate: 4.5±0.3) between the low and intermediate tertiles of locomotor skills (p≥0.05).  No significant differences were observed in MVPA (low: 11.8±0.5; intermediate: 12.8±0.5; high: 13.1±0.5) and VPA (low: 4.0±0.3; intermediate: 4.5±0.3; high: 4.8±0.3) between tertiles of object control skills (p≥0.05). |
| Pfeiffer et al. 2009 [41]; United States | Cross-sectional | n=331; mean age: 4.3 yr. | Accelerometer-derived MVPA and TPA (min/hr). | Parental perceptions of children’s coordination (5-point scale) assessed via proxy-report questionnaire. | MVPA was significantly positively correlated with coordination (r=0.16; p≤0.01).  TPA was significantly positively correlated with coordination (r=0.19; p≤0.001). |
| Lin et al. 2016 [42]; Taiwan | Cross-sectional | n=264; mean age: 52.6 mo. | Duration and frequency of physical activity assessed via the proxy-reported Preschool- Aged Children's Physical Activity Questionnaire. | Motor ability and performance objectively measured using the Movement Assessment Battery for Children 2nd edition (MABC-2) and classified as motor difficulty (<5^th^ percentile); at risk for motor difficulty (5-15th percentile); typically developing (>16 percentile). | Participants that engaged in physical activity <5 times/wk were significantly more likely to have a motor difficulty compared to participants who engaged in physical activity >5 times/wk (OR=2.9; 95%CI: 1.5, 5.5).  Participants that engaged in physical activity <840 min/wk were significantly more likely to have a motor difficulty compared to participants who engaged in physical activity >840 min/wk (OR=2.3; 95%CI: 1.0, 5.1). |

AIMS = Alberta Infant Motor Scales; β = standardized beta; CDIIT: Comprehensive Developmental Inventory for Infants and Toddlers; CHAMPS = Children’s Activity and Movement in Preschool Study; CMSP = Children’s Activity and Movement in Preschool Study (CHAMPS) Motor Skill Protocol; cpm = counts per minute; hr = hour; IQR: Interquartile Range; LPA = light-intensity physical activity; MABC-2 = Movement Assessment Battery for Children 2nd edition; min = minute; mo = month; MOT 4-6 = Motoriktestfürvier-bissechsjährige Kinder test battery; MoTB = Motor Test Battery; MVPA = moderate- to vigorous-intensity physical activity; OR = odd ratio; RCT = randomized controlled trial; SD = standard deviation; SPARK: Sports, Play and Active Recreation for Kids; T1-T4 = Time 1 to Time 4; TGMD-2 = Test of Gross Motor Development Version-2; TPA = total physical activity; VPA = vigorous-intensity physical activity; wk = week; yr = year; 95%CI = 95% confidence interval.

*Longitudinal study but only cross-sectional data eligible for this review.

**Table S3.** Summary of studies for psychosocial health

| Refid #. Author (year); country | Study Design | Sample | Exposure | Outcome | Main Findings |
| --- | --- | --- | --- | --- | --- |
| Porter, 1972 [55]; Philippines | RCT | n=130; mean age: 18.3 wk. | Intervention: Planned passive cycling – 2 x 5 min sessions alternated with equal rest, 2 times/day, 6 day/wk, for 2 mo.  Control: Regular child-rearing practices. | Personal-social behavior objectively measured using the Gessell Development Schedules- Development Quotient (units unknown) at baseline, 1 mo follow-up, and 2 mo follow-up. | The intervention group had significant gains (24.07±24.57 at 1 mo follow-up and 41.43±29.85 at 2 mo follow-up) in personal-social behavior compared to the control group (4.92±13.38 at 1 mo follow-up and 17.69±14.95 at 2 mo follow-up).  Note: It is unknown if the intervention resulted in a significant change in physical activity. |
| Lobo & Winsler, 2006 [67]; United States | RCT | n=40; mean age: 40 mo. | Intervention: Dance program – 35 min, 2 times/wk for 8wk.  Control: Regular curriculum play – 35 min, 2 times/wk for 8 wk. | Social competence, internalizing behavior problems and externalizing behavior problems measured via proxy-report Social Competence Behavior Evaluation: Preschool Education Questionnaire (Range 1-6 for each question; higher is better) at baseline and 8 wk follow-up. | Significantly greater mean gains from baseline to 8 wk follow-up were observed in the intervention group for parental-reported social competence (experiment: pre=103.29±38.95, post=139.33±24.27; control: pre=130.16±35.94, post=132.21±33.19) and teacher-reported social competence (experiment: pre=109.09±38.25, post=132.62± 23.32; control: pre=127.84±26.68, post=133.86±25.06), parental-reported internalizing behavior (experiment: pre=68.57±14.67, post=81.38±12.21; control: pre=71.63±11.83, post=73.37±14.80) and teacher-reported internalizing behavior (experiment: pre=73.93±9.87, post=82.57±7.01; control: pre=78.94±8.01, post=83.60±6.48), and parental-reported externalizing behavior (experiment: pre=71.67±15.05, post=81.29±10.49; control: pre=75.37±10.52, post= 76.16±13.85) and teacher-reported externalizing behavior (experiment: pre=81.04±13.35, post=86.43±9.10; control: pre=86.03±7.33, post=87.34±6.52) compared with the control group (p<0.01).  Note: It is unknown if the intervention resulted in a significant change in physical activity. |
| Bonvin et al. 2013 [2]; Switzerland | Clustered RCT | n=1467; mean age: 3.3 yr. | Intervention: Government-led physical activity program (no specific time component or curriculum) for 10 mo.  Control: Regular program | Quality of life assessed via the proxy-reported PedsQL 4.0 (units unknown). | No significant difference in quality of life were observed between the mean individual changes in the intervention group (baseline: 83.0±9.1; follow-up: 82.9±9.3) and the control group (baseline: 82.0±11.0; follow-up: 81.5±11.3).  Note: No significant difference in TPA (cpm) were observed between the mean individual changes in the intervention group (baseline: 620 ±278; follow-up: 765±340) and the control group (baseline: 600 ±206; follow-up: 711±219). No significant differences in MVPA (epochs/hr) were observed between the mean individual changes in the intervention group (baseline: 29.2±14; follow-up: 37.2±17.1) and the control group (baseline: 28.1±12.5; follow-up: 35.9±13.7). No significant differences in VPA (epochs/hr) were observed between the mean individual changes in the intervention group (baseline: 8.1±6.1; follow-up: 10.3±7.5) and the control group (baseline: 7.4±5.3; follow-up: 9.2±6.2). |
| Wang et al. 2008 [68]; Japan | Longitudinal | n=7289; mean age (baseline): 3.43 yr. | Physical activity (active, average, less active then peers) assessed via proxy-report questionnaire at baseline. | Quality of life (good vs. poor) measured via the self-reported Dartmouth Primary Care Cooperative Project (COOP charts) in the first year of junior high school. | Children who were less active compared to those that were active at baseline were significantly more likely to have poor quality of life in the first year of junior high school (OR=1.51; 95%CI: 1.08, 2.11).  Children who were average compared to those that were active at baseline were not significantly more likely to have poor quality of life in the first year of junior high school (OR=1.09; 95%CI: 0.99, 1.20; p=0.073).  However, a significant trend was observed for poor quality of life when moving from the active to less active groups (p for trend=0.010). |
| Vella et al. 2015 [69]; Australia | Longitudinal | n=2700; age range (baseline): 4-5 yr. | TPA (hr/wk) assessed via proxy-report log at baseline.  Sport participation (yes/no) based on swimming, dancing, gymnastics, team sports at baseline. | Health-related quality of life assessed via the proxy-reported PedsQL 4.0 (units unknown). It was completed at baseline and every 24 mo until age 12-13 yr. | Children that participated in sport at baseline were significantly less likely to be in the high risk (continuous decrease; OR=0.52; 95%CI: 0.36, 0.75) and recovery (extremely low at baseline and increased overtime; OR=0.37; 95%CI: 0.17, 0.82) trajectories but not the rebound trajectory (decreases followed by increases; OR=0.66; 95%CI: 0.42, 1.05), compared to the healthy trajectory (consistent high levels).  Higher hr/wk of TPA at baseline was not associated with being in the high risk (OR=0.89; 95%CI: 0.70, 1.22), rebound (OR=0.83; 95%CI: 0.66, 1.06), or recovery (OR=0.60; 95%CI: 0.80, 1.42) quality of life trajectories compared to the healthy trajectory. |
| Matheny & Brown, 1971 [65]; United States | Cross-sectional* | n=112 (56 pairs of twins); age 12 mo. | Activity (units unknown) assessed via proxy-report interview. | Temper frequency (units unknown) assessed via proxy-report interview. | Amount of activity was significantly positively associated with temper frequency (χ^2^=24.12; p<0.01). |
| Irwin et al. 2015 [70]; Canada | Cross-sectional | n=216; age range: 2.5-5 yr. | Accelerometer-derived LMVPA (LPA, MPA, VPA; min/hr), and MVPA (min/hr) during childcare. | Sociability, emotionality, and soothability (units unknown) assessed via the proxy-report Child Temperament Questionnaire (CTQ) | No significant correlations were observed between LMVPA with sociability (r=-0.01), emotionality (r=0.09), soothability (r=-0.07).  No significant correlations were observed between MVPA with sociability (r=-0.01), emotionality (r=0.03), soothability (r=-0.01). |
| Yu et al. 2012 [71]; Australia | Cross-sectional | n=4936; age range: 4-5 yr. | MVPA, walk, bike, other exercise (hr/day) assessed via proxy-report log. | Conduct problems assessed via the proxy-report Strengths and Difficulties Questionnaire (SDQ). | Children at risk of conduct problems compared to children not at risk of conduct problems had significantly higher hr/day of MVPA on the weekday (1.08 vs. 1.07) and weekend (1.66 vs. 1.62).  Children at risk of conduct problems compared to children not at risk of conduct problems had significantly higher hr/day of biking on the weekday (0.25 vs. 0.19) and weekend (0.34 vs. 0.31).  No significant differences were observed between children at risk of conduct problems compared to children not at risk of conduct problems for walking (weekday: 0.18 vs. 0.18; weekend: 0.20 vs. 0.23) and other exercise (weekday: 0.66 vs. 0.69; weekend: 1.13 vs. 1.08) on the weekday or weekend (p≥0.05). |
| Yu et al. 2010 [72];  Australia | Cross-sectional | n=1414; age range: 4-5 yr. | MVPA (walk, bike, exercise; hr/day) assessed via proxy-report log. | Conduct problems assessed via the proxy-report Strengths and Difficulties Questionnaire (SDQ). | *Boys*: Higher bike riding during the weekday was significantly associated with higher odds of being at risk of conduct problems (OR=1.70, SE=0.42). No significant associations were observed between walking on weekdays and risk of conduct problems (OR=0.94, SE=0.23). Additionally, compared to no exercise on weekdays, >0 and ≤1 hr (OR=1.10, SE = 0.29) and >1 hr and ≤24 hr (OR=0.58, SE=0.17) exercise on weekdays was not significantly associated with risk of conduct problems.  No significant associations were observed between walking (OR=0.62, SE=0.18) and riding a bike (OR=0.89, SE=0.21) on weekend days and the risk of conduct problems. In addition, compared to engaging in 0 hr exercise during weekend days, engaging in exercise for >0 and ≤1hr (OR=0.89, SE=0.28) and >1 and ≤2hr (OR=1.02, SE=0.29) were not significantly associated with the risk of conduct problems; however, engaging in exercise for >2 and ≤24hr was associated with higher odds of being at risk of conduct problems (OR=2.23, SE=0.65).  *Girls*: Walking, bike riding, and exercise on weekdays (walking: OR=0.67, SE=0.22; biking: OR=1.11, SE=0.31; exercise for >0 and ≤1hr vs. 0hr: OR=1.13, SE=0.31); exercise for >1 and ≤24hr vs. 0hr: OR=1.00, SE=0.29) and weekend days (walking: OR=1.35, SE=0.38; biking: OR=0.91, SE=0.23; exercise for >0 and ≤1hr vs. 0hr: OR=1.22, SE=0.36); exercise for >1 and ≤2hr vs. 0hr: OR=0.88, SE=0.29; exercise for >2 and ≤24hr vs. 0hr: OR=1.13, SE=0.38) were not significantly associated with the risk of conduct problems (p≥0.05). |
| Fliek et al. 2015 [73]; Belgium | Cross-sectional | n=105; mean age: 4.27 yr. | Rough and tumble play (Range 0-108) assessed via proxy-report questionnaire complete by mother and father. | Anxiety symptoms (Range 0-120) assessed via the proxy-report Preschool Anxiety Scale-Revised questionnaire. | Rough and tumble play was not significantly associated with anxiety symptoms in children as reported by the mother (β= −0.66; SE=0.65) or father β=0.57; SE=0.50). |
| Lindsay & Brussoni, 2014 [74]; United States | Cross-sectional | n=148; mean age: 57.61 mo. | Exercise play (toy-mediated and non-mediated; proportion of time) and rough and tumble play (chasing and play fighting; proportion of time) objective measured using direct observation. | Children's classroom peer acceptance measured via proxy-report sociometric interviews (higher score = greater peer acceptance). | *Mixed-gender peer play:* Toy mediated exercise play was significantly correlated with peer acceptance (r=0.27; p<0.001). No other correlations were observed between play variables and peer acceptance (all r< ±0.15).  *Same-gender peer play:* Toy mediated exercise play (r=0.36; p<0.001) and non-mediated exercise play (r=0.25; p<0.01) were significantly correlated with peer acceptance. No significant correlations were observed between rough and tumble play and peer acceptance (all r< ±0.17).  *Other-gender peer play:* No significant correlations were observed between exercise play, rough and tumble play and peer acceptance (all r< ±0.23). |

β = standardized beta; COOP = Dartmouth Primary Care Cooperative Project; cpm = counts per minute; CTQ = Child Temperament Questionnaire; hr = hour; LMVPA = light- to vigorous-intensity physical activity; LPA = light-intensity physical activity; min = minute; mo = month; MVPA = moderate- to vigorous-intensity physical activity; OR = odd ratio; PedsQL: Pediatric Quality of Life Inventory; RCT = randomized controlled trial; SE = standard error; SDQ = Strengths and Difficulties Questionnaire; TPA = total physical activity; VPA = vigorous-intensity physical activity; wk = week; yr = year; 95%CI = 95% confidence interval.

*Longitudinal study but only cross-sectional data eligible for this review.

**Table S4.** Summary of studies for cognitive development

| Refid #. Author (year); country | Study Design | Sample | Exposure | Outcome | Main Findings |
| --- | --- | --- | --- | --- | --- |
| Porter, 1972 [55]; Philippines | RCT | n=130; mean age: 18.3 wk. | Intervention: Planned passive cycling - 2x5 min sessions alternated with equal rest, 2 times/day, 6 day/wk, for 2 mo.  Control: Regular child-rearing practices. | Language development objectively measured using the Gessell Development Schedules- Development Quotient (units unknown) at baseline, 1 mo follow-up, and 2 mo follow-up. | The intervention group had significant gains (18.33±25.60 at 1 mo follow-up and 33.74±31.85 at 2 mo follow-up) in language development compared to the control group (5.23±16.16 at 1 mo follow-up and 17.43±18.78 at 2 mo follow-up).  Note: It is unknown if the intervention resulted in a significant change in physical activity. |
| Teixeira Costa et al. 2015 [56]; Portugal | RCT | n=324; age range: 3-5 yr. | Intervention: Structured physical education program – 45 min, 2 times/wk for 24 wk.  Control: Standard program of preschool education that did include some physical activity but not structured. | Psychomotor skills (body scheme, spatial organization, temporal organization; units unknown) objectively measured at baseline and 24 wk follow-up. | There was a significant group (intervention vs. control) by test (baseline and 24 wk follow-up) interaction for body scheme, spatial organization, and temporal organization (all p<0.001) that showed increases in the psychomotor skills were significantly larger in the experimental group compared to the control group.  Body Scheme: 3 year olds (intervention group mean difference: 3.13; control group mean difference: 0.73); 4 year olds (intervention group mean difference: 2.42; control group mean difference: 0.40); 5 year olds (intervention group mean difference: 2.95; control group mean difference: 0.75).  Spatial Organization: 3 year olds (intervention group mean difference: 2.91; control group mean difference: 0.82); 4 year olds (intervention group mean difference: 2.51; control group mean difference: 0.46); 5 year olds (intervention group mean difference: 3.07; control group mean difference: 0.63).  Temporal Organization: 3 year olds (intervention group mean difference: 3.49; control group mean difference: 1.41); 4 year olds (intervention group mean difference: 3.13; control group mean difference: 0.58); 5 year olds (intervention group mean difference: 2.90; control group mean difference: 0.08).  Note: It is unknown if the intervention resulted in a significant change in physical activity. |
| Mavilidi et al. 2015 [75]; Australia | Clustered RCT | n=125; mean age: 4.94 yr. | Children were in 1 of 4 conditions over 4 wk (2 days/wk, 15 min/day).  Integrated physical exercise condition: physical exercises to enact the meaning of Italian words.  Non-integrated physical exercise condition: physical exercises at the same intensity during learning but unrelated to the Italian words.  Gesturing condition: enacting the meaning of Italian words while sitting.  Conventional condition: verbal repetition of Italian words while sitting. | Free and cued recalls of the previously learned Italian words (range 0-14) objectively measured at 2, 4, and 10 wk follow-up. | On average, across the three measurement time points, the integrated group (1.73±1.34) freely recalled a significantly greater amount of words than the non-integrated (0.97±1.01, p=0.006), gesturing (1.24±1.35, p=0.049), and conventional (0.77±0.90, p<0.001) groups. Significant differences did not exist between the non-integrated, gesturing, and conventional groups.  On average, across the three measurement time points, the integrated group (4.00±2.25) correctly recalled on cue a significantly greater number of words than the gesture (3.17±2.04, p=0.044) and conventional (2.09±1.49, p<=0.001) groups but not the non-integrated group (3.20±1.55). The non-integrated group recalled more mean words than the conventional group but not the gesturing group. The gesturing group recalled more words than the conventional group.  *Note:* Accelerometer derived TPA (cpm) and MVPA (min) did not significantly differ between the integrated (TPA=938.07±552.76; MVPA=3.85±3.20) and non-integrated (TPA=807.56±552.76; MVPA=3.47±3.40) groups and between the gesturing (TPA=517.57±348.89; MVPA=1.92±1.68) and the conventional (TPA=534.66±311.32; MVPA=1.75±1.17) groups. However, significant differences in TPA and MVPA were observed between both the integrated and non-integrated groups and both the gesturing and conventional groups. |
| Kirk et al. 2014 [76]; United States | Non-randomized Intervention | n=72; mean age (baseline): 3.8 yr. | Intervention: Academic lessons taught by classroom teachers to promote 30 min of MPA/day (2 -15 min lessons) over 6 mo.  Control: Regular classroom instruction without the  academic lessons taught using physical activity. | Early literacy and language skills (Picture-naming assessment of oral language and  Vocabulary (range -=50), rhyming assessment of phonological  awareness (number per 2 min), and alliteration assessment of phonological  awareness (number per 2 min)) were measured using the Early Literacy Individual Growth and Development Indicators (IGDI) objectively measured at baseline, 3 mo follow-up, and 6 mo follow-up. | In the intervention group but not the control group, alliteration was significantly higher at 3 mo follow-up (1.0±0.3) and 6 mo follow-up (1.0±0.3) compared to baseline (0.3±0.1; p<0.05).  Alliteration was also significantly higher in the intervention compared to the control group at 3 mo follow-up (Intervention: 1.0 ± 0.3; Control 0.5±0.3) and 6 mo follow-up (Intervention: 1.0±0.3; Control: 0.5±0.3; p<0.01).  In the intervention group but not the control group, picture naming was significantly higher at 3 mo (21.7±3.3) and 6 mo (24.8±3.4) compared to baseline (17.2±3.5; p<0.01).  Picture naming was also significantly higher in the intervention compared to the control group at 3 mo follow-up (intervention: 21.7±3.3; control 18.5±2.3) and 6 mo follow-up (intervention: 24.8±3.4; control: 19.3±2.3; p<0.01).  In the intervention group, rhyming was not significantly higher at 3 mo follow-up (4.3±1.4) and 6 mo follow-up (4.3±1.6) compared to baseline (4.0±1.5; p≥0.05).  Rhyming was not significantly higher in the intervention compared to the control group at 3 mo follow-up (Intervention: 4.3±1.4; Control 4.0±1.5) and 6 mo follow-up (Intervention: 4.3±1.6; Control: 4.2±1.4; p≥0.05).  Note: Physical activity during free play at pre-school was significantly higher in the intervention group compared to the control group at 3 mo (intervention: 16.1%; control: -2.2%) and 6 mo (intervention: 28.1%; control: -7.2%; p<0.05). |
| Kirk & Kirk 2016 [77]; United States | Non-randomized Intervention | n=54; mean age (baseline): 4.1 yr. | Intervention: Academic lessons taught by classroom teachers to promote 60 min of MVPA/day (2 -30 min lessons) over 8 mo.  Control: Followed Head Start curriculum. | Early literacy and language skills (Picture-naming assessment of oral language and  vocabulary (range -=50), rhyming assessment of phonological  Awareness (number per 2 min), and alliteration assessment of phonological  Awareness (number per 2 min)) were measured using the Early Literacy Individual Growth and Development Indicators (IGDI) objectively measured at baseline, 4 mo follow-up, and 8 mo follow-up. | Significant between groups differences were observed in improvement of rhyming at 8 mo (intervention: 173 ± 12%; control: 28±8 %; p<0.01).  Significant between groups differences in improvement in alliteration was observed at 8 mo (intervention: 52±16%; control: 13±5%; p<0.01).  No significant between groups differences in improvement in picture naming was observed at 8 mo (intervention: 30± 8 %; control: 25±6%; p>0.05).  Note: PA was significantly higher in the intervention group compared to the control group at 4 mo (intervention: 3.5±0.4 vs. control: 2.5±0.4) and 8 mo (intervention: 3.5±0.4 vs. control: 2.6±0.5; higher score indicates higher intensity). |
| Zachopoulou et al. 2006 [78]; United States | Non-randomized Intervention | n=251; mean age: 4.3 yr. | Intervention: Physical education program – 35-40 min 2 times/wk for 10 wk. | Children’s creativity (fluency (range unknown) and imagination (range 1-5)) objectively measured via direct observation using the Thinking Creatively in Action and Movement (TCAM) test at baseline and 10 wk follow-up. | Mean fluency was significantly higher at 10 wk follow-up (21.57±11.77) compared to baseline (13.56±13.56; p<0.05).  Mean imagination was significantly higher at 10 wk follow-up (17.82±6.23) versus baseline (12.41±5.84; p<0.05).  Note: It is unknown if the intervention resulted in a significant change in physical activity. |
| Draper et al. 2012 [58]; South Africa | Non-randomized Intervention | n=83; mean age (intervention): 57 mo; (control): 56 mo. | Intervention: Supervised free play to structured activities for 3-4 sessions/wk, 45-60 min/session for 8 mo.  Control: Usual care. | Cognitive function objectively measured via the Herbst Test (5-point scale: very low to very high) at baseline and 7 mo follow-up. | No significant differences existed in cognitive function between intervention and control groups (p≥0.05). However, only one of the intervention centers participated in the program on a regular basis (called active intervention). An ANOVA comparing the children from the active intervention center compared to children that did not participate or regularly participate indicated that the children from the active intervention school performed better than the children from the other schools (p=0.001).  *Note:* It is unknown if the intervention resulted in a significant change in physical activity. |
| Webster et al. 2015 [79]; United States | Cross-over Trial | n=139; mean age (baseline): 3.80 yr. | Intervention condition: 2 days of a 10 min MVPA activity break.  Control condition: 2 days of typical instruction. | Time on task (before active break, following active break, before typical instruction, following typical instruction) objectively measured via direct observation using a momentary time sampling protocol. | A significant interaction effect was observed between condition and time on task (pre-activity break: 65.30±19.02 %; post-activity break: 81.95±15.11 %; pre-typical instruction: 71.23±18.84 %; post-typical instruction: 77.38±17.03 %; p<0.001).  Note: MVPA was significantly higher during the 10 min break (3.16 min) compared to the same time period on typical instruction days (0.07 min; p<0.001). For the whole school day, children engaged in significantly higher MVPA on activity break days (9.01 min/day) compared to the typical instruction days (7.56 min/day; p=0.01). |
| Palmer et al. 2013 [80];  United States | Cross-over Trial | n=16; mean age: 49.4 mo. | Physical activity condition: One 30 min planned movement session.  Sedentary condition: One 30 min sedentary behavior session. | Sustained attention and response inhibition (units unknown) objectively measured using the Picture Deletion Task for Preschoolers (PDTP). | Significantly fewer failures for sustained attention in the physical activity condition (25.6±12.3) compared to the sedentary condition (44.3±28.7).  No significant difference for response inhibition. However, the results trended in the predicted direction such that children made fewer errors after the exercise condition (4.88±4.43) than after the sedentary condition (10.6±17.2).    *Note:* It is unknown if there were significant differences in physical activity between conditions. |
| Holmes et al. 2006 [81]; United States | Cross-over Trial | n=27; mean age: 55.56 mo. | Condition 1: 10 min outdoor recess.  Condition 2: 20 min outdoor recess.  Condition 3: 30 min outdoor recess. | Post recess attention (0=inattentive; 1 = attentive) objectively measured via direct observation in 30 sec intervals. | Post recess attention was higher after sustained period of outdoor recess.  Mean post recess attention was significantly different across conditions (10 min recess: 0.79±0.15; 20 min recess: 0.79±0.19; 30 min recess: 0.32±0.43; p<0.001).  Note: A post hoc analysis was not conducted to determine where the significant differences between conditions in post recess attention existed. It is also unknown if there were significant differences in physical activity between conditions. |
| Matheny & Brown, 1971 [65]; United States | Cross-sectional* | n=112 (56 pairs of twins); age 12 mo. | Activity (units unknown) assessed via proxy-report interview. | Attention span (units unknown) measured via proxy-report interview. | Amount of activity was significantly negatively associated with attention span (χ^2^=22.14; p<0.001). |
| Irwin et al. 2015 [70]; Canada | Cross-sectional | n=216; age range: 2.5-5 yr. | Accelerometer-derived LMVPA (min/hr) and MVPA (min/hr) during childcare. | Attention span (units unknown) measured via the proxy-report Child Temperament Questionnaire (CTQ). | No significant correlations were observed between LMVPA (r=0.13) and MVPA (r=0.01) and attention span (p≥0.05). |
| Ansari 2015 [30]; United States | Cross-sectional* | n=2810; mean age: 45 mo. | Outdoor play (min/day) at child care assessed via proxy-report questionnaire. | Literacy skills (letter word identification, spelling skills, receptive vocabulary; units unknown) measured with the Woodcock Johnson, Peabody Picture Vocabulary Test via self-report interview.  Math skills (units unknown) measured with the Woodcock Johnson Applied Problems subscale via self-report interview. | Outdoor play at child care was not significantly associated with children’s literacy skills (β=0.01-0.03; 95% CI: -0.05, 0.07) or math skills (β=0.02; 95% CI: -0.03, 0.07). |

β = standardized beta; cpm = counts per minute; CTQ = Child Temperament Questionnaire; hr = hour; IGDI = Individual Growth and Development Indicators; LMVPA = light- to vigorous-intensity physical activity; LPA = light-intensity physical activity; min = minute; mo = month; MVPA = moderate- to vigorous-intensity physical activity; OR = odd ratio; PDTP = Picture Deletion Task for Preschoolers; RCT = randomized controlled trial; TCAM = Thinking Creatively in Action and Movement; TPA = total physical activity; VPA = vigorous-intensity physical activity; wk = week; yr = year; 95%CI = 95% confidence interval.

*Longitudinal study but only cross-sectional data eligible for this review.

**Table S5.** Summary of included studies for fitness

| Refid #. Author (year); country | Study Design | Sample | Exposure | Outcome | Main Findings |
| --- | --- | --- | --- | --- | --- |
| DuRant et al. 1994 [10]; United States | Longitudinal | n=123; mean age (baseline): 4.04 yr. | Physical activity objectively measured with heart rate monitors (% of heart rates 25% and 50% above resting heart rate, mean heart rate) and directly observed with the Children’s Activity Rating Scale (average, % of min at level 3, 4, or 5, and 4 or 5) between baseline and 1 yr follow-up. | Cardiorespiratory fitness objectively measured via a treadmill test at baseline and 1 yr follow-up. | The % of heart rate 25% (r=-0.22; p ≤0.04) and 50% (r=-0.23; p ≤0.03) above resting heart rate was significantly correlated with the heart rate during last 15 seconds of treadmill test (time points unknown). |
| Leppänen et al. 2016 [45]; Sweden | Cross-sectional | n=307; mean age: 4.48 yr. | Accelerometer-derived LPA (min/day), MPA (min/day), VPA (min/day), MVPA (min/day), and 25th, 50th, 75th, 90th and 95th percentiles of 10-s sum of vector magnitude. | Physical fitness (Cardiorespiratory fitness: 20-meter shuttle run (laps); muscular fitness: handgrip strength (kg), standing long jump (cm), and speed-agility: 4x10 shuttle run (seconds)) objectively measured using the PREFIT fitness test battery. | The 95^th^ percentile vector magnitude was significantly associated with all fitness measures (20-meter shuttle run: β=0.27; 95%CI: 0.17, 0.36; handgrip: β=0.09; 95%CI: 0.03, 0.15; standing long jump: β=1.01; 95%CI: 0.43, 1.60; 4x10 shuttle run: β= −0.18; 95%CI: −0.26, −0.11).  The 90th percentile vector magnitude was significantly associated with all fitness measures (20-meter shuttle run: β=0.31; 95%CI: 0.17, 0.45; handgrip: β=0.13; 95%CI: 0.05, 0.22; standing long jump: β=1.25; 95%CI: 0.39, 2.10; 4x10 shuttle run: β= −0.23; 95%CI: −0.33, −0.12).  The 75th percentile vector magnitude was significantly associated with all fitness measures (20-meter shuttle run: β=0.25; 95%CI: 0.03, 0.47; handgrip: β=0.17; 95%CI: 0.04, 0.29; 4x10 shuttle run: β= −0.19; 95%CI: −0.35, −-0.03), except standing long jump (β=1.05; 95%CI: -0.22, 2.32).  VPA was significantly associated with all fitness measures (20-meter shuttle run: β=0.96; 95%CI: 0.65, 1.28; handgrip: β=0.20; 95%CI: 0.01, 0.39; standing long jump: β=3.17; 95%CI: 1.25, 5.10; 4x10 shuttle run: β= −0.67; 95%CI: −0.90, −0.43).  MVPA was significantly associated with all fitness measures (20-meter shuttle run: β=0.24; 95%CI: 0.13, 0.36; standing long jump: β=1.23; 95%CI: 0.55, 1.90; 4x10 shuttle run: β= −0.16; 95%CI: −0.25, −0.08), except handgrip (β=0.07; 95%CI: −0.00, 0.13).  No significant associations were observed between the 25^th^ and 50^th^ percentiles of vector magnitude or LPA, and MPA and any fitness measure (data not shown). |
| Kolpakov et al. 2011 [82]; Russia | Cross-sectional | n=287; preschool age (mean or range not provided). | Habitual physical activity (low, medium, high) objectively assessed with a pedometer and subjectively assessed with a log. | Physical working capacity objectively measured using the Ruffier’s  test with calculation of the Ruffier–Dickson index (RDI; lower number is better). | *Boys:* Average physical work capacity was significantly higher in the low habitual physical activity group (7.52 ±0.28) compared to the mean values of the sample (6.39±6.39; p<0.05).  Average physical work capacity in the moderate (6.13±0.21) and high (5.96±0.27) habitual physical activity groups was significantly lower than the low habitual physical activity group (p<0.05).  *Girls:* Average physical work capacity was significantly higher in the low habitual physical activity group (7.74±0.28) compared to the mean values of the sample (6.71±0.18; p<0.05).  Average physical work capacity in the moderate (6.26±0.19) and high (6.23±0.25) habitual physical activity groups was significantly lower than the low habitual physical activity group (p<0.05). |

β = standardized beta; cpm = counts per minute; hr = hours; LPA = light-intensity physical activity; min = minutes; mo = month; MVPA = moderate- to vigorous-intensity physical activity; RCT = randomized controlled trial; VPA = vigorous-intensity physical activity; wk = week; yr = year; 95%CI = 95% confidence interval.

**Table S6.** Summary of included studies for bone and skeletal health

| Refid #. Author (year); country | Study Design | Sample | Exposure | Outcome | Main Findings |
| --- | --- | --- | --- | --- | --- |
| Specker et al. 1999 [83]; United States | RCT | n=72; age (baseline): 6 mo. | Intervention: Gross motor activity program – 15-20 min/day, 5 days/wk for 1 yr.  Control: Fine motor activity program – 15-20 min/day, 5 days/wk for 1 yr. | Total bone mineral content objectively measured using DXA at ages 6, 9, 12, 15, 18 mo. | There were no significant differences between bone mineral content in the fine and gross motor groups over the study period (data not shown; p≥0.05).  Note: There were no significant differences between groups in accelerometer-derived TPA (counts/hr) at ages 6, 9, 12, 15, 18 mo (p≥0.05). |
| Xu et al. 2013 [84]; China | Cross-sectional | n=11898; mean age: 9.27 mo. | Outdoor activity (hr/day) and physical activity (hr/day) assessed via proxy-report interview. | Bone mineral density (g/cm^2^) of the lumbar spine (L2-L4) objectively measured using DXA. | Outdoor activity (β=0.0068; p=0.0020) and physical activity (β=0.00129; p=0.0156) were significantly associated with bone mineral density. |
| Jazar et al. 2011 [85]; Jordan | Cross-sectional | n=200; mean age (toddler boys): 23.7mo; mean age (toddler girls): 23.47 mo; mean age (preschool boys): 55.4 mo; mean age (preschool girls): 57.12 mo. | Duration (min/day) and frequency (times/mo) of outdoor physical activity. | Vitamin D (25-(OH)-vitamin D_3_) objectively measured in serum. | Vitamin D levels were significantly different between duration of outdoor physical activity groups (p<0.05). Vitamin D levels were lower for children who spent <15 min (18.2±1.6 ng/mL) and ≥15-30 (24.6±1.4 ng/mL) but not >30-60 min (27.8±0.9 ng/mL) in outdoor physical activity compared to children who spent >60 min (28.4±1.1 ng/mL; p<0.05).  Vitamin D levels were significantly different between monthly frequency of outdoor physical activity groups (p<0.05). Vitamin D levels were significantly lower in children who participated in none (15.6±1.4 ng/mL), 1-5 (15.3±1.1 ng/mL), 6-10 (23.8±1.4 ng/mL), 11-15 (24.0±1.6 ng/mL), 16-20 (25.1±1.0 ng/mL) but not 21-25 (30.1±1.1 ng/mL) times/mo of outdoor physical activity compared to children who participated in 26-31 (33.0±1.5 ng/mL) times/mo. |
| Kensarah et al. 2015 [86]; Saudi Arabia | Cross-sectional | n=503; mean age: 38.27 mo. | Outdoor physical activity (min/day and day/mo) assessed via proxy-report interview. | Vitamin D (25-(OH)-vitamin D_3_, parathyroid hormone) objectively measured via non-fasting venous blood samples. | Outdoor physical activity was significantly correlated with vitamin D (r=0.381; p=0.011).  Children who engaged in no days of outdoor physical activity compared to those that engaged in 26-31 days were significantly more likely to have low Vitamin D status (OR=2.44; 95%CI: 0.93, 14.12; p<0.001). Children who engaged in 1-6 days (OR=1.4; 95%CI: 0.88, 5.21), 7-12 days (OR=1.34; 95%CI: 0.91, 2.45), 13-19 days (OR=2.25; 95%CI: 0.78, 7.69), and 20-25 days (1.01; 95%CI: 0.29, 3.31) did not have significantly different Vitamin D status compared to those that engaged in 26-31 days of outdoor physical activity. |
| Specker et al. 2001 [87]; United States | Cross-sectional | n=239; mean age (boys): 4.0 yr; mean age (girls): 3.9 yr. | Accelerometer-derived TPA (counts per day X 10,000), MVPA (% of time), VPA (% of time). | Total body bone area (cm^2^), total body bone mineral content (g), periosteal circumference of tibia (mm), endosteal circumference of tibia (mm), and cortical bone area of tibia (mm^2^) objectively measured using DXA. | No significant association was observed between any of the physical activity measures and any of the bone measures (data not provided). |
| Harvey et al. 2012 [88]; England | Cross-sectional | n=422; mean age: 4.1 yr. | Accelerometer-derived MVPA (min/day). | Hip bone area (cm^2^), hip bone mineral content (g), areal bone mineral density (grams/cm^2^), estimated volumetric bone mineral density (units unknown) objectively measuring using DXA. | MVPA was significantly positively associated with hip bone area (R^2^=0.03, p<0.001), hip bone mineral content (R^2^=0.04, p<0.001), areal bone mineral density (R^2^=0.03, p=0.001), and estimated volumetric bone mineral density (R^2^=0.02, p <0.01). |
| Herrmann et al. 2015 [89]; Sweden, Germany, Hungary, Italy, Cyprus, Spain, Beligium, Estonia | Cross-sectional | n=1512; mean age: 4.4 yr. | Accelerometer-derived TPA (per 100 cpm), LPA (hr/day), MPA (per 10 min/day), VPA (per 10min/day), MVPA (per 10 min/day).  Leisure time physical activity (outdoor and sport; hr/wk) and weight bearing activity (based on sport-based physical activity; moderate/high and low/no). | Bone stiffness (index) objectively measured using quantitative ultrasound. | LPA was not significantly associated with bone stiffness (β=-0.47; p=0.26).  TPA (β=0.92; p<0.001), MPA (β=0.75; p=0.003), MVPA (β=0.58; p=0.003), and leisure time physical activity (β=0.07; p=0.047) were significantly associated with bone stiffness but VPA was not (β=1.22; p=0.05).  Engaging in weight bearing activity compared to not engaging in weight bearing activity was significantly associated with higher mean bone stiffness (β=2.57; p=0.003). |

β = standardized beta; cm = centimeter; cpm = counts per minute; DXA = dual-energy X-ray absorptiometry; g = gram; hr = hours; L2-L4 = lumber spines 2-4; LPA = light-intensity physical activity; min = minutes; mm = millimeter; mo = month; MVPA = moderate- to vigorous-intensity physical activity; OR = odd ratio; OSRAP = the observation system for recording activity in preschool; RCT = randomized controlled trial; TPA = total physical activity; VPA = vigorous-intensity physical activity; wk = week; yr = year; 95%CI = 95% confidence interval.

**Table S7**. Summary of studies for cardiometabolic health

| Refid #. Author (year); country | Study Design | Sample | Exposure | Outcome | Main Findings |
| --- | --- | --- | --- | --- | --- |
| Scheffler et al. 2007 [90]; Germany | Non-randomized intervention | n=264; age (baseline): 3 yr. | Intervention: Playful-athletic exercise programme. 1-hr sessions 3times/wk for 24 mo.  Control: Usual programming. | DBP (mm Hg) objectively measured during rest and activity. | Children in the intervention group had significantly lower DBP at 5 yr compared to the controls during activity (intervention: 62.0±11.2; control: 68.8±11.1 mm HG), 1 minute after the activity (intervention: 65.1 ±9.4; control 68.1±9.2 mm Hg) and 3 min after the activity (intervention: 64.4±9.2; control: 67.6±8.3 mm Hg).  Note: It is unknown if the intervention resulted in a significant change in physical activity. |
| DuRant et al. 1994 [10]; United States | Longitudinal | n=123; mean age (baseline): 4.04 yr. | Physical activity objectively measured with heart rate monitor (% of heart rates 25% and 50% above resting heart rate, mean heart rate) and directly observed with the Children’s Activity Rating Scale (average, % of min at level 3, 4, or 5, and 4 or 5) between baseline and 1 yr follow-up. | Total serum cholesterol (mg/dl), triglycerides (mg/dl), HDL (mg/dl), HDL_2_ (mg/dl), LDL (mg/dl), LDL/HDL, total serum cholesterol/HDL objectively measured at 1 yr follow-up. | None of the physical activity measures were significantly correlated with any of the cardiometabolic health indicators (all r< ±0.23; p≥0.05). |
| Wilson et al. 1992 [91]; United States | Cross-sectional and  Longitudinal | n (cross-sectional) = 204;  n (longitudinal) = 168; mean age (baseline): boys, 4.5 yr; girls, 4.4 yr. | Structured, leisure, and aerobic physical activity was measured via parental-report questionnaire at baseline. | SBP (mm Hg) and DBP (mm Hg) objectively measured via Dinamap 1846SX monitor at baseline, 1 yr follow-up, and 2 yr follow-up. | *Cross sectional*:  *Boys:* Structured activity (β= −0.89, SE=1.57), leisure activity (β= −1.06, SE=1.81), and aerobic activity (β=0.88, SE=1.37) were not significantly associated with SBP. Structured activity (β=0.81, SE=1.08), leisure activity (β= −1.40, SE=1.25), and aerobic activity (β=0.03, SE=0.95) were not significantly associated with DBP.    *Girls:* Structured activity (β= −2.13, SE=1.97), leisure activity (β=0.13, SE=1.25), and aerobic activity (β=1.26, SE=1.29) were not significantly associated with SBP. Structured activity (β= −2.79, SE=1.53), leisure activity (β=0.53, SE=0.97), and aerobic activity (β=0.24, SE=1.00) were not significantly associated with DBP.  *Longitudinal*:  *Boys:* Structured activity (β= −2.20, SE=1.43), leisure activity (β=1.65, SE=1.34), and aerobic activity (β= −0.79, SE=1.40) at baseline were not significantly associated with SBP at 1-yr follow up.  Structured activity (β= −1.47, SE=1.20) and aerobic activity (β= −0.93, SE=1.18) at baseline were not significantly associated with DBP at 1-yr follow up. However, leisure activity (β=2.50; SE=1.13) at baseline was significantly associated with DBP at 1-yr follow up.  Structured activity (β= −0.76, SE=1.85), leisure activity (β=0.24, SE=1.52), and aerobic activity (β= −1.10, SE=1.57) at baseline were not significantly associated with SBP at 2-yr follow up.  Structured activity (β=0.49, SE=1.30), leisure activity (β=1.19, SE=1.07) at baseline were not significantly associated with DBP at 2-yr follow up. However, aerobic activity (β= −2.72, SE=1.10) at baseline was significantly associated with DBP at 2-yr follow-up.  Girls: Structured activity (β= −2.76, SE=2.44), leisure activity (β=2.87, SE=1.91), and aerobic activity (β=1.43, SE=1.72) at baseline were not associated with SBP at 1-yr follow up.  Structured activity (β= −0.60, SE=1.88), leisure activity (β=1.92, SE=1.47), and aerobic activity (β=0.91, SE=1.33) at baseline were not associated with DBP at 1-yr follow up.  Structured activity (β=0.23, SE=2.64), leisure activity (β=2.20, SE=2.21), and aerobic activity (β=0.01, SE=1.93) at baseline were not associated with DBP at 2-yr follow up.  Structured activity (β= −0.58, SE=1.76), leisure activity (β=1.29, SE=1.47), and aerobic activity (β= −0.93, SE=1.28) at baseline were not associated with DBP at 2-yr follow up. |
| Kolpakov et al. 2011 [82]; Russia | Cross-sectional | n=287; preschool age (mean or range not provided). | Habitual physical activity (low, medium, high) objectively assessed with a pedometer and subjectively assessed with a log. | SBP (mm Hg) and DBP (mm Hg) objectively measured. | *Boys:* Average SBP in the low habitual physical activity group (93.51±0.51) was significant lower compared to the mean values of the sample (97.32±0.27; p<0.05).  Average SBP in the moderate (96.92±0.31) and high (99.68±0.53) habitual physical activity groups were significantly higher than the low habitual physical activity group (p<0.05).  Average DBP in the low habitual physical activity group (55.32±0.55) was significantly lower compared to the mean values of the sample (57.08±0.27; p<0.05).  Average DBP in the moderate (57.31±0.36) and high (58.15±0.47) habitual physical activity groups were significantly higher than the low habitual physical activity group (p<0.05).  Girls: Average SBP in the low habitual physical activity group (92.14±0.53) was significantly lower compared to the mean values of the sample (95.56±0.25; p<0.05).  Average SBP in the moderate (95.83±0.36) and high (98.52±0.43) habitual physical activity groups were significantly higher than the low habitual physical activity group (p<0.05).  Average DBP in the low habitual physical activity group (55.04±0.52) was significantly lower compared to the mean values of the sample (56.76±0.21; p<0.05).  Average DBP in the moderate (57.02±0.33) and high (58.04±0.46) habitual physical activity groups were significantly higher than the low habitual physical activity group (p<0.05). |
| Sääkslahti et al. 1999 [31]; Finland | Cross-sectional | n=105; mean age: 3.75 yr. | Very active indoor play, very active outdoor play, high activity of play activities (indoors and outdoors) on the weekend  assessed via proxy-report log. | SBP (mm Hg), DBP (mm Hg), total cholesterol (mmol/L), and HDL (mmol/L) objectively measured. | No significant correlations were observed between play variables and SBP or DBP (all r< ±0.17; p≥0.05).  High activity of play activities was significantly correlated with total cholesterol (r=-0.21; p=0.034). No other significant correlations were observed between play variables and total cholesterol (all r< ±0.17; p≥0.05).  Very active outdoor play was significantly correlated with HDL (r=-0.21; p=0.042). No other significant correlations were observed between play variables and HDL or total cholesterol (all r< ±0.19; p≥0.05). |
| Jiménez-Pavón et al. 2013 [92]; Italy, Estonia, Cyprus, Belgium, Sweden, Germany, Hungary, and Spain | Cross-sectional | n=994; mean age: 4.4 yr. | Accelerometer-derived TPA (cpm), MPA (min/day), VPA (min/day), MVPA (min/day). | Clustered cardiovascular risk score assessed via objectively measures of systolic BP, triglycerides, total cholesterol/HDL, HOMA-IR, sum of two skinfolds. | *Boys:* Participants in Quintile (Q) 1 (but not Q2 (OR=1.59; 95%CI: 0.65, 3.91), Q3 (OR=1.31; 95%CI: 0.55, 3.16), Q4 (OR=2.21; 95%CI: 0.98, 4.98)) compared to Q5 of TPA were significantly more likely to have at risk for cardiovascular disease (OR=2.58; 95%CI: 1.07, 6.18).  Participants in Q1 (OR=1.26; 95%CI: 0.55, 2.88), Q2 (OR=1.27; 95%CI: 0.57, 2.83), Q3 (OR=1.17; 95%CI: 0.51, 2.66), and Q4 (OR=1.23; 95%CI: 0.56, 2.69) compared to Q5 of MPA were not significantly more likely to be at risk for cardiovascular disease.  Participants in Q1 (OR=1.39; 95%CI: 0.56, 3.45), Q2 (OR=2.21; 95%CI: 0.95, 5.15), Q3 (OR=1.98; 95%CI: 0.85, 4.58), and Q4 (OR=1.62; 95%CI: 0.68, 3.85) compared to Q5 of MVPA were not significantly more likely to be at risk for cardiovascular disease.  Participants in Q2 (but not Q1 (OR=1.23; 95%CI: 0.46, 3.26), Q3 (OR=1.98; 95%CI: 0.79, 4.93), Q4 (OR=2.14; 95%CI: 0.89, 5.15)) compared to Q5 of VPA were significantly more likely to be at risk for cardiovascular disease (OR=2.91; 95%CI: 1.25, 6.82).  *Girls:* Participants in Q1 (OR=1.03; 95%CI: 0.43, 2.47), Q2 (OR=0.76; 95%CI: 0.32, 1.85), Q3 (OR=0.81; 95%CI: 0.34, 1.90), and Q4 (OR=0.72; 95%CI: 0.31, 1.63) compared to Q5 of TPA were not significantly more likely to be at risk for cardiovascular disease.  Participants in Q1 (OR=0.77; 95%CI: 0.32, 1.85), Q2 (OR=0.80; 95%CI: 0.33, 1.93), Q3 (OR=0.69; 95%CI: 0.29, 1.67), and Q4 (OR=0.67; 95%CI: 0.28, 1.62) compared to Q5 of MPA were not significantly more likely to be at risk for cardiovascular disease.  Participants in Q1 (OR=2.54; 95%CI: 0.88, 7.29), Q2 (OR=2.71; 95%CI: 0.96, 7.70), Q3 (OR=1.68; 95%CI: 0.55, 5.15), and Q4 (OR=2.29; 95%CI: 0.82, 6.37) compared to Q5 of VPA were not significantly more likely to be at risk for cardiovascular disease.  Participants in Q1 (OR=1.23; 95%CI: 0.48, 3.17), Q2 (OR=1.18; 95%CI: 0.45, 3.08), Q3 (OR=1.26; 95%CI: 0.50, 3.13), and Q4 (OR=1.29; 95%CI: 0.42, 2.98) compared to Q5 of MVPA were not significantly more likely to be at risk for cardiovascular disease. |
| Klesges et al. 1990 [44]; United States | Cross-sectional | n=137; mean age (baseline): 4.44 yr. | Accelerometer-derived TPA (standardized activity score). | SBP, DBP, mean arterial pressure (units not provided) objectively measured. | No significant correlations were observed between TPA and SBP, DBP, mean arterial pressure (all r< ±0.02; p≥0.05). |
| Saakslahti et al. 2004 [52];  Finland | Cross-sectional* | n=155; mean age: 4.9 yr. | High-activity playing (hr/day of indoor and outdoor) assessed via proxy-report log. | SBP (mm Hg), DBP (mm Hg), Total cholesterol (mmol/L), HDL cholesterol (mmol/L), HDL/total, triglycerides objectively measured. | *Boys*: No significant correlations were observed between high-activity playing and cardiometabolic health (all r< ±0.24; p≥0.05).  *Girls*: No significant correlations were observed between high-activity playing and cardiometabolic health (all r< ±0.22; p≥0.05). |

β = standardized beta; BP = blood pressure; cpm = counts per minute; DBP = diastolic blood pressure; HDL = high-density lipoprotein cholesterol; HOMA-IR = Homeostasis model of assessment – Insulin resistance; hr = hour; LPA = light-intensity physical activity; min = minute; mo = month; MVPA = moderate- to vigorous-intensity physical activity; OR = Odd Ratio; OSRAP = the observation system for recording activity in preschool; RCT = randomized controlled trial; SBP = systolic blood pressure; SE = standard error; TPA = total physical activity; VPA = vigorous-intensity physical activity; wk = week; yr = year; 95%CI = 95% confidence interval.

*Longitudinal study but only cross-sectional data eligible for this review.

**Table S8.** Summary of included studies for risks/harm

| Refid #. Author (year); country | Study Design | Sample | Exposure | Outcome | Main Findings |
| --- | --- | --- | --- | --- | --- |
| Damashek & Kuhn, 2012 [93]; United States | Case Cross-over | n=170; mean age: 24 mo. | High or low activity level when child was injured and high and low activity level for control condition (same date and time one wk earlier) assessed via proxy-report log over 6 mo period. | Day, time, and type of injury assessed via proxy-report log over 6 mo period.  Injury severity assessed via the minor injury severity scale. | When children were engaging in high activity level compared to low activity level they were significantly more likely to be injured (OR=13.96; 95%CI: 9.55, 20.40).  Activity level was not significantly associated with injury severity (β=0.10; SE=0.16; p≥0.05). |
| Clark et al. 2008 [94]; England | Longitudinal | n=2692; age (baseline): 4.5 yr. | Outdoor time in summer and winter (hr/wk) assessed via proxy-report questionnaire at age 4.5 yr. | Fracture incidence since last visit (approximately 12-24 mo prior) assessed by self-report interview at age 9-11 yr. | Children who spent ≥28hr/wk outdoors in the summer, compared to <28hr/wk, at age 4.5 yr were significantly more likely to report a fracture between age 9 and 11 yr (OR=2.38; 95%CI: 1.56, 3.62). Children in the middle tertile of outdoor time in the winter, compared to the lowest tertile, at age 4.5 yr were significantly less likely to report a fracture between age 9 and 11 yr (OR=0.53; 95%CI: 0.38, 0.75). Children in the highest tertile of outdoor time in the winter, compared to the lowest tertile, at age 4.5 yr were not significantly less likely to report a fracture between age 9 and 11 yr (OR=0.79; 95%CI: 0.50, 1.25). However, a significant test for trend was observed (OR=0.77; 95%CI: 0.61, 0.97). |
| Hutchison et al. 2003 [95]; New Zealand | Case-control | n=194; age range: 2-12 mo. | Activity level and daily duration of tummy time at assessed at present time and 6 wk of age via proxy-report interview. | Nonsynostotic plagiocephaly objectively assessed by both visual and anthropometric examinations at referring clinics. | Cases were significantly more likely to be very inactive/inactive/average compared to active/very active (OR=3.23; 95%CI: 1.38, 7.56) at present time but not at 6 wk of age (data not shown).  No significant differences between cases and controls in regard to whether tummy time was provided (data not shown).  Cases were significantly more likely to have <5 min/day of tummy time compared to ≥5 min/day at 6 wk of age (OR=2.26; 95%CI: 1.03, 5.00).  Note: It was unclear if there was a significant difference in tummy time between cases and control at present time. |
| van Vlimmeren et al. 2007 [96];  Netherlands | Cross-sectional* | n=380; mean age: 7.4 wk. | Age when first put in prone position and frequency (times/day) and duration (min/day) of tummy time while awake assessed via proxy-report questionnaire or interview (unclear). | Deformational plagiocephaly (oblique diameter difference index ≥104%) assessed via objectively measured head circumference. | Participants whose first age of tummy time was ≥3 wk of age compared to <3 wk of age were not significantly less likely to have deformational plagiocephaly (OR=0.9; 95%CI: 0.55, 1.49).  Participants who engaged in ≤5 min/day of tummy time compared >5 min/day were not significantly more likely to have deformational plagiocephaly (OR=0.9; 95%CI: 0.55, 1.48).  Participants who engaged in <3 times/day of tummy time compared ≥3 times/day were not significantly more likely to have deformational plagiocephaly (OR=2.4; 95%CI: 0.90, 6.20). |

β = standardized beta; hr = hours; mo = month; OR = odd ratio; SE = standard error; wk = week; yr = year; 95%CI = 95% confidence interval.

*Longitudinal study but only cross-sectional data eligible for this review.

**References**

1. de Vries A, Huiting H, Heuvel E, L'abée C, Corpeleijn E, Stolk R. An activity stimulation programme during a child's first year reduces some indicators of adiposity at the age of two‐and‐a‐half. Acta Paediatr. 2015;104(4):414-21.

2. Bonvin A, Barral J, Kakebeeke TH, Kriemler S, Longchamp A, Schindler C, et al. Effect of a governmentally-led physical activity program on motor skills in young children attending child care centers: A cluster randomized controlled trial. Int J Behav Nutr Phys Act. 2013;10:90.

3. Jones RA, Riethmuller A, Hesketh K, Trezise J, Batterham M, Okely AD. Promoting fundamental movement skill development and physical activity in early childhood settings: A cluster randomized controlled trial. Pediatr Exerc Sci. 2011;23(4):600-15.

4. Annesi JJ, Smith AE, Tennant GA. Effects of a cognitive–behaviorally based physical activity treatment for 4- and 5-year-old children attending US preschools. Int J Behav Med. 2013;20(4):562-6.

5. Mo-suwan L, Pongprapai S, Junjana C, Puetpaiboon A. Effects of a controlled trial of a school-based exercise program on the obesity indexes of preschool children. Am J Clin Nutr. 1998;68(5):1006-11.

6. Monsalves Álvarez M, Castro Sepúlveda M, Zapata Lamana R, Rosales Soto G, Salazar G. Motor skills and nutritional status outcomes from a physical activity intervention in short breaks on preschool children conducted by their educators: A pilot study. Nutrición Hospitalaria. 2015;32(4);1576-81.

7. Krombholz H. The impact of a 20-month physical activity intervention in child care centers on motor performance and weight in overweight and healthy-weight preschool children. Percept Mot skill.s 2012;115(3):919-32.

8. Sijtsma A, Sauer PJ, Stolk RP, Corpeleijn E. Infant movement opportunities are related to early growth—GECKO Drenthe cohort. Early Hum Dev. 2013;89(7):457-61.

9. Carter PJ, Taylor BJ, Williams SM, Taylor RW. Longitudinal analysis of sleep in relation to BMI and body fat in children: The FLAME study. BMJ. 2011;342:d2712.

10. DuRant RH, Baranowski T, Rhodes T, Gutin B, Thompson WO, Carroll R, et al. Association among serum lipid and lipoprotein concentrations and physical activity, physical fitness, and body composition in young children. J Pediatr. 1993;123(2):185-92.

11. Klesges RC, Klesges LM, Eck LH, Shelton ML. A longitudinal analysis of accelerated weight gain in preschool children. Pediatr. 1995;95(1):126-30.

12. Butte NF, Puyau MR, Wilson TA, Liu Y, Wong WW, Adolph AL, et al. Role of physical activity and sleep duration in growth and body composition of preschool‐aged children. Obesity. 2016;24(6):1328-35.

13. Huynh DT, Dibley MJ, Sibbritt D, Tran H, Le QT. Influence of contextual and individual level risk factors on adiposity in a preschool child cohort in Ho Chi Minh City, Vietnam. Pediatr Obes. 2011;6(2):e487-500.

14. de Coen V, De Bourdeaudhuij I, Verbestel V, Maes L, Vereecken C. Risk factors for childhood overweight: A 30-month longitudinal study of 3-to 6-year-old children. Public Health Nutr. 2014;17(09):1993-2000.

15. He Q, Ding Z, Fong D, Karlberg J. Risk factors of obesity in preschool children in China: A population-based case-control study. Int J Obes. 2000;24(11):1528-36.

16. Takahashi E, Yoshida K, Sugimori H, Miyakawa M, Izuno T, Yamagami T, et al. Influence factors on the development of obesity in 3-year-old children based on the Toyama study. Prev Med. 1999;28(3):293-6.

17. Kain J, Andrade M. Characteristics of the diet and patterns of physical activity in obese Chilean preschoolers. Nutr Res. 1999;19(2):203-15.

18. Shapiro LR, Crawford PB, Clark MJ, Pearson DL, Raz J, Huenemann RL. Obesity prognosis: A longitudinal study of children from the age of 6 months to 9 years. Am J Public Health. 1984;74(9):968-72.

19. LaRowe TL, Adams AK, Jobe JB, Cronin KA, Vannatter SM, Prince RJ. Dietary intakes and physical activity among preschool-aged children living in rural American Indian communities before a family-based healthy lifestyle intervention. J Am Diete Assoc. 2010;110(7):1049-57.

20. Chen LP, Ziegenfuss JY, Jenkins SM, Beebe TJ, Ytterberg KL. Pediatric obesity and self-reported health behavior information. Clin Pediatr. 2011;50(9):872-5.

21. Johansson E, Hagströmer M, Svensson V, Ek A, Forssén M, Nero H, Marcus C. Objectively measured physical activity in two-year-old children–levels, patterns and correlates. Int J Behav Nutr Phys Act. 2015;12:3.

22. Nelson JA, Carpenter K, Chiasson MA. Diet, activity, and overweight among preschool-age children enrolled in the Special Supplemental Nutrition Program for Women, Infants, and Children (WIC). Prev Chronic Dis. 2006;3(2):1-12.

23. Østbye T, Malhotra R, Stroo M, Lovelady C, Brouwer R, Zucker N, et al. The effect of the home environment on physical activity and dietary intake in preschool children. Int J Obes. 2013;37(10):1314-21.

24. Kagamimori S, Yamagami T, Sokejima S, Numata N, Handa K, Nanri S, et al. The relationship between lifestyle, social characteristics and obesity in 3‐year‐old Japanese children. Child Care Health Dev. 1999;25(3):235-48.

25. Trost SG, Sirard JR, Dowda M, Pfeiffer KA, Pate RR. Physical activity in overweight and nonoverweight preschool children. Int J Obes.2003;27(7):834-9.

26. Lioret S, Maire B, Volatier J, Charles M. Child overweight in France and its relationship with physical activity, sedentary behaviour and socioeconomic status. Eur J Clin Nutr. 2007;61(4):509-16.

27. Kuzik N, Carson V. The association between physical activity, sedentary behavior, sleep, and body mass index z-scores in different settings among toddlers and preschoolers. BMC Pediatr. 2016;16:100.

28. Burdette HL, Whitaker RC. A national study of neighborhood safety, outdoor play, television viewing, and obesity in preschool children. Pediatr. 2005;116(3):657-62.

29. Bonvin A, Barral J, Kakebeeke TH, Kriemler S, Longchamp A, Marques-Vidal P, et al. Weight status and gender-related differences in motor skills and in child care-based physical activity in young children. BMC Pediatr. 2012;12:23.

30. Ansari A, Pettit K, Gershoff E. Combating Obesity in Head Start: Outdoor Play and Change in Children's Body Mass Index. JDBP. 2015;36(8):605-12.

31. Sääkslahti A, Numminen P, Varstala V, Helenius H, Tammi A, Viikari J, et al. Physical activity as a preventive measure for coronary heart disease risk factors in early childhood. Scand J Med Sci Sports. 2004;14(3):143-9.

32. Sijtsma A, Sauer PJ, Corpeleijn E. Parental correlations of physical activity and body mass index in young children-the GECKO Drenthe cohort. Int J Behav Nutr Phys Act. 2015;12:132.

33. Jouret B, Ahluwalia N, Cristini C, Dupuy M, Nègre-Pages L, Grandjean H, et al. Factors associated with overweight in preschool-age children in southwestern France. Am J Clin Nutr. 2007;85(6):1643-9.

34. Sijtsma A, Koller M, Sauer PJ, Corpeleijn E. Television, sleep, outdoor play and BMI in young children: The GECKO Drenthe cohort. Eur J Pediatr. 2015;174(5):631-9.

35. Pallan MJ, Adab P, Sitch AJ, Aveyard P. Are school physical activity characteristics associated with weight status in primary school children? A multilevel cross-sectional analysis of routine surveillance data. Arch Dis Child. 2014;99(2):135-41.

36. Collings PJ, Brage S, Ridgway CL, Harvey NC, Godfrey KM, Inskip HM, et al. Physical activity intensity, sedentary time, and body composition in preschoolers. Am J Clin Nutr. 2013;97(5):1020-8.

37. Williams HG, Pfeiffer KA, O'neill JR, Dowda M, McIver KL, Brown WH, et al. Motor skill performance and physical activity in preschool children. Obesity. 2008;16(6):1421-6.

38. Watanabe E, Lee J, Kawakubo K. Associations of maternal employment and three-generation families with pre-school children's overweight and obesity in Japan. Int J Obes. 2011;35(7):945-52.

39. Hajian-Tilaki K, Heidari B. Childhood obesity, overweight, socio-demographic and life style determinants among preschool children in Babol, Northern Iran. Iran J Public Health. 2013;42(11):1283-91.

40. Jones RA, Okely AD, Gregory P, Cliff DP. Relationships between weight status and child, parent and community characteristics in preschool children. Int J Pediatr Obes. 2009;4(1):54-60.

41. Pfeiffer KA, Dowda M, McIver KL, Pate RR. Factors related to objectively measured physical activity in preschool children. Pediatr Exerc Sci. 2009;21(2):196-208.

42. Lin LY, Cherng RJ, Chen YJ. Relationship between time use in physical activity and gross motor performance of preschool children. Aust Occup Ther J. 2016; doi:10.1111/1440-1630.12318.

43. Jago R, Baranowski T, Baranowski JC, Thompson D, Greaves K. BMI from 3-6 y of age is predicted by TV viewing and physical activity, not diet. Int J Obes. 2005;29(6):557-64.

44. Klesges RC, Haddock CK, Eck LH. A multimethod approach to the measurement of childhood physical activity and its relationship to blood pressure and body weight. J Pediatr. 1990;116(6):888-93.

45. Leppänen M, Nyström CD, Henriksson P, Pomeroy J, Ruiz J, Ortega F, et al. Physical activity intensity, sedentary behavior, body composition and physical fitness in 4-year-old children: Results from the MINISTOP trial. Int J Obes. 2016;40;1126-33.

46. España-Romero V, Mitchell JA, Dowda M, O’Neill JR, Pate RR. Objectively measured sedentary time, physical activity and markers of body fat in preschool children. Pediatr Exerc Sci. 2013;25(1):154-63.

47. Cox R, Skouteris H, Rutherford L, Fuller-Tyszkiewicz M, Hardy LL. Television viewing, television content, food intake, physical activity and body mass index: A cross-sectional study of preschool children aged 2-6 years. Health Promot J Austr 2012;23(1):58-62.

48. Söderström M, Boldemann C, Sahlin U, Mårtensson F, Raustorp A, Blennow M. The quality of the outdoor environment influences childrens health–a cross‐sectional study of preschools. Acta Paediatr. 2013;102(1):83-91.

49. Jiang J, Rosenqvist U, Wang H, Greiner T, Ma Y, Toschke AM. Risk factors for overweight in 2‐to 6‐year‐old children in Beijing, China. Int J Pediatr Obes. 2006; 1(2):103-8.

50. Cardon G, De Bourdeaudhuij I, Iotova V, Latomme J, Socha P, Koletzko B, et al. Health Related Behaviours in Normal Weight and Overweight Preschoolers of a Large Pan-European Sample: The ToyBox-Study. PloS One. 2016;11(3):e0150580.

51. Eijkemans M, Mommers M, de Vries SI, van Buuren S, Stafleu A, Bakker I, et al. Asthmatic symptoms, physical activity, and overweight in young children: A cohort study. Pediatr. 2008;121(3):e666-72.

52. Sääkslahti A, Numminen P, Varstala V, Helenius H, Tammi A, Viikari J, et al. Physical activity as a preventive measure for coronary heart disease risk factors in early childhood. Scand J Med Sci Sports. 2004;14(3):143-9.

53. Anderson SE, Economos CD, Must A. Active play and screen time in US children aged 4 to 11 years in relation to sociodemographic and weight status characteristics: A nationally representative cross-sectional analysis. BMC Public Health. 2008;8:366.

54. de Carvalho Cremm E, Leite FHM, de Abreu DSC, de Oliveira MA, Scagliusi FB, et al. Factors associated with overweight in children living in the neighbourhoods of an urban area of Brazil. Public Health Nutr. 2012;15(6):1056-64.

55. Porter LS. The Impact of physical-physiological activity on infants' growth and development. Nursing Res. 1972;21(3):210-9.

56. Teixeira Costa HJ, Abelairas-Gomez C, Arufe-Giráldez V, Pazos-Couto JM, Barcala-Furelos R. Influence of a physical education plan on psychomotor development profiles of preschool children. J Human Sport Exerc. 2015;10(1):126-40.

57. Mostafavi R, Ziaee V, Akbari H, Haji-Hosseini S. The effects of spark physical education program on fundamental motor skills in 4-6 year-old children. Iran J Pediatr. 2014;23(2): 216-9.

58. Draper CE, Achmat M, Forbes J, Lambert EV. Impact of a community-based programme for motor development on gross motor skills and cognitive function in preschool children from disadvantaged settings. Early Child Dev Care. 2012;182(1):137-52.

59. Livonen S, Sääkslahti A, Nissinen K. The development of fundamental motor skills of four‐to five‐year‐old preschool children and the effects of a preschool physical education curriculum. Early Child Dev Care. 2011;181(3):335-43.

60. Venetsanou F, Kambas A. How can a traditional Greek dances programme affect the motor proficiency of pre‐school children? Research in Dance Education. 2004;5(2):127-38.

61. Sigmundsson H, Hopkins B. Baby swimming: Exploring the effects of early intervention on subsequent motor abilities. Child Care Health Dev. 2010;36(3):428-30.

62. Kuo Y-L, Liao H-F, Chen P-C, Hsieh W-S, Hwang A-W. The influence of wakeful prone positioning on motor development during the early life. J Dev Behav Pediatr. 2008; 29(5):367-76.

63. de Kegel A, Peersman W, Onderbeke K, Baetens T, Dhooge I, et al. New reference values must be established for the Alberta Infant Motor Scales for accurate identification of infants at risk for motor developmental delay in Flanders. Child Care Health Dev. 2013;39(2):260-7.

64. Dudek-Shriber L, Zelazny S: The effects of prone positioning on the quality and acquisition of developmental milestones in four-month-old infants. Pediatr Phys Ther. 2007;19(1):48-55.

65. Matheny AP, Brown AM. Activity, motor coordination and attention: Individual differences in twins. Percept Mot Skills. 1971;32(1):151-8.

66. Fisher A, Reilly JJ, Kelly LA, Montgomery C, Williamson A, Paton JY, et al. Fundamental movement skills and habitual physical activity in young children. Med Sci Sports Exerc. 2005;37(4):684-8.

67. Lobo YB, Winsler A. The effects of a creative dance and movement program on the social competence of head start preschoolers. Soc Dev. 2006;15(3):501-19.

68. Wang H, Sekine M, Chen X, Yamagami T, Kagamimori S. Lifestyle at 3 years of age and quality of life (QOL) in first-year junior high school students in Japan: Results of the Toyama Birth Cohort Study. Qual Life Res. 2008;17(2):257-65.

69. Vella SA, Cliff DP, Magee CA, Okely AD. Associations between sports participation and psychological difficulties during childhood: A two-year follow up. J Sci Med Sport. 2015;18(3):304-9.

70. Irwin JD, Johnson AM, Vanderloo LM, Burke SM, Tucker P. Temperament and Objectively Measured Physical Activity and Sedentary Time among Canadian Preschoolers. Prev Med Rep. 2015;2:598-601.

71. Yu ML, Ziviani J, Baxter J, Haynes M. Time use differences in activity participation among children 4–5 years old with and without the risk of developing conduct problems. Res Dev Disabil. 2012;33(2):490-8.

72. Yu BN, Protudjer JLP, Anderson K, Fieldhouse P. Weight Status and Determinants of Health: In Manitoba Children and Youth. Can J Diet Pract Res. 2010;71(3):115-21.

73. Fliek L, Daemen E, Roelofs J, Muris P. Rough-and-tumble play and other parental factors as correlates of anxiety symptoms in preschool children. J Child Fam Stud. 2015; 24(9):2795-804.

74. Lindsay H, Brussoni M. Injuries and helmet use related to non-motorized wheeled activities among pediatric patients. Chronic Dis Inj Canada. 2014;34(2-3):74-81.

75. Mavilidi M-F, Okely AD, Chandler P, Cliff DP, Paas F. Effects of integrated physical exercises and gestures on preschool children’s foreign language vocabulary learning. Educ Psychol Rev. 2015;27(3):413-26.

76. Kirk SM, Vizcarra CR, Looney EC, Kirk EP. Using physical activity to teach academic content: A study of the effects on literacy in head start preschoolers. Early Child Educ J. 2014;42(3):181-9.

77. Kirk SM, Kirk EP. Sixty month of physical activity per day included within preschool academic lessons improves early literacy. J Sch Health. 2016;86(3):155-63.

78. Zachopoulou E, Trevlas E, Konstadinidou E, Group APR. The design and implementation of a physical education program to promote children’s creativity in the early years. Int J Early Years Educ. 2006;14(3):279-94.

79. Webster EK, Wadsworth DD, Robinson LE. Preschoolers’ time on-task and physical activity during a classroom activity break. Pediatr Exerc Sci. 2015;27(1):160-7.

80. Palmer KK, Miller MW, Robinson LE. Acute exercise enhances preschoolers’ ability to sustain attention. J Sport Exerc Psychol. 2013;35(4):433-7.

81. Holmes RM, Pellegrini AD, Schmidt SL. The effects of different recess timing regimens on preschoolers' classroom attention. Early Child Dev Care. 2006;176(7):735-43.

82. Kolpakov V, Bespalova T, Tomilova E, Larkina NY, Mamchits E, Chernogrivova M, et al. Functional reserves and adaptive capacity of subjects with different levels of habitual physical activity. Human Physiol. 2011;37(1):93-104.

83. Specker BL, Mulligan L, Ho M. Longitudinal study of calcium intake, physical activity, and bone mineral content in infants 6–18 months of age. J Bone Miner Res. 1999; 14(4):569-76.

84. Xu H, Zhao Z, Wang H, Ding M, Zhou A, Wang X, et al. Bone mineral density of the spine in 11,898 Chinese infants and young children: A cross-sectional study. PloS One. 2013;8(12):e82098.

85. Jazar AS, Takruri HR, Khuri-Bulos NA. Vitamin D status in a sample of preschool children aged from 1 to 6 years visiting the pediatrics clinic at Jordan University hospital. J Med J. 2012;45(4):308-16.

86. Kensarah OA, Jazar AS, Azzeh FS. Hypovitaminosis D in healthy toddlers and preschool children from Western Saudi Arabia. Int J Vit Nutr Res. 2015;85:50-60.

87. Specker BL, Johannsen N, Binkley T, Finn K. Total body bone mineral content and tibial cortical bone measures in preschool children. J Bone Miner Res. 2001;16(12):2298-305.

88. Harvey N, Cole Z, Crozier S, Kim M, Ntani G, Goodfellow L, et al. Physical activity, calcium intake and childhood bone mineral: A population-based cross-sectional study. Osteoporos Int. 2012;23(1):121-30.

89. Herrmann D, Buck C, Sioen I, Kouride Y, Marild S, Molnár D, et al. Impact of physical activity, sedentary behaviour and muscle strength on bone stiffness in 2–10-year-old children-cross-sectional results from the IDEFICS study. Int J Behav Nutr Phys Act. 2015;12:112.

90. Scheffler C, Ketelhut K, Mohasseb I. Does physical education modify the body composition?—Results of a longitudinal study of pre-school children. Anthropologischer Anzeiger. 2007:193-201.

91. Wilson DK, Klesges LM, Klesges RC, Eck LH, Hackett-Renner CA, Alpert BS, et al. A prospective study of familial aggregation of blood pressure in young children. J Clin Epidemiol. 1992;45(9):959-69.

92. Jiménez-Pavón D, Konstabel K, Bergman P, Ahrens W, Pohlabeln H, Hadjigeorgiou C, et al. Physical activity and clustered cardiovascular disease risk factors in young children: A cross-sectional study (the IDEFICS study). BMC Med. 2013;11:172.

93. Damashek A, Kuhn J. Toddlers’ unintentional injuries: The role of maternal-reported paternal and maternal supervision. J Pediatr Psychol. 2012:jss113.

94. Clark EM, Ness AR, Tobias JH. Vigorous physical activity increases fracture risk in children irrespective of bone mass: A prospective study of the independent risk factors for fractures in healthy children. J Bone Miner Res. 2008;23(7):1012-22.

95. Hutchison BL, Thompson JM, Mitchell EA. Determinants of nonsynostotic plagiocephaly: A case-control study. Pediatr, 2003;112(4):e316-22.

96. van Vlimmeren LA, van der Graaf Y, Boere-Boonekamp MM, L'Hoir MP, Helders PJ, Engelbert RH. Risk factors for deformational plagiocephaly at birth and at 7 weeks of age: A prospective cohort study. Pediatr, 2007;119(2):e408-18.
